# Synthesis and Antimicrobial Activity of a New Series of Thiazolidine-2,4-diones Carboxamide and Amino Acid Derivatives

**DOI:** 10.3390/molecules25010105

**Published:** 2019-12-27

**Authors:** Rakia Abd Alhameed, Zainab Almarhoon, Sarah I. Bukhari, Ayman El-Faham, Beatriz G. de la Torre, Fernando Albericio

**Affiliations:** 1 Department of Chemistry, College of Science, King Saud University, P.O. Box 2455, Riyadh 11451, Saudi Arabia; Roki.ahmed@yahoo.com (R.A.A.); zalmarhoon@ksu.edu.sa (Z.A.); 2 Department of Pharmaceutics, College of Pharmacy, King Saud University, P.O. Box 4584, Riyadh 11451, Saudi Arabia; sbukhari@ksu.edu.sa; 3 Chemistry Department, Faculty of Science, Alexandria University, P.O. Box 426, Ibrahimia, Alexandria 12321, Egypt; 4 KwaZulu-Natal Research Innovation and Sequencing Platform (KRISP), School of Laboratory Medicine and Medical Sciences, College of Health Sciences, University of KwaZulu-Natal, Durban 4041, South Africa; garciadelatorreb@ukzn.ac.za; 5 Peptide Science Laboratory, School of Chemistry and Physics, University of KwaZulu-Natal, Durban 4001, South Africa; 6 CIBER-BBN, Networking Centre on Bioengineering, Biomaterials and Nanomedicine, and Department of Organic Chemistry, University of Barcelona, Martí i Franqués 1-11, 08028 Barcelona, Spain

**Keywords:** OxymaPure, thiazolidine-2,4-dione carboxamide, thiazolidine-2,4-dione amino acid ester, antibacterial activity

## Abstract

Novel thiazolidine-2,4-dione carboxamide and amino acid derivatives were synthesized in excellent yield using OxymaPure/*N,N′*-diisopropylcarbodimide coupling methodology and were characterized by chromatographic and spectrometric methods, and elemental analysis. The antimicrobial and antifungal activity of these derivatives was evaluated against two Gram-positive bacteria (*Staphylococcus aureus* and *Bacillus subtilis*), two-Gram negative bacteria (*Escherichia coli* and *Pseudomonas aeruginosa*), and one fungal isolate (*Candida albicans).* Interestingly, several samples demonstrated weak to moderate antibacterial activity against Gram-negative bacteria, as well as antifungal activity. However, only one compound namely, 2-(5-(3-methoxybenzylidene)-2,4-dioxothiazolidin-3-yl)acetic acid, showed antibacterial activity against Gram-positive bacteria, particularly *S. aureus*.

## 1. Introduction

The underlying goal for research into medicinal chemistry is to discover new products with greater biological activity, achieving a low number of by-products during the synthesis and low toxicity of both the intermediates and the final products. Accordingly, considerable attention has been devoted to thiazolidinedione derivatives (TZDs), both from a synthetic point of view and biological applications [1,2,3,4,5,6]. In this regard, TZDs have been used as the following: antibacterial and antifungal agents [7,8,9,10,11,12]; anti-inflammatory drugs [13,14,15,16]; aldose reductase inhibitors [17,18]; and anticancer [19,20,21,22,23,24,25], antiplasmodial inhibitors [26], and antidiabetic agents [27,28,29,30,31,32]. Consequently, TZDs have become a pharmacologically important group of heterocyclic compounds and the object of great interest as precursors of novel drugs.

A survey of the literature reveals that the inclusion of the substituted aromatic ring at the *ortho* position, as well as substituents at the *meta* position, is required to enhance the biological activity of compounds [33,34]. Regarding the thiazolidinedione ring, substitution at the third position confers antimicrobial properties, especially when chloro, bromo, hydroxyl, and nitro groups are attached to the aromatic moiety [34]. Here we prepared a new series of thiazolidine-2,4-dione carboxamide and amino acid derivatives and characterized their antibacterial activity against Gram-positive, Gram-negative bacteria and fungal as well.

## 2. Results and Discussion

### 2.1. Chemistry

**Scheme 1:**
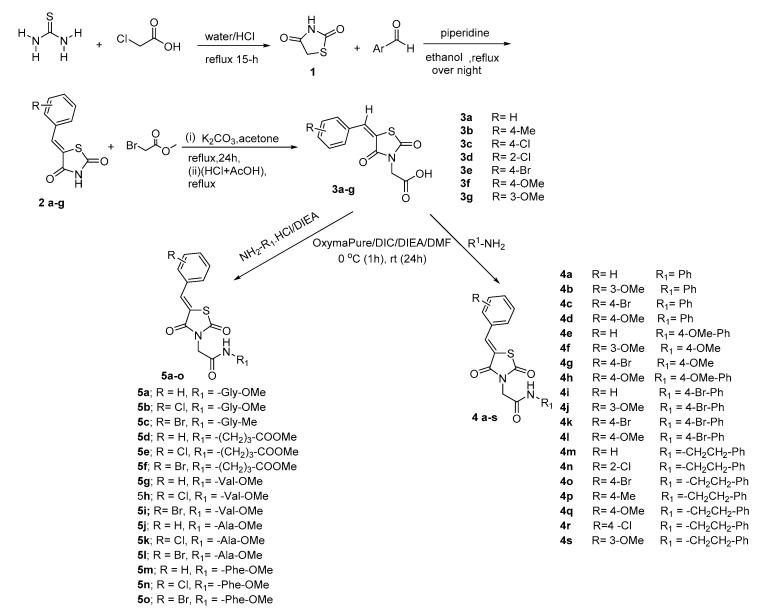
Synthesis of 2,4-dioxothiazolidine-carboxamide and amino acid derivatives derivatives.

2-(5-Arylidene-2,4-dioxothiazolidine-3-yl)acetic acids **3a**–**g** were synthesized as outlined in Scheme 1, where TZD **1** was prepared following the reported strategy [35] with minor modifications. TZD solution was treated with various appropriate aldehydes via refluxing in ethanol for 24 h in the presence of piperidine as a catalyst to afford compounds **2a**–**g**. Furthermore, reaction of **2a**–**g** with ethyl 2-bromoacetate in acetone in the presence of potassium carbonate as a base, followed by acidic hydrolysis using acetic acid-HCl, furnished target acid derivatives **3a**–**g**. Some spectral data are reported in [7,13,15] and other spectra are given in the Supplementary Materials.

The acid derivatives **3a**–**g** were reacted with different amines in DMF and in the presence of ethyl (hydroxyimino)cyanoacetate [OxymaPure] and *N*,*N′*-diisopropylcarbodiimide (DIC) as a coupling cocktail [36,37] to give the carboxamide derivatives **4a**–**s** in >90% yield (Scheme 1). All the prepared derivatives were characterized by FT-IR, ^1^H, ^13^C-NMR techniques and elemental analysis.

It could be envisaged a synthetic scheme based first on the alkylation of the NH of the thiazolidine, followed by the amidation, and finally the condensation with the aldehyde as previously described for different thiazolidine based derivatives [38]. However, the strategy chosen herein facilitates excellent yields for all reactions due presumably to the solubility of all the intermediates in the corresponding solvents.

**Figure 1:**
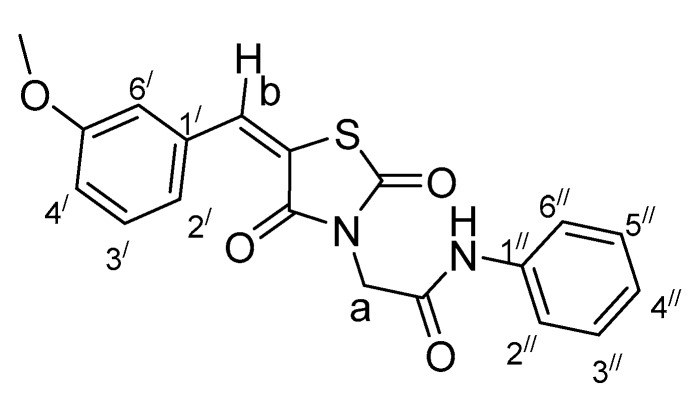
Structure of compound **4b**.

The ^1^H-NMR spectrum of **4b** as a prototype for the **4a**–**s** series (Figure 1) showed two singlet peaks at δ 3.80 and 4.49 integrated for the hydrogens of the methoxy group and the methylene group H*_a_*, respectively. In addition, multiple peaks at δ 7.07–7.53 represented nine aromatic protons, while the better to numbering it in the figure C*H*=C=S (H*_b_*) and NH proton appeared at δ 7.95 and 10.38 ppm, respectively.

The ^13^C-NMR spectrum of **4b** showed that two peaks at δ 44.5 and 55.8 belonged to the methylene group (CH_2_-CO-N-) and (OCH_3_), respectively, twelve aromatic carbon peaks at δ 116.0, 117.2, 119.6, 121.8, 122.4, 124.2, 129.3, 131.0, 134.2, 134.6, 138.8, and 160.1. In addition, while three peaks appear at δ 164.2, 165.7 and 167.5, were attributed to (CO).

Reaction of **3a**–**g** was performed with amino acid esters hydrochloride using OxymaPure/DIC as a coupling agent in the presence of 1 equiv. DIEA as a base to afford **5a**–**o** as shown in Scheme 1.

All the derivatives prepared were characterized by FT-IR, ^1^H-NMR, ^13^C-NMR techniques and elemental analysis.

**Figure 2:**
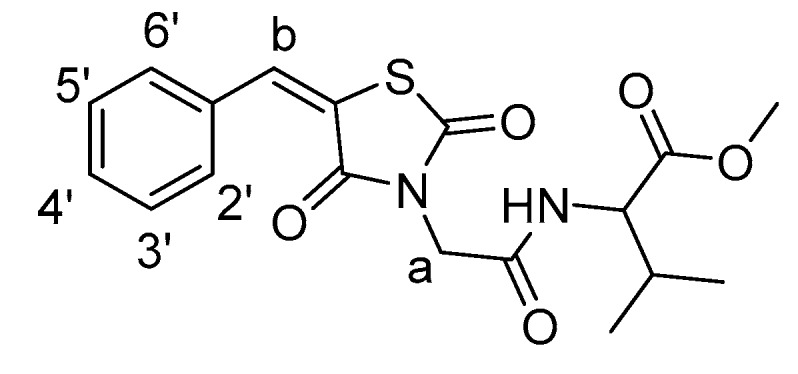
Structure of compound **5g**.

The ^1^H-NMR spectrum of **5g** (Figure 2) as a prototype for the **5a**–**o** series showed a singlet peak at δ 0.84 ppm, which integrated six protons for 2CH_3_ (valine residue), and one broad singlet peak at δ 2.00, which integrated one proton for CH(CH_3_)_2_ (valine residue), singlet at δ 3.61 represents 3H for OCH_3_ and two singlet peaks appeared at δ 4.18 and 4.34, related to NHCHCO (valine residue) and NCH_2_CO, H*_a_*), respectively. Multiple peaks belonging to five aromatic protons appeared at δ 7.51–7.60, while the CH=C-S (H*_b_*) and NH protons appeared at δ 7.92 and 8.64 ppm, respectively.

The ^13^C-NMR spectrum of **5g** displayed six peaks at δ 121.4, 129.8, 130.6, 131.2, 133.3, and 133.8 related to the aromatic carbons, in addition to four peaks at δ 165.6, 165.8, 167.4, 172.1 corresponding to four (CO), and also six peaks at δ 18.5, 19.3, 30.6, 43.6, 52.2 and 58.0, attributed to three carbons, (CH(CH_3_)_2_), (NCH_2_CO), (COOCH_3_), and (CH CO), respectively.

### 2.2. Biology

Using the well diffusion technique, we examined the in vitro antibacterial activity of the synthesized compounds against two Gram-positive bacteria, namely *Staphylococcus aureus* (ATCC 29213) and *Bacillus subtilis* (ATCC 10400), two Gram-negative bacteria, namely *Escherichia coli* (ATCC 25922) and *Pseudomonas aeruginosa* ATCC 27853, and one fungal strain of *Candida albicans* (ATCC 10231).

Antimicrobial activity was determined by measuring the inhibition zone around each well in mm (Table 1). Inhibition zones above 8 mm indicated that the micro-organism was susceptible to the specific chemical compound used. Data were compared to the positive control standard antibiotic discs of an Impenem (10 µg), Sulfamethzole trioxamethoprim for the bacterial isolates, and Fluconazole for the *Candida* isolate. Tests were repeated three times and the average of the inhibition zone was recorded in Table 1.

Table 1 showed that the tested micro-organisms had variable sensitivity and susceptibility to the chemical compounds. Indeed, the antimicrobial assay revealed that most of compounds tested had no or negligible activity against *S. aureus* and *B. subtilis* with the exception of some acid derivatives (**3a**, **3b**, and **3g**)**.** This observation could be explained by the difference in the cell wall structure of Gram-positive and Gram-negative bacteria or it may be due to the charges and the kinetics of the chemical compounds, which can damaged the bacterial cell wall via electrostatic interactions, as previously reported by Azevedo et al. [39].

Regarding the series **3a**–**g**, the presence of methoxy and chloro groups and their positions has a great impact on the biological activity. Whereas the methoxy group at the *meta* position (compound **3g**) enhanced the activity more than the same group at the *para* position (compound **3f**) also chloro at the *ortho* position (compound **3d**) showed more activity than the same group at the *para* position as in **3c**. The latter showed only minor activity against Gram-negative bacteria (*Ps. aeruginosa*), achieving an inhibition zone of 10 mm. The unsubstituted derivative **3a** and the derivative with chloro at the *ortho* position **3d** showed moderate activity against *C. albicans* as shown in Table 1.

The two series of 2,4-dioxothiazolidine carboxamides **4a**–**s** and 2,4-dioxothiazolidine amino acid ester derivatives **5a**–**o** showed no activity against Gram-positive bacteria. While some derivatives showed weak activity against Gram-negative bacteria (*E. coli*), compounds **4s** from the series **4a**–**s** showed weak activity, while compounds **5g**, **5h**, **5i**, **5n** and **5o** from series **5a**–**o** showed moderate activity. Most of the compounds from series **4a**–**s**, especially derivatives with the ethyl morpholine moiety **4m**–**s** showed activities against Gram-negative bacteria (*Ps. aeruginosa*), with inhibition zones ranging from 10–14 mm (Table 1). The presence and position of the bromo in the carboxamide series **4a**–**s** had a remarkable effect on the antifungal activity, as shown in **4c**, **4k**, and **4i** (Table 1). On the other hand, the antifungal activity increased as the number of the bromo atom increased in the molecules as shown in **4k** vs. **4c** (15 mm vs. 12 mm, respectively). In contrast, increasing the methoxy group as in **4h** had no effect on activity against *C. albicans* as shown in Table 1. However, the presence of bromo beside the methoxy group **4g** showed good antifungal activity (14 mm).

For the **5a**–**o** series, utilizing glycinate, alaninate, butanoate, and phenylalaninate without any substitution on the benzene ring **5a**, **5j**, and **5d** did not lead to microbial activity, except **5m,** which showed activity against *C. albicans* (12 mm). However, valinate and its derivatives **5g**–**i** did show inhibitory activity. Glycinate derivatives **5a**–**c** showed no activity against Gram-positive or Gram-negative bacteria. However, they exerted antifungal activity when halogen present in the molecule **5b** and **5c** (15 mm and 13 mm**,** respectively)**.** The alaninate derivatives with halogen substituent **5k**–**l** demonstrated activity against *C. albicans,* and the derivative with chloro **5k** (18 mm) was more active than the other derivatives. In addition, **5k** showed activity (7 mm and 13 mm) against two-Gram negative bacteria (*E. coli* and *Ps. aeruginosa*, respectively).

Butanoate derivatives **5d**–**f** showed no antimicrobial activity, except the derivative with a chlorine atom **5e,** which showed minor activity against *Ps. aeruginosa* (12 mm). All valinate derivatives **5g**–**i** recorded activity against gram-negative bacteria and antifungal activity, except **5i** with a bromine substitution, which did not exert antifungal activity.

Compound **5o** showed antimicrobial activity against *E. coli, Ps. Aeruginosa* and *C.albicans,* respectively (10 mm, 12 mm, and 16 mm). These promising new compounds lend themselves to minor structural modifications to enhance their activity or may find applications in other pharmaceutical fields.

## 3. Materials and Methods

### 3.1. Materials and Methods

All the starting materials, chemicals, reagents and solvents were purchased from commercial known reputable sources and were used without further purification. TLC (silica gel 60-F254 protected aluminum sheets) was used to monitor the reactions. All melting points were performed in open capillary tubes using a Gallenkamp melting point apparatus (Sigma-Aldrich Chemie GmbH, Taufkirchen, Germany) and are uncorrected. FTIR spectra were recorded on a Shimadzu 8201 PC FTIR spectrophotometer (Shimadzu, Ltd., Chiyoda-ku, Tokyo, Japan). Elemental analyses were performed on a Perkin-Elmer 2400 elemental analyzer (PerkinElmer, Inc., Waltham, MA, USA), and the values found were within ±0.3% of the theoretical values.^1^H- and ^13^C-NMR spectra were recorded on a Varian-Agilent-NMR 600 MHz spectrometer (Varian, Inc., Palo Altro, CA, USA).

UPLC-MS conditions were as follows: instrument: Waters Acquity UPLC system (Waters Corp., Milford, MA, USA) and a triple quadrupole (TQD) mass spectrometer equipped with a Z-electrospray interface. Parameters of the electrospray ionization source were as follows: capillary voltage: 3.0 kV; cone voltage: 28 V; desolvation gas: nitrogen with flow 800 L/h; cone gas: nitrogen with flow 70 L/h; source temperature: 120 °C; and desolvation temperature: 300 °C. Analysis was done in full scan mode with positive ionization in the mass range of 50–850 Da. The sample solutions were directly infused to the ion source at a flow rate of 10 µL/min. Data acquisition and processing were done using Waters MassLynx software.

### 3.2. General Procedure for the Synthesis of 2,4-Dioxothiazolidine Acid Derivatives

2,4-Dioxothiazolidine acid derivatives were prepared in three steps following the reported method [35,40]. A solution of previously prepared TZD **1** was treated with various appropriate aldehydes via refluxing in ethanol for 24 h in the presence of piperidine as a catalyst. The reaction mixture was poured into water, followed by acidification with acetic acid to afford compounds **2a**–**g**. Then a mixture of **2a**–**g** (1 mmol) and ethyl 2-bromoacetate (2 mmol) was refluxed for 24 h in acetone in the presence of potassium carbonate (2 mmol) to furnish the target product as a white solid after evaporation of the solvent. The crude product was used directly in the next step for the preparation of the free carboxylic acid derivatives **3a**–**g**, where the solid product was refluxed with glacial acetic acid and HCl at a ratio of 4:1 for 2 h to afford the pure (2,4-dioxothiazolidine-3-yl)-acetic acid derivatives **3a**–**g** after evaporation of the solvents and crystallization with ethanol. The spectral data for **3a**, **3f**, and **3g** are in good agreement with previously reported ones [7,13,15] and other spectra in the Supplementary Materials.

#### 3.2.1. 2-(5-(4-Methylbenzylidene)-2,4-dioxothiazolidin-3-yl)acetic Acid (**3b**)

The product was obtained as light-yellow crystals in 96% yield, mp: 226–228 °C. IR (KBr, cm^−1^): 2950 (CH-aliphatic); 1733, 1685, 1600 (CO); ^1^H-NMR (DMSO-*d*_6_, δ ppm): 2.34 (3H, s, CH), 4.36 (2H, s, CH_2_COOH); 7.33 (2H, d, *J =* 6.6 Hz, H_3′_ & H_5′_); 7.51 (2H, d, *J =* 7.2 Hz, H_2′_ & H_6′_), 7.92 (1H, s, CH=C). ^13^C-NMR (DMSO-*d*_6_, δ ppm): 21.6 (CH_3_); 42.7 (CH_2_-COOH); 119.8, 130.5, 130.7, 134.4, 141.7; 165.5, 167.4, 168.4 (CO). Anal. Calc. for C_13_H_11_NO_4_S (277.3): C, 56.31; H, 4.00; N, 5.05; Found C, 56.44; H, 4.12; N, 5.26. LC/MS (ESI): 278.32 [M + H]^+^.

#### 3.2.2. 2-(5-(4-Chlorobenzylidene)-2,4-dioxothiazolidin-3-yl)acetic Acid (**3c**)

The product was obtained as light-yellow crystals in yield 92%, mp: 250–252 °C. IR (KBr, cm^−1^): 3008 (CH-aromatic); 1738, 1690 & 1607 (CO); ^1^H-NMR (DMSO-*d*_6_, δ ppm): 4.42 (2H, s, CH_2_-COOH); 7.59–8.00 (4H, m, aromatic protons), 8.03 (1H, s, CH=C); ^13^C-NMR (DMSO-*d*_6_, δ ppm): 42.2 (CH_2_-COOH); 121.3, 129.3, 131.5, 131.7, 132.4, 35.3; 164.8, 166.5, 167.8 (CO). Anal. Cal. For C_12_H_8_ClNO_4_S (297.7): C, 48.40; H, 2.70; N, 4.70; Found C, 48.61; H, 2.83; N, 4.81. LC/MS (ESI): 298.72 [M + H]^+^.

#### 3.2.3. 2-(5-(2-Chlorobenzylidene)-2,4-dioxothiazolidin-3-yl)acetic Acid (**3d**)

The product was obtained as white crystals in 90% yield, mp: 243–245 °C. IR (KBr, cm^−1^): 3064 (CH-aromatic); (2940) CH-aliphatic; 1490 (C=C); 1722, 1691, 1608 (CO); ^1^H-NMR (DMSO-*d*_6_, δ ppm): 3.78 (3H, s, OCH_3_); 4.43 (2H, s, CH_2_COOH); 7.55–7.69 (4H, m, aromatic protons); 8.09 (1H, s, CH=C); ^13^C-NMR (DMSO-*d*_6_, δ ppm): 42.0 (CH_2_COOH); 124.1, 128.0, 128.8, 130.8, 130.6, 132.0; 134.4 (CH=C); 164.3, 166.3, 167.6 (CO). Anal. Cal. for C_12_H_8_ClNO_4_S (297.7): C, 48.41; H, 2.71; N, 4.70; Found: C, 48.65; H, 2.84; N, 4.95.

#### 3.2.4. 2-(5-(4-Bromobenzylidene)-2,4-dioxothiazolidin-3-yl)acetic Acid (**3e**)

The product was obtained as yellowish white crystals in 94% yield, mp: 260–262 °C. IR (KBr, cm^−1^): 2948 (CH-aliphatic); 1696, 1606 (CO); ^1^H-NMR (DMSO-*d*_6_, δ ppm): 4.37 (2H, s, CH_2_COOH); 7.56, (2H, d, *J* = 6.6 Hz, H_2′_ & H_6′_); 7.73 (2H, d, *J* = 6 Hz, H_3′_ & H_5′_); 7.95 (1H, s, CH=C); ^13^C-NMR (DMSO-*d*_6_, δ ppm): 42.8 (CH_2_-COOH); 121.9, 124.9, 132.4, 132.8, 133.1; 165.3, 167.08, 168.4 (CO). Anal. Calc. for C_12_H_8_BrNO_4_S (342.16): C, 42.12; H, 2.36; N, 4.09; Found C, 42.45; H, 2.59; N, 4.25.

### 3.3. General Procedure for the Synthesis of 2,4-Dioxothiazolidine Carboxamide Derivatives ***4a**–**s***

A mixture of an acid **3a**–**g** (1 mmol), and OxymaPure (1 mmol) was dissolved in 5 mL DMF at 0 °C, followed by dropwise addition of DIC (1.1 mmol) at 0 °C. The reaction mixture was preactivated for 5 min and then 1 mmol of an amine (aniline, *p*-OMe aniline, *p*-Br aniline and 4-(2-aminoethyl) morpholine) was added dropwise at the same temperature. After that, the mixture was stirred at 0 °C for 1 h and then left overnight under stirring at rt. The progress of the reaction was followed by TLC (ethyl acetate-hexane; 4:6 or MeOH-CHCl_3_; 1:9). Excess water was added, and the mixture was extracted with ethyl acetate three times (3 × 20 mL), followed by washing with 1 N HCl (2 × 10 mL), a saturated solution of Na_2_CO_3_ (2 × 10 mL), and a saturated solution of NaCl (10 mL). It was then dried over anhydrous MgSO_4_ for 20 min (when there was precipitation after pouring into water, the final product was isolated by normal filtration and 3 washings with water).

#### 3.3.1. 2-(5-Benzylidene-2,4-dioxothiazolidine-3-yl)*-N-*phenylacetamide (**4a**)

The product was obtained as a white powder from ethanol in 97% yield, mp: 256–258 °C. IR (KBr, cm^−1^): 3278 (N-H); 1748, 1694 & 1662 (C=O); ^1^H-NMR (DMSO-*d*_6_, δ ppm): 4.50 (2H, s, CH_2_CO); 7.06–7.64 (10H, m, 2-Ph); 7.98 (1H, s, CH=C); 10.38 (s, 1H, NH); ^13^C-NMR (DMSO-*d*_6_, δ ppm): 44.5 (CH_2_CONH), 119.6, 121.4, 124.2, 129.3, 129.9, 130.6, 131.3, 133.3, 134.1, 138.8; 164.2, 165.7, 167.6 (CO). Anal.Calc for C_18_H_14_N_2_O_3_S (338.38): C, 63.89; H, 4.17; N, 8.28; Found: C, 64.02; H, 4.31; N, 8.42. LC/MS (ESI): 339.61 [M + H]^+^.

#### 3.3.2. 2-(5-(3-Methoxybenzylidene)-2,4-dioxothiazolidine-3-yl)*-N-*phenylacetamide (**4b**)

The product was obtained as a white powder from ethanol in 96% yield, mp: 227–229 °C. IR (KBr, cm^−1^): 3271 (NH); 1745, 1667 (CO); ^1^H-NMR (DMSO-*d*_6_, δ ppm): 3.80 (3H, s, OCH_3_); 4.50 (2H, s, CH_2_); 7.07–7.53 (9H, m, aromatic protons); 7.95 (1H, s, CH=C); 10.38 (1 H, s, NH); ^13^C-NMR (DMSO-*d*_6_, δ ppm): 44.5 (CH_2_CONH); 55.8 (OCH_3_); 116.0, 117.2, 119.6, 121.8, 122.4, 124.2, 129.3, 131.0, 134.0, 134.6, 138.8, 160.1; 164.2, 165.7, 167.5 (CO). Anal. Calc for C_19_H_16_N_2_O_4_S (368.41): C, 61.94; H, 4.38; N, 7.60; Found: C, 61.82; H, 4.44; N, 7.82. LC/MS (ESI): 369.54 [M + H]^+^.

#### 3.3.3. 2-(5-(4-Bromobenzylidene)-2,4-dioxothiazolidine-3-yl)*-N-*phenylacetamide (**4c**)

The product was obtained as a white powder from ethanol in 96% yield, mp: 273–275 °C. IR (KBr, cm^−1^): 3296 (NH); 1748, 1694, 1668 (CO); 1605 (C=C aromatic); ^1^H-NMR (DMSO-*d*_6_, δ ppm): 4.51 (2H, s, CH_2_-CO); 7.05 (1H, s, H_4″_); 7.29 (2H, d, *J =* 3.6 Hz, H_3″_ & H_5″_); 7.55 (4H, t, *J =* 7.2 Hz, H_2′_, H_6′_, H_3′_ & H_5′_); 7.71 (2H, s, H_2″_ & H_6″_); 7.93 (1H, s, CH=C); 10.4 (1H, s, NH); ^13^C-NMR (DMSO-*d*_6_, δ ppm): 44.5 (CH_2_CONH); 119.6, 122.2, 124.2, 124.8, 129.3, 132.4, 132.5, 132.8, 138.8; 164.1, 165.6, 167.3 (3CO). Anal. Calc for C_18_H_13_BrN_2_O_3_S (417.28): C, 51. 81; H, 3.14; N, 6.71; Found: C, 51.98; H, 3.33; N, 6.91. LC/MS (ESI): 418.41 [M + H]^+^.

#### 3.3.4. 2-(5-(4-Methoxybenzylidene)-2,4-dioxothiazolidine-3-yl)*-N-*phenylacetamide (**4d**)

The product was obtained as a white powder from ethanol in 98% yield, mp: 249–251 °C. IR (KBr, cm^−1^): 3297 (NH); 1742, 1679 and 1594 (CO); ^1^H-NMR (DMSO-*d*_6_, δ ppm): 3.81 (3H, s, OCH_3_); 4.49 (2H, s, CH_2_); 7.08 (3H, dd, *J* = 6.6 Hz,3.6 Hz H_4″_, H_3′_ & H_5′_); 7.30 (2H, d, *J =* 4.2 Hz, H_3″_ & H_5″_); 7.53 (2H, s, H_2″_ & H_6″_); 7.60 (2H, s, H_2′_ & H_6′_); 7.92 (1H, s, CH=C); 10.38 (1H, s, NH); ^13^C-NMR (DMSO-*d*_6_, δ ppm): 44.4 (CH_2_CO NH); 56.0 (OCH_3_); 115.5, 118.1, 119.6, 124.1, 125.7, 129.3, 132.8, 134.0, 138.8, 161.7; 164.3, 165.9, 167.7 (CO). Anal. Calc for C_19_H_16_N_2_O_4_S (368.41): C, 61.94; H, 4.38; N, 7.60; Found: C, 61.77; H, 4.29; N, 7.81. LC/MS (ESI): 369.30 [M + H]^+^.

#### 3.3.5. 2-(5-Benzylidene-2,4-dioxothiazolidine-3-yl)*-N-*(4-methoxyphenyl)acetamide (**4e**)

The product was obtained as a white powder from ethanol in 97% yield, mp: 258–260 °C. IR (KBr, cm^−1^): 3280 (NH), 1745, 1694, 1658 (CO); ^1^H-NMR (DMSO-*d*_6_, δ ppm): 3.70 (3H, s, OCH_3_); 4.46 (2H, s, CH_2_CO); 6.87 (2H, d, *J =* 7.2 Hz, H_3″_ & H_5″_); 7.44 (2H, s, H_2″_ & H_6″_); 7.52 (3H, dd, *J* = 7.8, 6 Hz, H_3′_, H_5′_ & H_4′_); 7.64 (2H, s, H_2′_ & H_6′_); 7.97 (1H, s, CH=C); 10.24 (H, s, NH); ^13^C-NMR (DMSO-*d*_6_, δ ppm): 44.4 (CH_2_CONH); 55.6 (OCH_3_); 114.4, 121.2, 121.5, 129.9, 130.6, 131.3, 131.9, 133.3, 134.0, 155.9; 163.7, 165.8, 167.6 (3CO). Anal. Calc for C_19_H_16_N_2_O_4_S (368.41): C, 61.94; H, 4.38; N, 7.60; Found: C, 62.12; H, 4.55; N, 7.87. LC/MS (ESI): 369.41[M + H]^+^.

#### 3.3.6. 2-(5-(3-Methoxybenzylidene)-2,4-dioxothiazolidine-3-yl)*-N-*(4-methoxyphenyl)acetamide (**4f**)

The product was obtained as a white powder from ethanol in 97% yield, mp: 225–227 °C. IR (KBr, cm^−1^): 3339 (NH), 1749, 1694 & 1612 (CO); ^1^H-NMR (DMSO-*d*_6_, δ ppm): 3.70 (3H, s, OCH_3_); 3.80 (3H, s, OCH_3_); 4.46 (2H, s, CH_2_CO); 6.87 (2H, s, H_3″_ & H_5″_); 7.08 (1H, s, H_2′_); 7.20 (2H, s, H_6′_ & H_4′_), 7.44 (3H, t, *J* = 8.4 Hz, 12 Hz, H_2″_, H_5′_, H_6″_); 7.95 (1H, d, *J* = 1.2 Hz, CH=C); 10.23 (1H, s, NH); ^13^C-NMR (DMSO-*d*_6_, δ ppm): 44.3 (CH_2_CONH); 55.6, 55.7 (2OCH_3_); 114.4, 115.9, 117.1, 121.1, 121.8, 122.4, 130.9, 131.9, 133.9, 134.6, 155.9, 160.1; 163.6, 165.7, 167.5 (CO).Anal. Calc for C_20_H_18_N_2_OS (398.43): C, 60.29; H, 4.55; N, 7.03; Found: C, 60.55; H, 4.67; N, 7.27. LC/MS (ESI): 399.21 [M + H]^+^.

#### 3.3.7. 2-(5-(4-Bromobenzylidene)-2,4-dioxothiazolidine-3-yl)*-N-*(4-methoxyphenyl)acetamide (**4g**)

The product was obtained as a white powder from ethanol in 96% yield, mp: 270–272 °C. IR (KBr, cm^−1^): 3280 (NH), 1746, 1693, 1662 (CO); ^1^H-NMR (DMSO-*d*_6_, δ ppm): 3.69 (3H, s, OCH_3_); 4.46 (2H, s, CH_2_CO); 6.86 (2H, s, H_3″_ & H_5″_); 7.43 (2H, s, H_2″_ & H_6″_), 7.57 (2H, s, H_2′_ & H_6′_), 7.72 (2H, s, H_3′_ & H_5′_); 7.93 (1H, s, CH=C); 10.24 (1H, s, NH); ^13^C-NMR (DMSO-*d*_6_, δ ppm); 44.4 (CH_2_CONH); 55.6 (OCH_3_); 114.4, 121.2, 122.3, 124.8, 131.9, 132.4, 132.5, 132.8, 132.8, 155.9; 163.6, 165.6, 167.3 (CO). Anal. Calc for C_19_H_15_BrN_2_O_4_S (447.3): C, 51.02; H, 3.38; N, 6.26; Found: C, 51.39; H, 3.54; N, 6.43. LC/MS (ESI): 448.32 [M + H]^+^.

#### 3.3.8. 2-(5-(4-Methoxybenzylidene)-2,4-dioxothiazolidine-3-yl)*-N-*(4-methoxyphenyl)acetamide (**4h**)

The product was obtained as an off-white powder from ethanol in 98% yield, mp: 257–259 °C. IR (KBr, cm^−1^): 3270 (NH), 1740, 1687, 1668 (CO); ^1^H-NMR (DMSO-*d*_6_, δ ppm): 3.69 (3H, s, OCH_3_); 3.81 (3H, s, OCH_3_); 4.45 (2H, s, CH_2_); 6.87 (2H, s, H_3′_ & H_5′_); 7.09 (2H, s, H_2′_ & H_6′_); 7.44 (2H, s, H_3′_ & H_5′_); 7.60 (2H, s, H_2′_ & H_6′_); 7.91 (1H, s, CH=C); 10.23 (1H, s, NH); ^13^C-NMR (DMSO-*d*_6_, δ ppm); 44.3 (CH_2_CONH); 55.6, 56.0 (2OCH_3_); 114.4, 115.5, 118.1, 121.1, 125.8, 132.0, 132.8, 134.0, 155.9, 161.7; 163.8, 165.9, 167.7 (CO). Anal. Calc for C_20_H_18_N_2_O_5_S (398.43): C, 60.29; H, 4.55; N, 7.03. Found: C, 60.54; H, 4.66; N, 7.29. LC/MS (ESI): 399.62 [M + H]^+^.

#### 3.3.9. 2-(5-Benzylidene-2,4-dioxothiazolidine-3-yl)*-N-*(4-bromophenyl)acetamide (**4i**)

The product was obtained as a white powder from ethyl acetate-ethanol (2:1) in 95% yield, mp: 252–254 °C. IR (KBr, cm^−1^): 3278 (N-H); 1750, 1693, 1660 (C=O); ^1^H-NMR (DMSO-*d*_6_, δ ppm): 4.51 (2H, s, CH_2_); 7.50 (5H, s, -Ph proton); 7.54 (2H, d, *J* = 6 Hz, H_2″_ & H_6″_); 7.65 (2H, d, *J* = 7.2 Hz, H_2′_ & H_6′_); 7.98 (1H, s, CH=C); 10.52 (1H, s, NH); ^13^C-NMR (DMSO-*d*_6_, δ ppm); 44.5 (CH_2_CONH); 115. 8, 121.4, 121.6, 129.9, 130.7, 131.3, 132.2, 133.3, 134.1, 138.2; 164.5, 165.7, 167.5 (CO). Anal. Calc for C_18_H_13_BrN_2_O_3_S (417.28): C, 51.81; H, 3.14; N, 6.71; Found: C, 51.98; H, 3.23; N, 6.92. LC/MS (ESI): 418.51 [M + H]^+^.

#### 3.3.10. *N*-(4-Bromophenyl)-2-(5-(3-methoxybenzylidene)-2,4-dioxothiazolidine-3-yl) acetamide (**4j**)

The product was obtained as a white powder from ethyl acetate-ethanol (2:1) in yield, mp: 253–255 °C. IR (KBr, cm^−1^): 3330 (N-H); 1748, 1688, 1609 (CO); ^1^H-NMR (DMSO-*d*_6_, δ ppm): 3.79 (3H, s, OCH_3_); 4.50 (2H, s, CH_2_); 7.07–7.49 (8H, m, aromatic proton); 7.94 (1H, s, CH=C); 10.54 (1H, s, NH); ^13^C-NMR (DMSO-*d*_6_, δ ppm): 44.5 (CH_2_CONH); 55.7 (OCH_3_); 115.8, 116.0, 117.2, 121.6, 121.7, 122.4, 132.0, 132.2, 134.1, 134.6, 138.1, 160.1; 164.4, 165.64, 167.5 (CO). Anal. Calc for for C_19_H_15_BrN_2_O_4_S (447.30): C, 51.02; H, 3.38; N, 6.26; Found: C, 51.33; H, 3.51; N, 6.43. LC/MS (ESI): 448.21 [M + H]^+^.

#### 3.3.11. 2-(5-(4-Bromobenzylidene)-2,4-dioxothiazolidine-3-yl)*-N-*(4-bromophenyl)acetamide (**4k**)

The product was obtained as a white powder from ethyl acetate-ethanol (2:1) in 96% yield, mp: 286–288 °C. IR (KBr, cm^−1^): 3259 (NH); 1748 & 1691 (CO); ^1^H-NMR (DMSO-*d*_6_, δ ppm): 4.50 (2H, s, CH_2_CO); 7.49 (4H, s, H_2″_, H_3″_, H_5″_ & H_6″_); 7.57 (2H, s, H_2′_ & H_6′_), 7.73 (2H, s, H_3′_ & H_5′_), 7.94 (1H, s, CH=C); 10.53 (1 H, s, NH); ^13^C-NMR (DMSO-*d*_6_; δ ppm): 44.5 (CH_2_CO NH); 115.8, 121.6, 122.2, 124.8, 132.2, 132.4, 132.5, 132.8, 132.9, 138.1 (C-sp^2^); 164.4, 165.6, 167.3 (CO). Anal. Calc for C_18_H_12_Br_2_N_2_O_3_S (496.17): C, 43.57; H, 2.44; N, 5.65; Found: C, 43.71; H, 2.61; N, 5.80. LC/MS (ESI): 497.21 [M + H]^+^.

#### 3.3.12. *N*-(4-Bromophenyl)-2-(5-(4-methoxybenzylidene)-2,4-dioxothiazolidine-3-yl)acetamide (**4l**)

The product was obtained as a yellowish white powder from ethyl acetate-ethanol (2:1) in 98% yield, mp: 260–262 °C. IR (KBr, cm^−1^): 3267 (NH); 1740 & 1689 (C=O); ^1^H-NMR (DMSO-*d*_6_, δ ppm): 3.81 (3H, s, OCH_3_); 4.49 (2H, s, CH_2_CO); 7.09 (2H, d, *J =* 6.6 Hz, H_3′_ & H_5′_); 7.48 (4H, d, *J =* 7.8 Hz, H_2″_, H_3″_, H_5″_, H_6″_), 7.60 (2H, s, H_2′_ & H_6′_); 7.92 (1H, s, CH=C); 10.52 (1H, s, NH); ^13^C-NMR (DMSO-*d*_6_, δ ppm): 44.4 (CH_2_CONH); 56.0 (OCH_3_); 115.5, 115.8, 118.0, 121.5, 125.7, 132.1, 132.8, 134.1, 138.2, 161.8; 164.5, 165.8, 167.6 (CO). Anal. Calc for C_19_H_15_BrN_2_O_4_S (447.30): C, 51.02; H, 3.38; N, 6.26; Found: C, 51.22; H, 3.47; N, 6.50. LC/MS (ESI): 448.51 [M + H]^+^.

#### 3.3.13. 2-(5-Benzylidene-2,4-dioxothiazolidine-3-yl)*-N-*(2-morpholinoethyl)acetamide (**4m**)

The product was obtained as a white powder from ethanol in 89% yield, mp: 180–182 °C. IR (KBr, cm^−1^): 3308 (NH); 2941 (CH-aliphatic); 1748, 1691, 1658 (CO); ^1^H-NMR (DMSO-*d*_6_, δ ppm): 2.33 (6H, d, *J* = 6 Hz), (CH_2_NCH_2_ & CH_2_CH_2_N); 3.18 (2H, s, NHCH_2_CH_2_); 3.54 (4H, s, CH_2_OCH_2_); 4.25 (2H, s, NCH_2_CO); 7.49 (5H, m, -Ph proton); 7.93 (1H, s, CH=C); 8.21 (1H, s, NH); ^13^C-NMR (DMSO-*d*_6_, δ ppm): 36.6 (NHCH_2_); 43.9 (CH_2_CO); 53.7 (CH_2_NCH_2_); 57.6 (CH_2_CH_2_N); 66.6 (CH_2_OCH_2_); 121.6, 129.9, 130.6, 131.2, 133.3, 133.7; 165.4, 165.7, 167.5 (CO). Anal. Calc for C_18_H_21_N_3_O_4_S (375.44): C, 57.58; H, 5.64; N, 11.19; Found: C, 57.69; H, 5.74; N, 11.33. LC/MS (ESI): 376.62 [M + H]^+^.

#### 3.3.14. 2-(5-(2-Chlorobenzylidene)-2,4-dioxothiazolidine-3-yl)-N-(2-morpholinoethyl)acetamide (**4n**)

The product was obtained as a white powder from ethanol in 95% yield, mp: 177–179 °C. IR (KBr, cm^−1^): 3307 (NH); 2958 (CH-aliphatic); 1751, 1703 and 1657 (CO); ^1^H-NMR (DMSO-*d*_6_, δ ppm): 2.33 (6H, s, CH_2_NCH_2_ & CH_2_CH_2_N); 3.18 (2H, s, NHCH_2_CH_2_); 3.54 (4H, s, CH_2_OCH_2_; 4.26 (2H, s, NCH_2_C=O); 7.51 (2H, s, H_4′_ & H_5′_);7.59 (1H, d, *J* = 9 Hz, H_6′_);7.63 (1H, s, H_3′_); 8.03 (1H, s, CH=C); 8.24 (1H, s, NH); ^13^C-NMR (DMSO-*d*_6_, δ ppm): 36.6 (NH CH_2_); 44.0 (CH_2_-CO); 53.7 (CH_2_NCH_2_); 57.6 (CH_2_CH_2_N); 66.6 (CH_2_OCH_2_); 125.3, 128.6, 128.8, 129.4, 130.8, 131.3, 132.6, 134.9; 165.3, 165.3, 167.3 (CO). Anal. Calc for C_18_H_20_ClN_3_O_4_S (409.9): C, 52.75; H, 4.92; N, 10.25; Found: C, 52.93; H, 5.01; N, 10.41. LC/MS (ESI): 411.21 [M + H]^+^.

#### 3.3.15. 2-(5-(4-Bromobenzylidene)-2,4-dioxothiazolidine-3-yl)*-N-*(2-morpholinoethyl)acetamide (**4o**)

The product was obtained as a white powder from ethanol in 95% yield, mp: 224–226 °C. IR (KBr, cm^−1^): 3298 (NH); 2931 (CH-aliphatic); 1751, 1693, 1664 (CO); ^1^H-NMR (DMSO-*d*_6_, δ ppm): 2.34 (6H, s, CH_2_NCH_2_ and CH_2_CH_2_N); 3.18 (2H, s, NHCH_2_CH_2_); 3.54 (4H, s, CH_2_OCH_2_); 4.25 (2H, s, NCH_2_CO); 7.56 (2H, s, H_3′_ & H_5′_); 7.73 (2H, s, H_2′_ & H_6′_); 7.91 (1H, s, CH=C); 8.21 (1H, s, NH); ^13^C-NMR (DMSO-*d*_6_, δ ppm): 36.6 (NHCH_2_); 43.9 (CH_2_CO); 53.7 (CH_2_NCH_2_); 57.6 (CH_2_CH_2_N); 66.6 (CH_2_OCH_2_); 122.5, 124.7, 132.4, 132.5, 132.9; 165.3, 165.6, 167.3 (CO). Anal. Calc for C_18_H_20_BrN_3_O_4_S (454.3): C, 47.59; H, 4.44; N, 9.25; Found: C, 47.77; H, 4.52; N, 9.49. LC/MS (ESI): 454.3 [M + H]^+^.

#### 3.3.16. 2-(5-(4-Methylbenzylidene)-2,4-dioxothiazolidine-3-yl)*-N-*(2-morpholinoethyl)acetamide (**4p**)

The product was obtained as a white powder from ethanol in 95% yield, mp: 212–214 °C. IR (KBr, cm^−1^): 3291 (NH); 2933 (CH-aliphatic); 1747, 1696, 1657 (CO); ^1^H-NMR (DMSO-*d*_6_, δ ppm): 2.34 (9H, s, CH_2_NCH_2_, CH_2_CH_2_N & CH_3_); 3.18 (2H, s, NHCH_2_ CH_2_); 3.54 (4H, s, CH_2_OCH_2_); 4.24 (2H, s, NCH_2_CO); 7.34 (2H, s, H_3′_ & H_5′_);7.51 (2H, s, H_2′_ & H_6′_); 7.89 (1H, s, CH=C); 8.20 (1H, s, NH); ^13^C-NMR (DMSO-*d*_6_, δ ppm); 21.5 (CH_3_); 36.6 (NHCH_2_); 43.9 (CH_2_CO); 53.7 (CH_2_NCH_2_); 57.6 (CH_2_CH_2_N); 66.6 (CH_2_OCH_2_); 120.4, 130.5, 130.6, 130.7, 133.8 & 141.6; 165.4, 165.8, 167.6 (CO). Anal. Calc for C_19_H_23_N_3_O_4_S (389.5): C, 58.59; H, 5.95; N, 10.79. Found: 58.71; H, 6.09; N, 10.98. LC/MS (ESI): 390.12[M + H]^+^.

#### 3.3.17. 2-(5-(4-Methoxylbenzylidene)-2,4-dioxothiazolidine-3-yl)*-N-*(2-morpholinoethyl)acetamide (**4q**)

The product was obtained as a white powder from ethanol in 95% yield, mp: 231–233 °C. IR (KBr, cm^−1^): 3290 (NH); 2933 (CH-aliphatic); 1739, 1691 & 1660 (CO); ^1^H-NMR (DMSO-*d*_6_, δ ppm): 2.33 (6H, d, CH_2_-N-CH_2_ & CH_2_CH_2_N-); 3.17 (2H, d, *J =* 4.8 Hz, NHCH_2_CH_2_); 3.53 (4H, s, CH_2_OCH_2_); 3.81 (3H, t, *J* = 1.2 Hz, OCH_3_); 4.24 (2H, s, NCH_2_CO); 7.09 (1H, s, H_3′_ & H_5′_); 7.58 (2H, s, H_2′_ & H_6′_); 7.87 (1H, s, CH=CS); 8.20 (1H, s, NH); ^13^C-NMR (DMSO-*d*_6_, δ ppm): 36.6 (NH CH_2_); 43.8 (CH_2_CO); 53.7 (CH_2_NCH_2_; 56.0 (OCH_3_); 57.6 (CH_2_CH_2_N); 66.6 (CH_2_OCH_2_; 115.4, 118.3, 125.8, 132.7, 133.7; 161.7, 165.5, 165.9 & 167.6 (CO). Anal. Calc for C_19_H_23_N_3_O_5_S (405.47): C, 56.28; H, 5.72; N, 10.36; Found: C, 56.44; H, 5.91; N, 10.55. LC/MS (ESI): 406.81[M + H]^+^.

#### 3.3.18. 2-(5-(4-Chlorobenzylidene)-2,4-dioxothiazolidine-3-yl)*-N-*(2-morpholinoethyl)acetamide (**4r**)

The product was obtained as a white powder from ethanol in 94% yield, mp: 222–224 °C. IR (KBr, cm^−1^): 3294 (NH); 2930 (CH-aliphatic); 1751, 1693, 1662 (CO). ^1^H-NMR (DMSO-*d*_6_, δ ppm): 2.33 (6H, s, CH_2_NCH_2_ & CH_2_CH_2_N); 3.17 (2H, s, NHCH_2_ CH_2_); 3.53 (4H, s, CH_2_OCH_2_); 4.25 (2H, s, NCH_2_CO); 7.59 (2H, d, *J* = 1.2 Hz, H_3′_ & H_5′_); 7.63 (2H, s, H_2′_ & H_6′_); 7.92 (1H, s, CH=C); 8.21 (1H, s, NH); ^13^C-NMR (DMSO-*d*_6_, δ ppm): 36.59 (NH CH_2_); 43.9 (CH_2_CO); 53.7 (CH_2_N-CH_2_); 57.6 (NHCH_2_CH_2_); 66.5 (CH_2_OCH_2_); 122.4, 129.9, 132.2, 132.4, 135.8; 165.3; 165.6, 167.3 (CO). Anal. Calc for C_18_H_20_ClN_3_O_4_S (409.9): C, 52.75; H, 4.92; N, 10.25; Found: C, 52.99; H, 5.10; N, 10.47. LC/MS (ESI): 411.51 [M + H]^+^.

#### 3.3.19. 2-(5-(3-Methoxybenzylidene)-2,4-dioxothiazolidine-3-yl)*-N-*(2-morpholinoethyl)acetamide (**4s**)

The product was obtained as a white powder from ethanol in 95% yield, mp: 164–166 °C. IR (KBr, cm^−1^): 3296 (NH); 2943 (CH-aliphatic); 1748, 1693, 1659 (CO); ^1^H-NMR (DMSO-*d*_6_, δ ppm): 2.33 (6H, d, *J* = 6 Hz, (CH_2_NCH_2_ & CH_2_CH_2_N); 3.18 (2H, s, NHCH_2_ CH_2_); 3.53 (4H, s, CH_2_OCH_2_); 3.79 (3H, s, OCH_3_); 4.25 (2H, s, NCH_2_CO); 7.06 (1H, s, H_4′_); 7.18 (2H, s, H_2′_ & H_6′_); 7.45 (1H, d, *J* = 6.6 Hz, H_5′_); 7.91 (1H, s, CH=C); 8.21 (1H, s, NH); ^13^C-NMR (DMSO-*d*_6_, δ ppm): 36.6 (NHCH_2_); 43.9 (CH_2_CO); 53.7 (CH_2_NCH_2_); 55.8 (OCH_3_); 57.6 (CH_2_CH_2_N); 66.6 (CH_2_OCH_2_); 115.9, 117.1, 122.0 122.4, 131.0, 133.7, 134.7 & 160.1; 165. 4, 165.7, 167.5 (CO). Anal. Calc for C_19_H_23_N_3_O_5_S (405.47): C, 56.28; H, 5.72; N, 10.36; Found: C, 56.45; H, 5.91; N, 10.53. LC/MS (ESI): 406.12 [M + H]^+^.

### 3.4. General Procedure for the Synthesis of Amino Acid Ester Derivatives ***5a**–**o***

Compounds **5a–o** were prepared using the method mentioned above for the preparation of **4a**–**s,** employing OxymaPure/DIC as a coupling reagent in the presence of diisopropylamine (DIEA) as a base to neutralize the amino acid ester hydrochloride salt.

#### 3.4.1. 2-(5-Benzylidene-2,4-dioxothiazolidine-3-yl) acetyl)glycinate (**5a**)

The product was obtained as a white powder from ethanol in 97% yield, mp: 177–179 °C. IR (KBr, cm^−1^): 3312 (NH); 1745, 1662, 1698, 1609 (CO); ^1^H-NMR (DMSO-*d*_6_, δ ppm): 3.61 (3H, s, CH3); 3.87 (2H, s, NHCH_2_CO); 4.31 (2H, s, CH_2_CONH); 7.47–7.61 (5H, m, aromatic); 7.93 (1H, s, CH=C); 8.74 (1H, s, NH); ^13^C-NMR (DMSO-*d*_6_, δ ppm): 41.1 (NHCH_2_COO); 43.7 (NCH_2_CO); 52.2 (COOCH_3_); 121.5, 129.8, 130.6, 131.2, 133.3, 133.8; 165.6, 166.1, 167.5, 170.4 (CO). Anal. Calc for C_15_H_14_N_2_O_5_S (334.35): C, 53.89; H, 4.22; N, 8.38; Found: C, 53.11; H, 4.40; N, 8.54. LC/MS (ESI): 335.67 [M + H]^+^.

#### 3.4.2. Methyl-(2-(5-(4-chlorobenzylidene)-2,4-dioxothiazolidine-3-yl)acetyl)glycinate (**5b**)

The product was obtained as a white powder from ethanol in 96% yield, mp: 236–238 °C. IR (KBr, cm^−1^): 3294 (NH); 1751, 1694, 1663, 1610 (CO). ^1^H-NMR (DMSO-*d*_6_, δ ppm): 3.61 (3H, s, OCH_3_); 3.87 (2H, s, NHCH_2_COO); 4.31 (2H, s, NCH_2_CONH); 7.58 (2H, s, H_2′_ & H_6′_); 7.63 (2H, s, H_3′_ & H_5′_); 7.93 (1H, s, CH=CS); 8.74 (1H, s, NH); ^13^C-NMR (DMSO-*d*_6_, δ ppm): 41.1 (NHCH_2_COO); 43.7 (CH_2_CONH); 52.2 (COOCH_3_); 122.3, 129.9, 132.2, 132.5, 135.8; 165.5, 166.1, 167.2, 170.3 (CO). Anal. Calc for C_15_H_13_Cl N_2_O_5_S (368.79): C, 48.85; H, 3.55; N, 7.60; Found: C, 48.01; H, 3.56; N, 7.82. LC/MS (ESI): 370.12 [M + H]^+^.

#### 3.4.3. Methyl-(2-(5-(4-bromobenzylidene)-2,4-dioxothiazolidine-3-yl) acetyl)glycinate (**5c**)

The product was obtained as a white powder from ethanol in 96% yield, mp: 239–241 °C. IR (KBr, cm^−1^): 3294 (NH); 1751, 1693, 1664, 1607 (CO); ^1^H-NMR (DMSO-*d*_6_, δ ppm): 3.60 (3H, s, OCH_3_); 3.87 (2H, s, NHCH_2_COO); 4.31 (2H, s, CH_2_CONH); 7.56 (2H, s, H_2′_ & H_5′_); 7.72 (2H, s, H_3′_ & H_5′_); 7.91 (1H, s, CH=CS); 8.74 (1H, s, NH). ^13^C-NMR (DMSO-*d*_6_, δ ppm): 41.2 (NHCH_2_COO); 43.7 (CH_2_CONH); 52.2 (COOCH_3_); 122.4, 124.7, 132.4, 132.5, 132.6, 132.8; 165.5, 166.1, 167.2, 170.3 (CO). Anal. Calc for C_15_H_13_BrN_2_O_5_S (413.24): C, 43.60; H, 3.17; N, 6.78. Found: C, 43.81; H, 3.31; N, 6.91. LC/MS (ESI): 415.41 [M + H]^+^.

#### 3.4.4. Methyl-4-(2-(5-benzylidene-2,4-dioxothiazolidine-3-yl)acetamido)butanoate (**5d**)

The product was obtained as a white powder from ethanol in 98% yield, mp: 169–171 °C. IR (KBr, cm^−1^): 3306 (NH); 1734, 1692, 1660 & 1607 (CO); ^1^H-NMR (DMSO-*d*_6_, δ ppm): 1.62 (2H, m, CH_2_CH_2_CH_2_); 2.28 (2H, s, CH_2_CH_2_CO); 3.05 (2H, s, NHCH_2_); 3.55 (3H, s, OCH_3_); 4.22 (2H, s, NCH_2_CO); 7.48–7.49 (3H, m, H_3′_, H_4′_ & H_5′_); 7.61 (2H, s, H_2′_ & H_6′_);7.93 (1H, s, CH=CS); 8.26 (1H, s, NH); ^13^C-NMR (DMSO-*d*_6_, δ ppm): 24.8 (CH_2_CH_2_CH_2_); 31.0 (CH_2_CH_2_CO); 38.5 (NHCH_2_CH_2_); 43.9 (NCH_2_CO); 51.7 (COOCH_3_); 121.6, 129.8, 130.6, 131.2, 133.3, 133.7; 165.4, 165.7, 167.5, 173.5 (CO). Anal. Calc for C_17_H_18_N_2_O_5_S (362.40): C, 56.34; H, 5.01; N, 7.73; Found: C, 56.56; H, 5.19; N, 7.98. LC/MS (ESI): 363.41 [M + H]^+^.

#### 3.4.5. Methyl-4-(2-(5-(4-chlorobenzylidene)-2,4-dioxothiazolidine-3-yl)acetamide)butanoate (**5e**)

The product was obtained as a white powder from ethanol in 97% yield, mp: 192–194 °C. IR (KBr, cm^−1^): 3304 (NH); 1743, 1692, 1662, 1609 (CO); ^1^H-NMR (DMSO-*d*_6_, δ ppm): 1.62 (2H, s, CH_2_CH_2_CH_2_); 2.28 (2H, s, CH_2_CH_2_CO); 3.05 (2H, s, NHCH_2_CH_2_); 3.56 (3H, s, OCH_3_); 4.22 (2H, s, NCH_2_CO); 7.59 (2H, s, H_3′_ & H_5′_); 7.64 (2H, s, H_2′_ & H_6′_); 7.93 (1H, s, CH=CS); 8.25 (1H, s, NH); ^13^C-NMR (DMSO-*d*_6_, δ ppm): δ 24.8 (CH_2_CH_2_CH_2_); 31.0 (CH_2_CH_2_CO); 38.5 (NHCH_2_CO); 44.0 (NCH_2_CO); 51.7 (COOCH_3_); 122.4, 129.9, 132.2, 132.4, 135.8; 165.3, 165.6, 167.3, 173.5 (CO). Anal. Calc for C_17_H_17_ClN_2_O_5_S (396.84): C, 51.45; H, 4.32; N, 7.06; Found: C, 51.61; H, 4.44; N, 7.28. LC/MS (ESI): 398.12 [M + H]^+^.

#### 3.4.6. Methyl-4-(2-(5-(4-bromobenzylidene)-2,4-dioxothiazolidine-3-yl)acetamido)butanoate (**5f**)

The product was obtained as a white powder from ethanol in 95% yield, mp: 191–193 °C. IR (KBr, cm^−1^): 3293 (NH); 1744, 1690, 1659, 1608 (CO); ^1^H-NMR (DMSO-*d*_6_, δ ppm): 1.63 (2H, s, CH_2_CH_2_CH_2_); 2.28 (2H, s, CH_2_CH_2_CO); 3.06 (2H, s, NHCH_2_CH_2_); 3.55 (3H, s, OCH_3_); 4.22 (2H, s, NCH_2_CO); 7.54 (2H, d, *J* = 2.4 Hz, H_2′_ & H_6′_); 7.70 (2H, s, H_3′_ & H_5′_); 7.89 (1H, s, CH=CS); 8.27 (1H, s, NH); ^13^C-NMR (DMSO-*d*_6_, δ ppm): 24.8 (CH_2_CH_2_CH_2_); 31.0 (CH_2_CH_2_CO); 38.5 (NHCH_2_CO); 43.9 (NCH_2_CO); 51.7 (COOCH_3_); 122.4, 124.7, 132.3, 132.5, 132.8; 165.3, 165.6, 167.3, 173.4 (CO). Anal. Calc for C_17_H_17_BrN_2_O_5_S (441.30): C, 46.27; H, 3.88; N, 6.35. Found: C, 46.44; H, 4.05; N, 6.58. LC/MS (ESI): 442.52 [M + H]^+^.

#### 3.4.7. Methyl-2-(5-benzylidene-2,4-dioxothiazolidine-3-yl)acetyl)valinate (**5g**)

The product was obtained as a white powder from ethanol in 98% yield, mp: 186–188 °C. IR (KBr, cm^−1^): 3300 (NH); 1749, 1697, 1662 (CO); ^1^H-NMR (DMSO-*d*_6_, δ ppm): δ 0.84 (6H, s, 2 CH_3_); 2.00 (1H, s, CH(CH_3_)_2_); 3.61 (3H, s, OCH_3_); 4.18 (1H, s, NHCHCO); 4.34 (2H, s, NCH_2_CO); 7.51–7.60 (5H, m, -Ph proton); 7.92 (1H, s, CH=CS); 8.64 (1H, s, NH); ^13^C-NMR (DMSO-*d*_6_, δ ppm): δ 18.5, 19.3 (2CH_3_); 30.6 (CH (CH_3_)_2_); 43.6 (NCH_2_CO); 52.2 (COOCH_3_); 58.0 (CH CO); 121.4, 129.8,130.6, 131.2, 133.3, 133.8; 165.6, 165.8, 167.4, 172.1 (CO). Anal. Calc for C_18_H_20_N_2_O_5_S (376.43): C, 57.43; H, 5.36; N, 7.44; Found: C, 57.66; H, 5.51; N, 7.61. LC/MS (ESI): 377.92 [M + H]^+^.

#### 3.4.8. Methyl-(2-(5-(4-chlorobenzylidene)-2,4-dioxothiazolidine-3-yl)acetyl)valinate (**5h**)

The product was obtained as a yellowish white powder from ethanol in 97% yield, mp: 193–195 °C. IR (KBr, cm^−1^): 3278 (NH), 1745, 1688, 1658, 1606 (CO); ^1^H-NMR (DMSO-*d*_6_, δ ppm): 0.84 (6H, s, 2CH_3_); 2.00 (1H, s, CH(CH_3_)_2_); 3.62 (3H, s, OCH_3_); 4.19 (1H, s, NHCHCO); 4.35 (2H, s, NCH_2_CO); 7.55 (2H, d, *J* = 9.6 Hz, H_2′_ & H_6′_); 7.61 (2H, s, H_3′_ & H_5′_); 7.91 (1H, s, CH=CS); 8.65 (1H, s, NH); ^13^C-NMR (DMSO-*d*_6_, δ ppm): 18.5, 19.3 (2CH_3_); 30.6 (CH (CH_3_)_2_); 43.6 (NCH_2_CO); 52.2 (COOCH_3_); 57.9 (CH CO); 121.2, 129.9, 132.2, 132.5, 135.8; 165.5, 165.8, 167.1, 172.1 (CO). Anal. Calc for C_18_H_19_ClN_2_O_5_S (410.87): C, 52.62; H, 4.66; N, 6.82; Found: C, 52.81; H, 4.72; N, 6.63. LC/MS (ESI): 412.33 [M + H]^+^.

#### 3.4.9. Methyl-2-(5-(4-bromobenzylidene)-2,4-dioxothiazolidine-3-yl)acetyl)valinate (**5i**)

The product was obtained as a yellowish white powder from ethylacetate-ethanol (2:1) in 96% yield, mp: 222–224 °C. IR (KBr, cm^−1^): 3280 (NH), 1744, 1688, 1658, 1606 (CO); ^1^H-NMR (DMSO-*d*_6_, δ ppm): 0.84 (6H, d, 2CH_3_); 2.01 (1H, d, CH(CH_3_)_2_); 3.62 (3H, s, OCH_3_); 4.19 (1H, s, CHCO); 4.35 (2H, s, NCH_2_CO); 7.53 (2H, s, H_2′_ & H_6′_); 7.70 (2H, s, H_3′_ & H_5′_); 7.89 (1H, s, CH=CS); 8.65 (1H, s, NH); ^13^C-NMR (DMSO-*d*_6_, δ ppm): 18.5, 19.3 (2CH_3_); 30.6 (CH (CH_3_)_2_); 43.6 (-NCH_2_CO); 52.2 (COOCH_3_); 57.9 (CHCO); 122.3, 124.7, 132.3, 132.5, 132.6, 132.8; 165.5, 165.8, 167.1, 172.2 (CO). Anal. Calc for C_18_H_19_BrN_2_O_5_S (455.32): C, 47.48; H, 4.21; N, 6.15; Found: C, 47.67; H, 4.39; N, 6.31. LC/MS (ESI): 456.43 [M + H]^+^.

#### 3.4.10. Methyl-2-(5-benzylidene-2,4-dioxothiazolidine-3-yl)acetyl)alaninate (**5j**)

The product was obtained as a white powder from ethanol and 2 drops dimethylformamide in 97% yield, mp: 202–204 °C. IR (KBr, cm^−1^): 3304 (NH); 1743, 1691, 1663, 1607 (CO); ^1^H-NMR (DMSO-*d*_6_, δ ppm): 1.26 (3H, s, CH_3_); 3.59 (3H, s, OCH_3_); 4.27, 4.28 (3H, d, NCH_2_COCHCOO); 7.47–7.61 (5H, m, -Ph proton); 7.93 (1H, s, CH=CS); 8.74 (1H, s, NH); ^13^C-NMR (DMSO-*d*_6_, δ ppm): δ 17.5 (CH_3_); 43.6 (NCH_2_CO); 48.2 (NHCHCO); 52.4 (COOCH_3_); 121.5, 129.8, 130.6, 131.2, 133.3, 133.8; 165.4, 165.6, 167.4, 173.1 (CO). Anal. Calc for C_16_H_16_N_2_O_5_S (348.37): C, 55.16; H, 4.63; N, 8.04; Found: C, 55.33; H, 4.78; N, 8.24. LC/MS (ESI): 349.56 [M + H]^+^.

#### 3.4.11. Methyl-2-(5-(4-chlorobenzylidene)-2,4-dioxothiazolidine-3-yl)acetyl)alaninate (**5k**)

The product was obtained as a yellowish white powder from ethylacetate-ethanol (2:1) in 95% yield, mp: 233–235 °C. IR (KBr, cm^−1^): 3295 (NH); 1748, 1693, 1660, 1608 (CO); ^1^H-NMR (DMSO-*d*_6_, δ ppm): δ 1.25 (3H, s, CH_3_); 3.59 (3H, s, OCH_3_); 4.27, 4.28 (3H, d, NCH_2_COCHCOO); 7.58 (2H, s, H_2′_ & H_6′_); 7.62 (2H, s, H_3′_ & H_5′_); 7.92 (1H, s, CH=CS); 8.74 (1H, s, NH); ^13^C-NMR DMSO-*d*_6_, δ ppm): δ 17.5 (CH_3_); 43.6 (NCH_2_CO); 48.2 (NHCHCOO); 52.4 (COOCH_3_); 122.2, 129.9, 132.2, 132.5, 135.8 (C-sp^2^); 165.3, 165.5, 167.2, 173.1 (CO). Anal. Calc for C_16_H_15_ClN_2_O_5_S (382.82): C, 50.20; H, 3.95; N, 7.32; Found: C, 50.41; H, 4.12; N, 7.51. LC/MS (ESI): 384.22[M + H]^+^.

#### 3.4.12. Methyl-2-(5-(4-bromobenzylidene)-2,4-dioxothiazolidine-3-yl) acetyl)alaninate (**5l**)

The product was obtained as a white powder from ethylacetate-ethanol (2:1) in 94% yield, mp: 220–222 °C. IR (KBr, cm^−1^): 3300 (NH); 1745, 1692, 1661, 1606 (CO); ^1^H-NMR (DMSO-*d*_6_, δ ppm): 1.26 (3H, s, CH_3_); 3.60 (3H, s, OCH_3_); 4.28 (3H, s, NCH_2_COCHCOO); 7.55 (2H, s, H_2′_ & H_6′_); 7.71 (2H, s, H_3′_ & H_5′_); 7.90 (1H, s, CH=CS); 8.74 (1H, s, NH); ^13^C-NMR (DMSO-*d*_6_, δ ppm): δ 17.5 (CH_3_); 43.6 (-NCH_2_CO); 48.2 (NHCH-CO); 52.4 (COOCH_3_); 122.3, 124.7, 132.4, 132.5, 132.6, 132.8 (C-sp^2^); 165.3, 165.5, 167.1, 173.1 (CO). Anal. Calc for C_16_H_15_BrN_2_O_5_S (427.27): C, 44.98; H, 3.54; N, 6.56. Found: C, 45.12; H, 3.66; N, 6.73. LC/MS (ESI): 42,854 [M + H]^+^.

#### 3.4.13. Methyl-(2-(5-benzylidene-2,4-dioxothiazolidine-3-yl) acetyl)phenylalaninate (**5m**)

The product was obtained as a white powder from ethanol in 96% yield, mp: 164–166 °C. IR (KBr, cm^−1^): 3329 (NH); 1740, 1693, 1662 & 1609 (CO); ^1^H-NMR (DMSO-*d*_6_, δ ppm): 2.92 (2H, t, CH_2_ph); 3.31 (3H, s, COOCH_3_); 4.26 (2H, s, NCH_2_CONH); 4.32 (1H, s, NHCHCOO), 7.19–7.61 (10H, m, 2 -Ph proton); 7.93 (1H, s, CH=CS); 8.68 (1H, s, NH); ^13^C-NMR (DMSO-*d*_6_, δ ppm): δ 37.3 (CH_2_ph); 43.6 (CH_2_CONH); 54.9 (COOCH_3_); 81.4 (CHCOOCH_3_), 121.5, 127.0, 128.6, 129.7, 129.8, 130.6, 131.2, 133.3, 133.8, 137.3; 165.4, 165.6, 167.4, 170.5 (CO). Anal. Calc for C_22_H_20_N_2_O_5_S (424.47): C, 62.25; H, 4.75; N, 6.60; Found: C, 62.41; H, 4.87; N, 6.80. LC/MS (ESI): 425.82 [M + H]^+^.

#### 3.4.14. Methyl-2-(5-(4-chlorobenzylidene)-2,4-dioxothiazolidine-3yl)acetyl)phenylalaninate (**5n**)

The product was obtained as a white powder from ethanol and 2 drops dimethylformamide 94% yield, mp: 164–166 °C. IR (KBr, cm^−1^): 3341 (NH); 1741, 1685, 1607 (CO); ^1^H-NMR (DMSO-*d*_6_, δ ppm): 2.91 (2H, t, -CH_2_ph); 3.32 (3H, s, COOCH_3_); 4.26 (2H, s, CH_2_CONH), 4.32 (1H, d, *J* = 4.2 Hz, NHCHCOOCH_3_), 7.19–7.25 (5H, m, -Ph proton); 7.58 (2H, s, H_2′_ & H_6′_); 7.63 (2H, s, H_3′_ & H_5′_); 7.93 (1H, s, CH=CS); 8.68 (1H, s, NH); ^13^C-NMR (DMSO-*d*_6_, δ ppm): 37.3 (CH_2_ph); 43.7 (CH_2_CONH); 54.9 (COOCH_3_); 81.4 (CHCOOCH_3_); 122.3, 127.0, 128.6, 129.7, 129.9, 132.2, 132.5, 135.8, 137.3; 165.4, 165.5, 167.1, 170.5 (CO). Anal. Calc for C_22_H_19_ClN_2_O_5_S (458.91): C, 57.58; H, 4.17; N, 6.10; Found: C, 57.77; H, 4.32; N, 6.29. LC/MS (ESI): 410.10 [M + H]^+^.

#### 3.4.15. Methyl-(2-(5-(4-bromobenzylidene)-2,4-dioxothiazolidine-3-yl)acetyl)phenylalaninate (**5o**)

The product was obtained as a white powder from ethanol in 94% yield, mp: 161–163 °C. IR (KBr, cm^−1^): 3341 (NH); 1741, 1683, 1606 (CO); ^1^H-NMR (DMSO-*d*_6_, δ ppm): 2.91 (2 H, t, CH_2_ph); 3.31 (3H, s, COOCH_3_); 4.26 (2H, s, CH_2_CONH); 4.32 (1H, d, *J* = 4.2 Hz, NHCHCOOCH_3_), 7.19–7.25 (5H, m, -Ph proton); 7.55 (2H, s, H_2′_ & H_6′_); 7.71 (2H, s, H_3′_ & H_5′_); 7.91 (1H, s, CH=CS); 8.69 (1H, s, NH). ^13^C-NMR (DMSO-*d*_6_, δ ppm): 37.3 (CH_2_ph); 43.7 (CH_2_CONH); 54.9 (COOCH_3_); 81.3 (CHCOOCH_3_), 122.4, 124.7, 127.0, 128.6, 129.7, 132.2, 132.4, 132.5, 132.6, 132.8, 137.3; 165.4, 165.5, 167.1, 170.5 (CO). Anal. Calc for C_22_H_19_BrN_2_O_5_S (503.37): C, 52.49; H, 3.80; N, 5.57; Found: C, 52.64; H, 4.01; N, 5.71. LC/MS (ESI): 503.54 [M + H]^+^.

### 3.5. Antimicrobial Activity

#### 3.5.1. Microbial Preparation

All tested organisms were pre-cultured on Nutrient agar plates (Oxoid, Lenexa, KS, USA) incubated at 37 °C for 18–24 h, and microbial suspensions of each of the pure isolates, following pre culture, were prepared in nutrient broth tubes with 0.5 McFarland turbidity needed for the in vivo antimicrobial test. We tested two gram-positive bacteria, namely *Staphylococcus aureus* ATCC 29213 and *Bacillus subtilis* ATCC 10400, two-gram negative bacteria, namely *Escherichia coli* ATCC 25922 and *Pseudomonas aeruginosa* ATCC 27853, and one fungal isolate, *Candida albicans* ATCC 10231.

#### 3.5.2. Well Diffusion Technique

Before applying the in vivo antimicrobial test, 20 mg of each chemical compound was dissolved in 1 mL DMSO and mixed thoroughly to form a solution. Mueller Hinton plates were prepared for the sensitivity test. Next, each microbial suspension was spread on the surface of the plates using a sterile cotton swab. Equidistant holes with a diameter of 6 mm were then made using a sterile cork borer. One hundred µL of each chemical compound was added to the corresponding well. Plates were incubated at 37 °C for 18–24 h. Antimicrobial activity was determined by measuring the inhibition zone around each well in mm. Inhibition zones above 8 mm in diameter indicated susceptibility of the micro-organism to the specific compound used. Data were compared to the positive control standard impenem 10 μg antibiotic discs, sulfamethoxazole trimethoprim for the bacterial isolates, and fluconazole for the *Candida* isolate. Tests were repeated three times and the average of the inhibition zone was recorded (Table 1).

## 4. Conclusions

Novel thiazolidine-2,4-diones carboxamide and amino acid derivatives were synthesized in excellent yield and purity using OxymaPure/DIC coupling methodology and were characterized by IR, NMR (^1^H and ^13^C), elemental analysis, and LC-MS. The presence of the OxymaPure as additive during the coupling facilitates this reaction, which is not straightforward due to the poor reactivity of the carboxylic moiety.

Interestingly, some compounds from the three series showed weak activity against *E. coli*, while most of the prepared compounds showed weak to moderate activity against gram-negative bacteria *P. aeruginosa* and antifungal activity against *C. albicans.* On the other hand, none of the prepared compounds showed any any antimicrobial activity against Gram-positive bacteria (*S. aureus* and *B. subtilis*) except compound **3g** that gave good activity against *S. aureus*. These results are of special relevance because to the lack of new antibiotic drugs against Gram-negative resistant strains.

Finally, the type of substituent at the thiazolidine ring and at carboxylic moiety (carboxamide or amino acid ester derivatives) has a great impact on the antimicrobial activity of the compound. Based on these results, the preparation of a new series of compounds with different thiazolidine derivatives are currently carrying out in our laboratories with the objective of finding compounds with better antimicrobial activity against Gram-negative bacteria.

## Figures and Tables

**Table 1:** Antimicrobial activity (zones of inhibition, mm) compared with several standard antimicrobial drugs.

Average Inhibition Zone in mm					
Chemical Compounds	*S. aureus*	*Bacillus subtilis*	*E. coli*	*Ps. aeruginosa*	*C. albicans*
**3a**	12	-	11	16	15
**3b**	12	-	12	11	11
**3c**	-	-	-	10	-
**3d**	-	-	10	15	15
**3e**	-	-	12	13	-
**3f**	-	-	-	13	-
**3g**	20	-	7	14	7
**4a**	-	-	-	12	14
**4b**	-	-	-	-	12
**4c**	-	-	-	-	12
**4d**	-	-	-	14	-
**4e**	-	-	-	-	13
**4f**	-	-	-	11	-
**4g**	-	-	-	12	14
**4h**	-	-	-	13	-
**4i**	-	-	-	12	-
**4k**	-	-	-	-	15
**4l**	-	-	-	-	13
**4m**	-	-	-	10	-
**4n**	-	-	-	11	13
**4o**	-	-	-	10	-
**4p**	-	-	-	12	14
**4q**	-	-	-	12	12
**4r**	-	-	-	14	-
**4s**	-	-	11	12	12
**5a**	-	-	-	-	-
**5b**	-	-	-	-	15
**5c**	-	-	-	-	13
**5d**	-	-	-	-	-
**5e**	-	-	-	12	-
**5f**	-	-	-	-	-
**5g**	-	-	10	12	15
**5h**	-	-	11	11	13
**5i**	-	-	12	12	-
**5j**	-	-	-	-	-
**5k**	-	-	7	13	18
**5l**	-	-	-	-	12
**5m**	-	-	-	-	12
**5n**	-	-	10	-	-
**5o**	-	-	11	12	16
Impenem *	30	34	20	35	
SXT **	22	20	-	30	
Fluconazole	-	-	-	-	-

* 10 µg; ** 23.75/1.25 µg.

## Supplementary Materials

The following are available online, Figures S1–S37 represent the NMR (^1^H and ^13^C) spectra for the prepared compounds.
