# Supplementary material for: Synthesis and Antimicrobial Activity of a New Series of Thiazolidine-2,4-diones Carboxamide and Amino Acid Derivatives
Source: Molecules. 2019 Dec 27;25(1):105. doi: 10.3390/molecules25010105 (PMC6983134; doi:10.3390/molecules25010105)

Supporting Information

Synthesis of and Antimicrobial Activity a New Series of Thiazolidine-2, 4-diones Carboxamide and Amino acid Derivatives

Rakia Abd Alhameed, Zainab Almarhoon, Sarah I. Bukhari, Ayman El-Faham,* Beatriz G. de la Torre,[4,5] Fernando Albericio*

Table of Content

**Figure S1**	^{1}H- and ^{13}C-NMR for compound **3b**
**Figure S2**	^{1}H- and ^{13}C-NMR for compound **3c**
**Figure S3**	^{1}H- and ^{13}C-NMR for compound **3e**
**Figure S4**	^{1}H- and ^{13}C-NMR for compound **4a**
**Figure S5**	^{1}H- and ^{13}C-NMR for compound **4b**
**Figure S6**	^{1}H- and ^{13}C-NMR for compound **4c**
**Figure S7**	^{1}H- and ^{13}C-NMR for compound **4d**
**Figure S8**	^{1}H- and ^{13}C-NMR for compound **4e**
**Figure S9**	^{1}H- and ^{13}C-NMR for compound **4f**
**Figure S10**	^{1}H- and ^{13}C-NMR for compound **4g**
**Figure S11**	^{1}H- and ^{13}C-NMR for compound **4h**
**Figure S12**	^{1}H- and ^{13}C-NMR for compound **4i**
**Figure S13**	^{1}H- and ^{13}C-NMR for compound **4j**
**Figure S14**	^{1}H- and ^{13}C-NMR for compound **4k**
**Figure S15**	^{1}H- and ^{13}C-NMR for compound **4l**
**Figure S16**	^{1}H- and ^{13}C-NMR for compound **4m**
**Figure S17**	^{1}H- and ^{13}C-NMR for compound **4n**
**Figure S18**	^{1}H- and ^{13}C-NMR for compound **4o**
**Figure S19**	^{1}H- and ^{13}C-NMR for compound **4p**
**Figure S20**	^{1}H- and ^{13}C-NMR for compound **4q**
**Figure S21**	^{1}H- and ^{13}C-NMR for compound **4r**

Figure S22 ^{1}H- and ^{13}C-NMR for compound **4s**

Figure S23 ^{1}H- and ^{13}C-NMR for compound **5a**

Figure S24 ^{1}H- and ^{13}C-NMR for compound **5b**

Figure S25 ^{1}H- and ^{13}C-NMR for compound **5c**

Figure S26 ^{1}H- and ^{13}C-NMR for compound **5d**

Figure S27 ^{1}H- and ^{13}C-NMR for compound **5e**

Figure S28 ^{1}H- and ^{13}C-NMR for compound **5f**

Figure S29 ^{1}H- and ^{13}C-NMR for compound **5g**

Figure S30 ^{1}H- and ^{13}C-NMR for compound **5h**

Figure S31 ^{1}H- and ^{13}C-NMR for compound **5i**

Figure S32 ^{1}H- and ^{13}C-NMR for compound **5j**

Figure S33 ^{1}H- and ^{13}C-NMR for compound **5k**

Figure S34 ^{1}H- and ^{13}C-NMR for compound **5l**

Figure S35 ^{1}H- and ^{13}C-NMR for compound **5m**

Figure S36 ^{1}H- and ^{13}C-NMR for compound **5n**

Figure S37 ^{1}H- and ^{13}C-NMR for compound **5o**

Figure S1 ^{1}H- and ^{13}C-NMR for compound **3b**

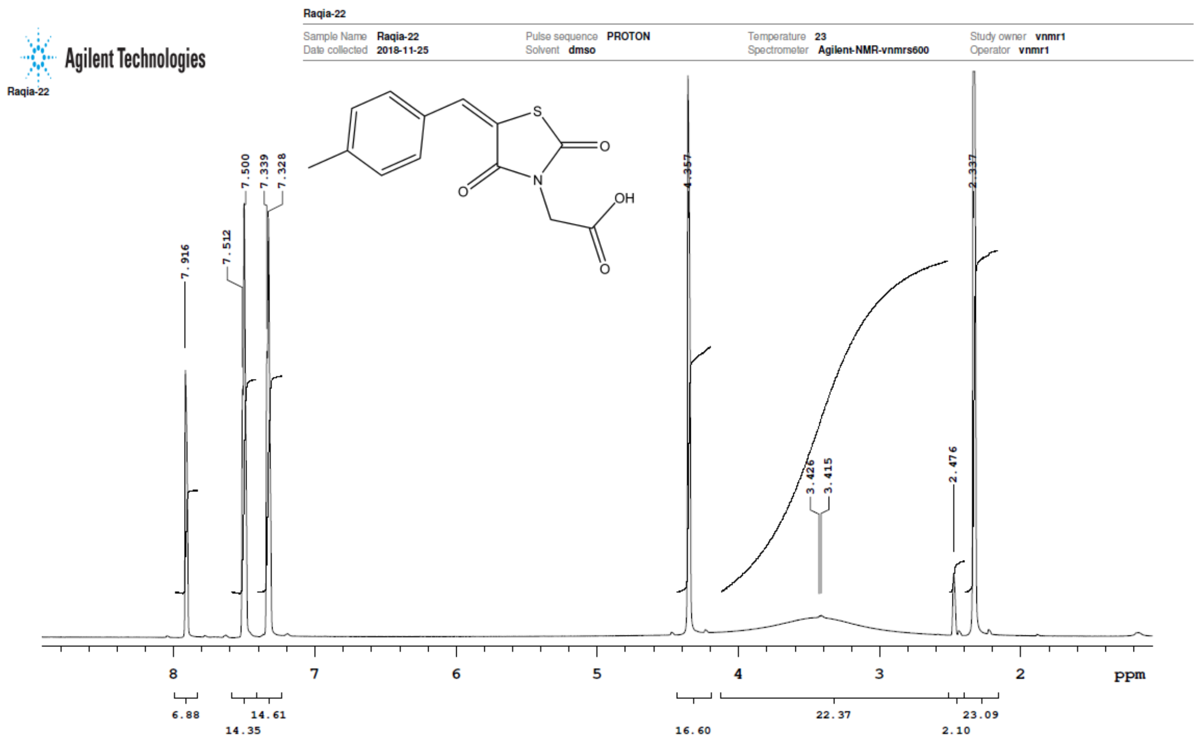

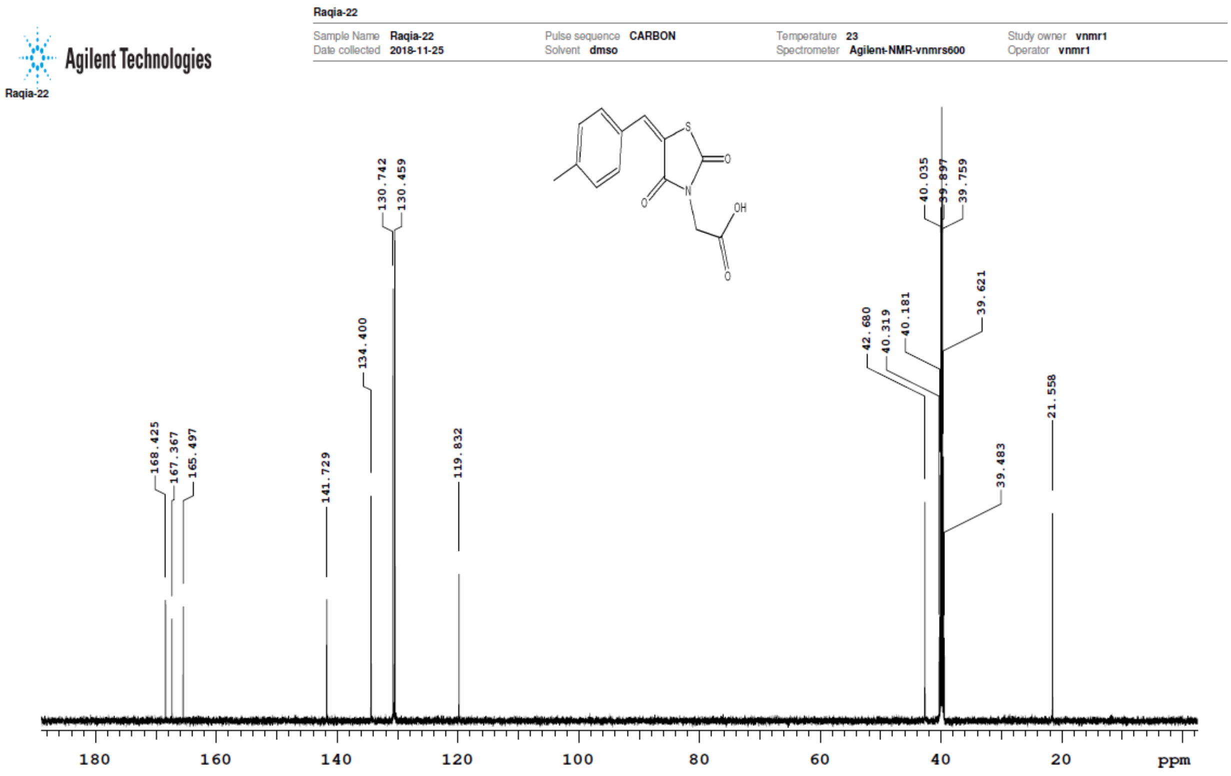

Figure S2 ^{1}H- and ^{13}C-NMR for compound **3c**

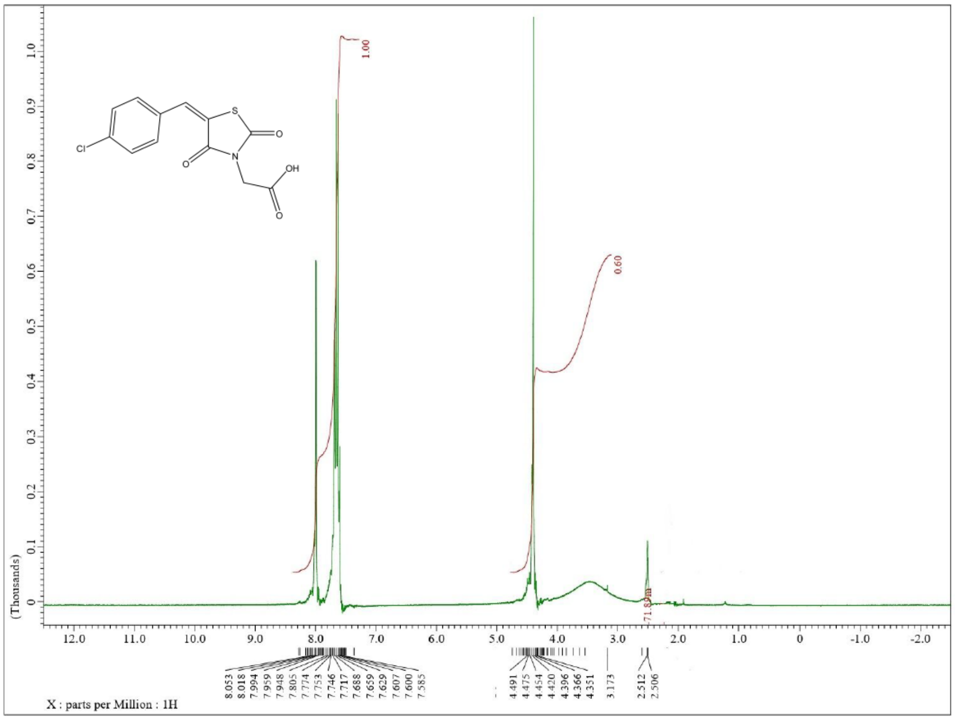

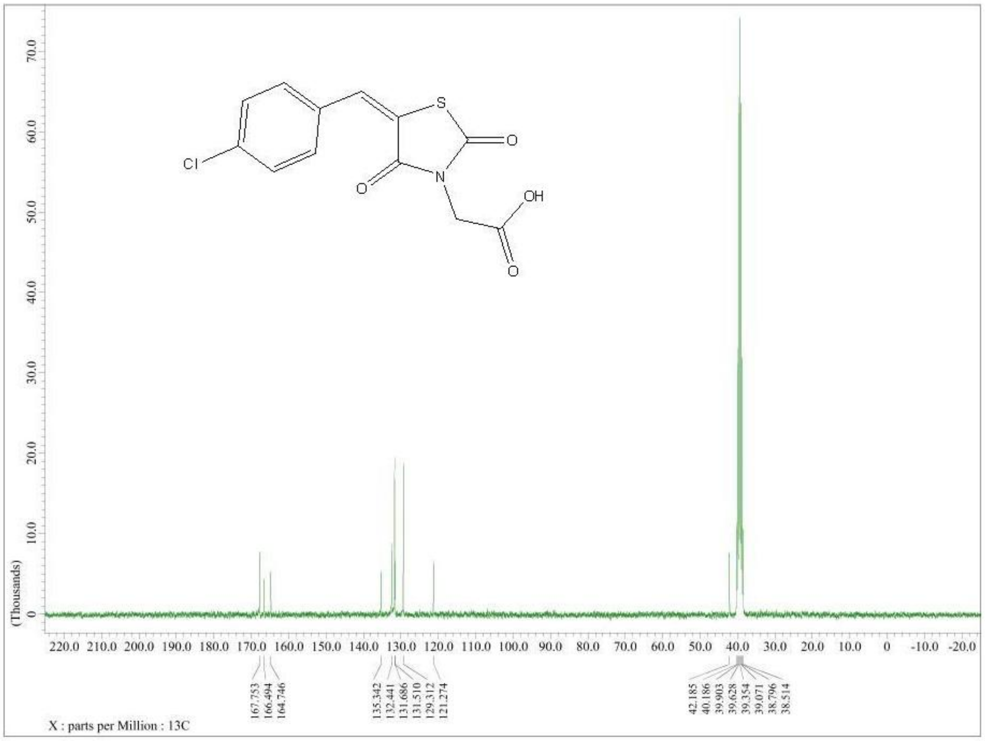

Figure S3 ^{1}H- and ^{13}C-NMR for compound **3e**

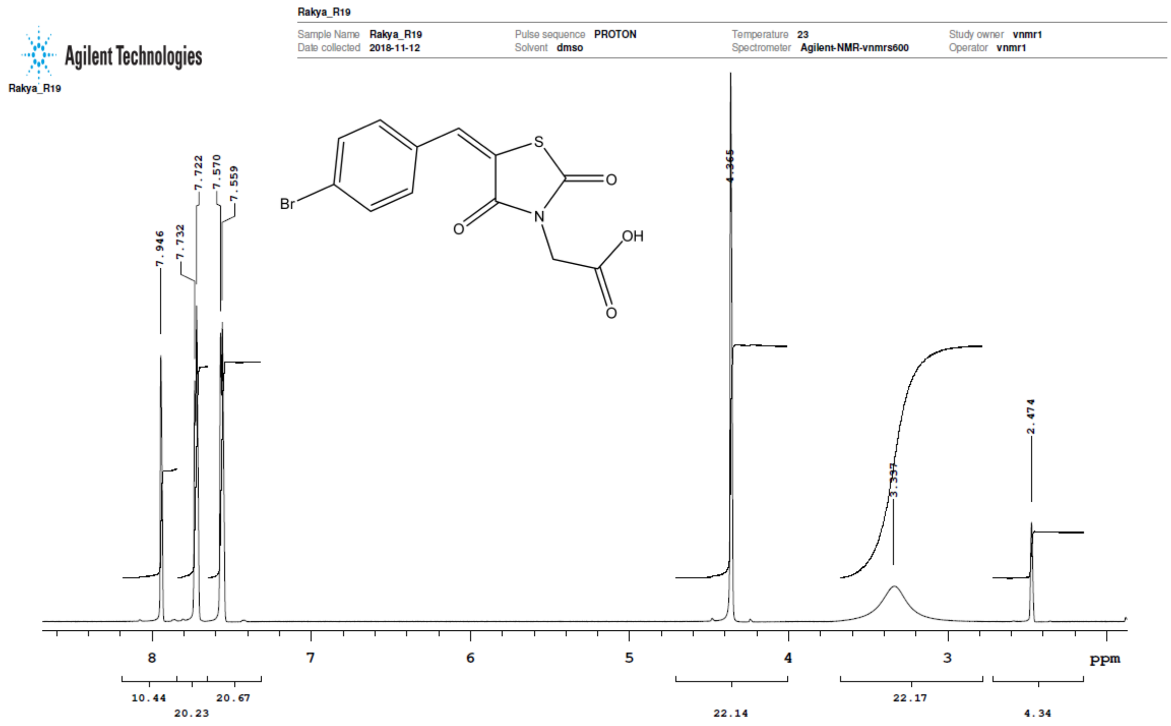

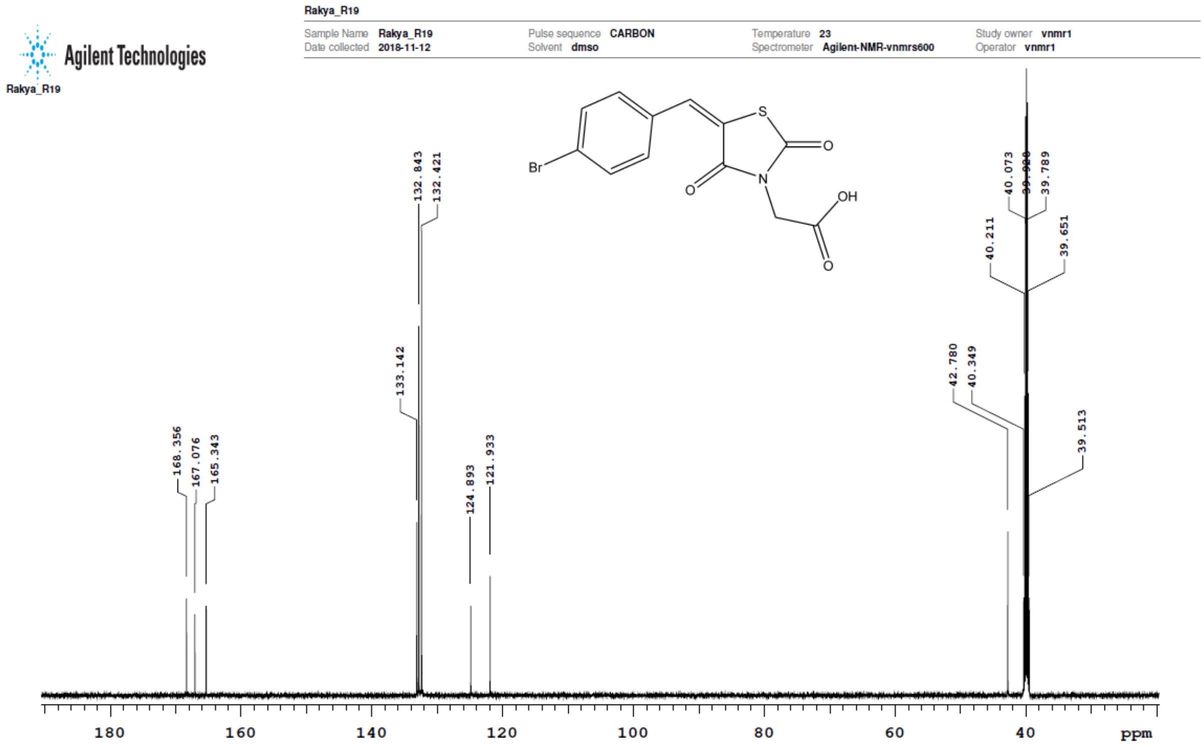

Figure S4 ^{1}H- and ^{13}C-NMR for compound **4a**

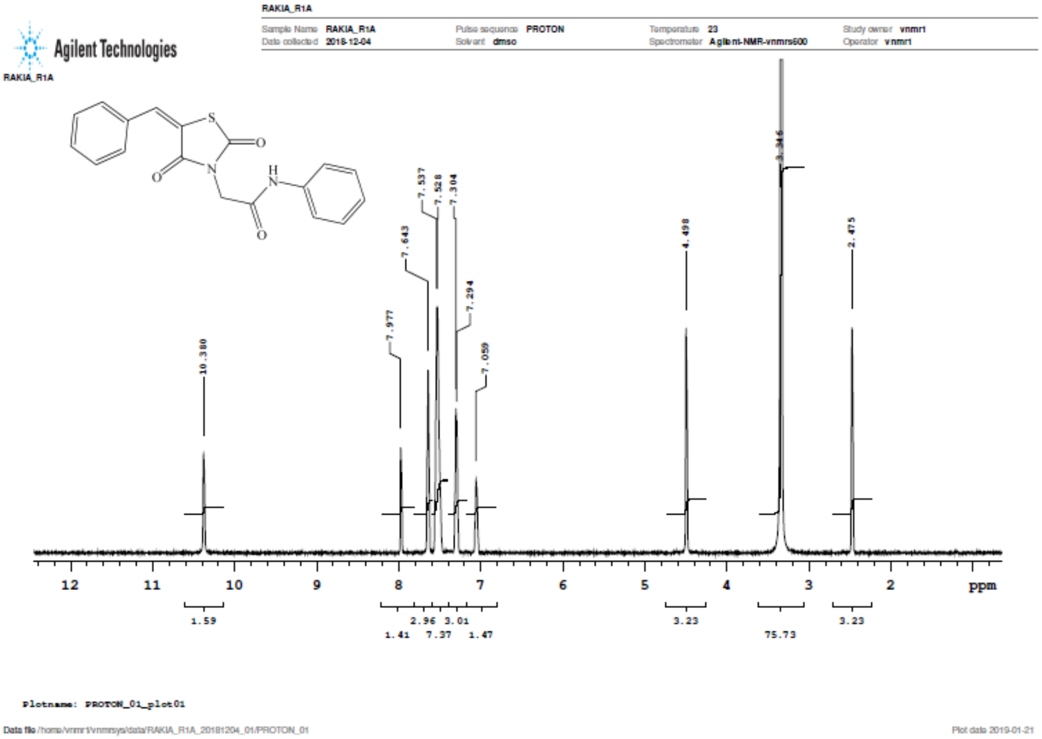

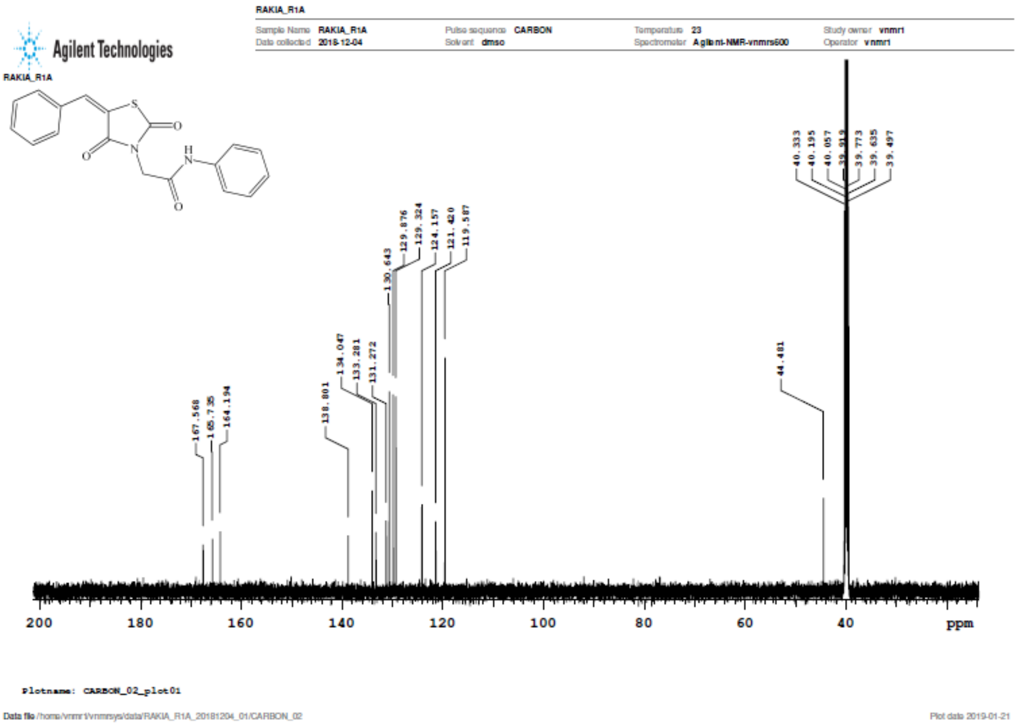

Figure S5 ^{1}H- and ^{13}C-NMR for compound **4b**

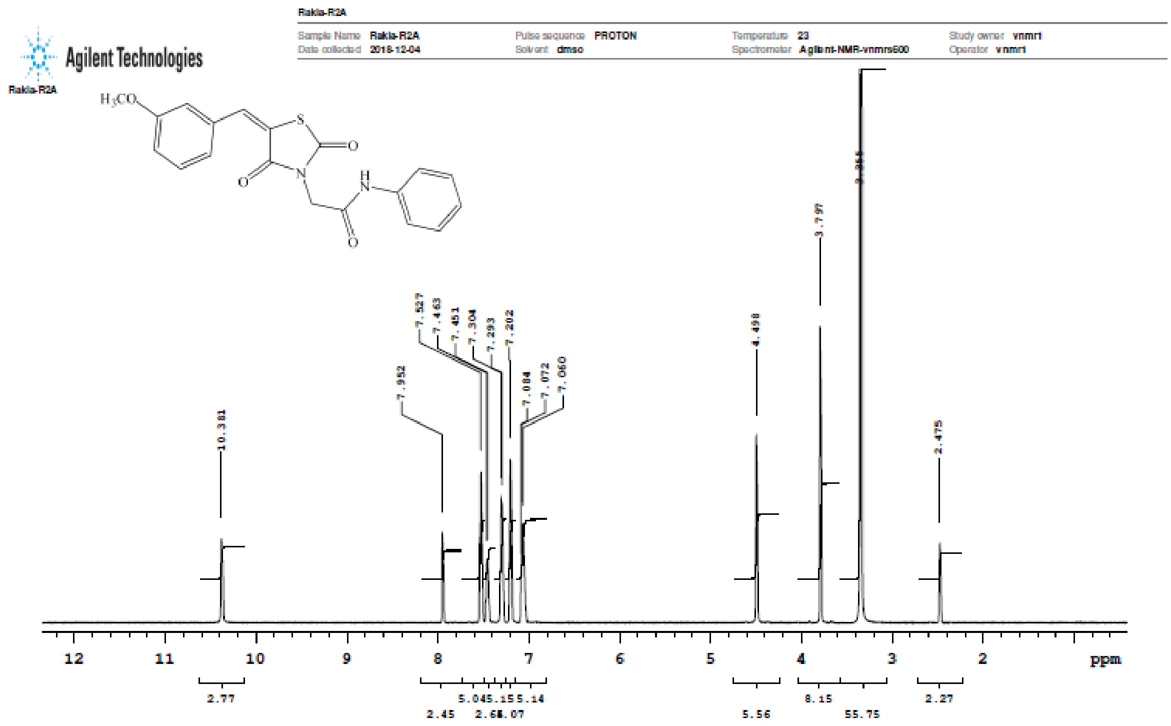

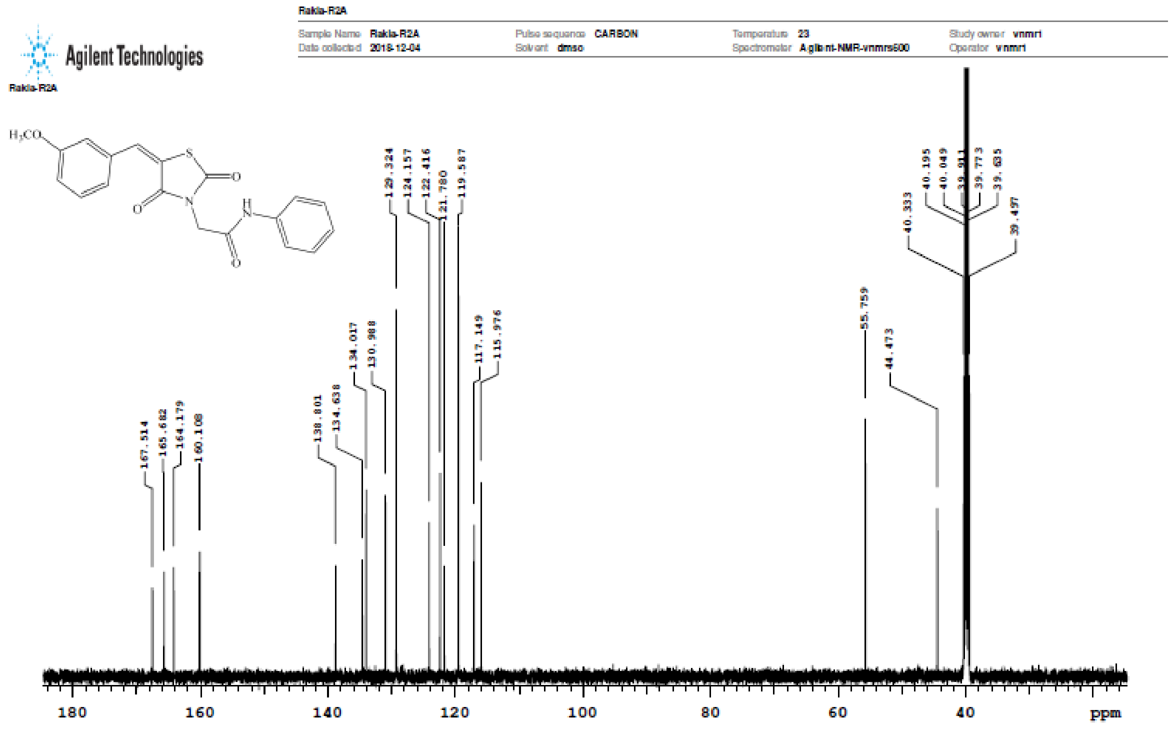

Plotname: CARBON 01 plot01

Figure S6 ^{1}H- and ^{13}C-NMR for compound **4c**

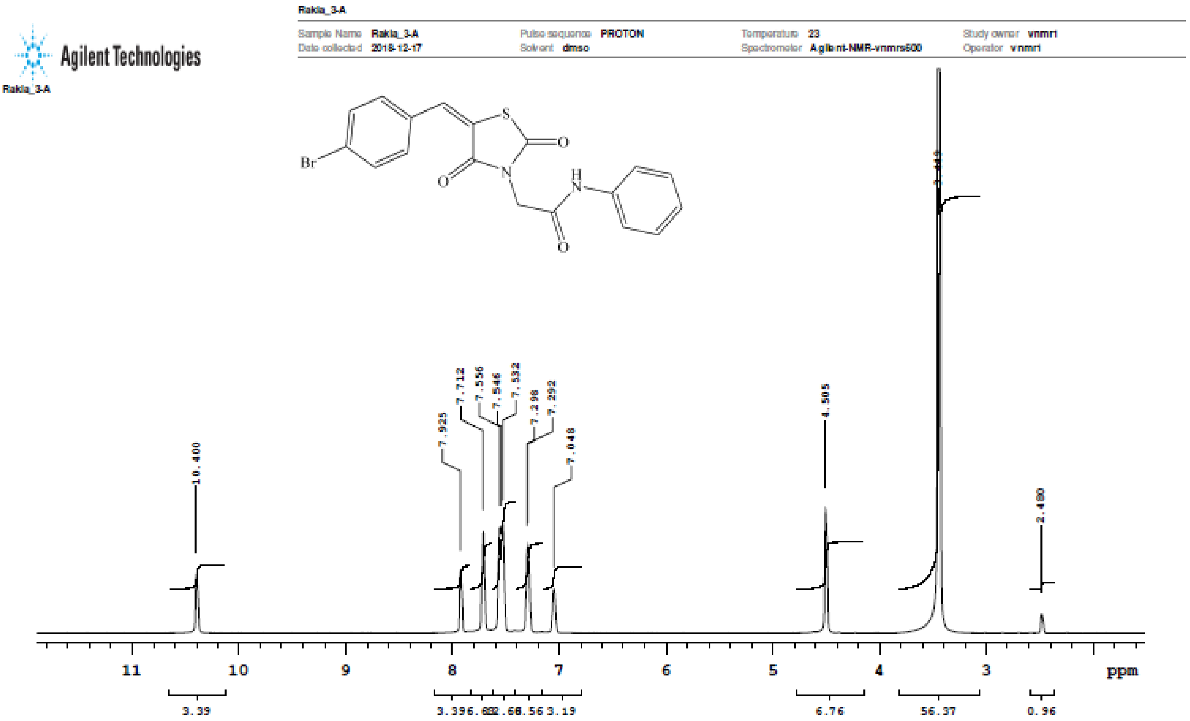

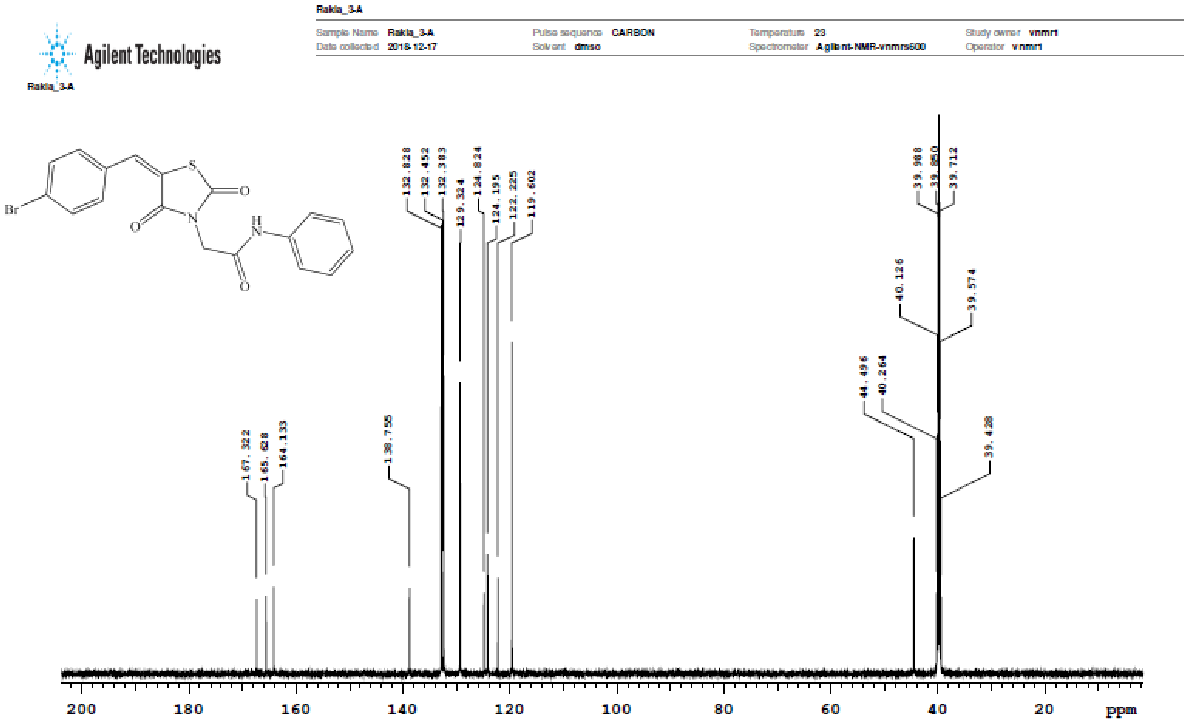

Figure S7 ^{1}H- and ^{13}C-NMR for compound **4d**

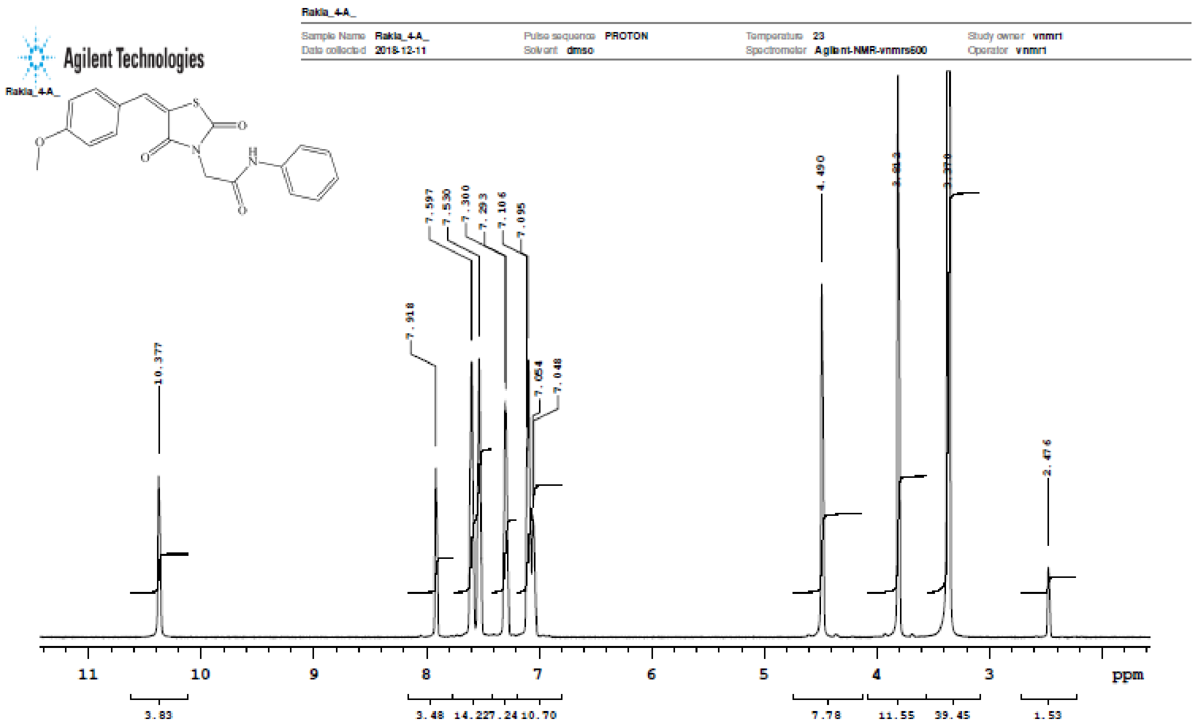

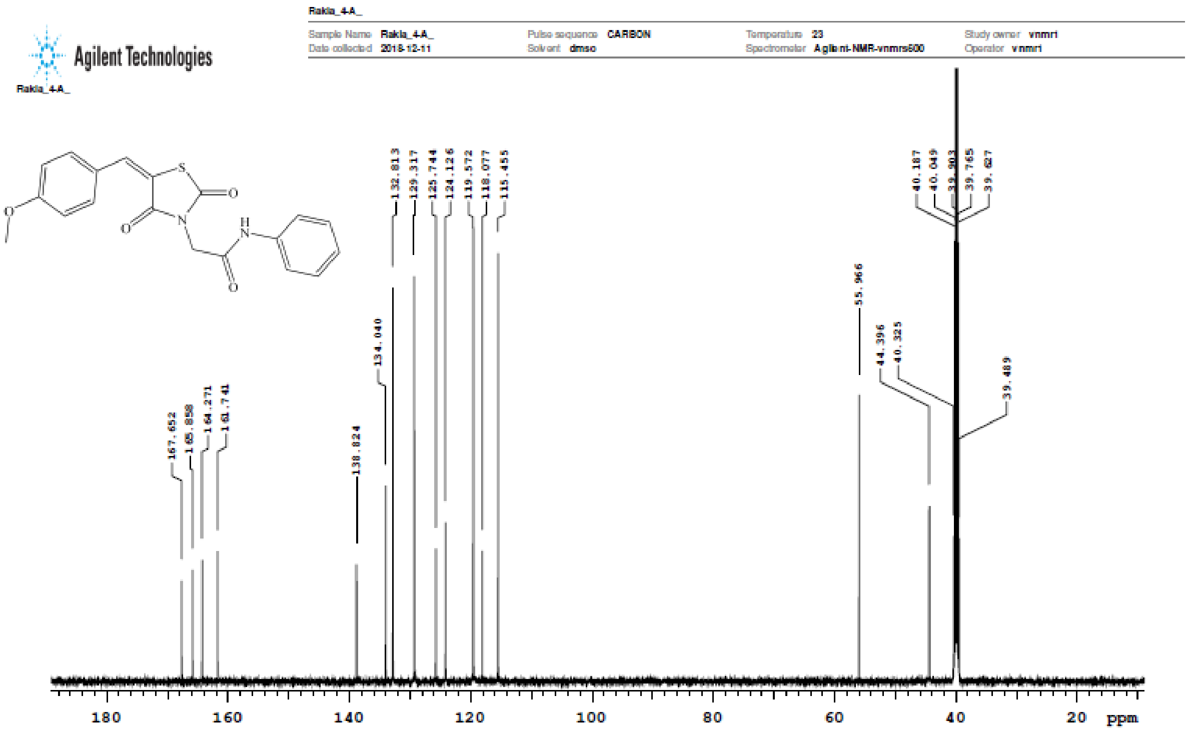

Figure S8 ^{1}H- and ^{13}C-NMR for compound **4e**

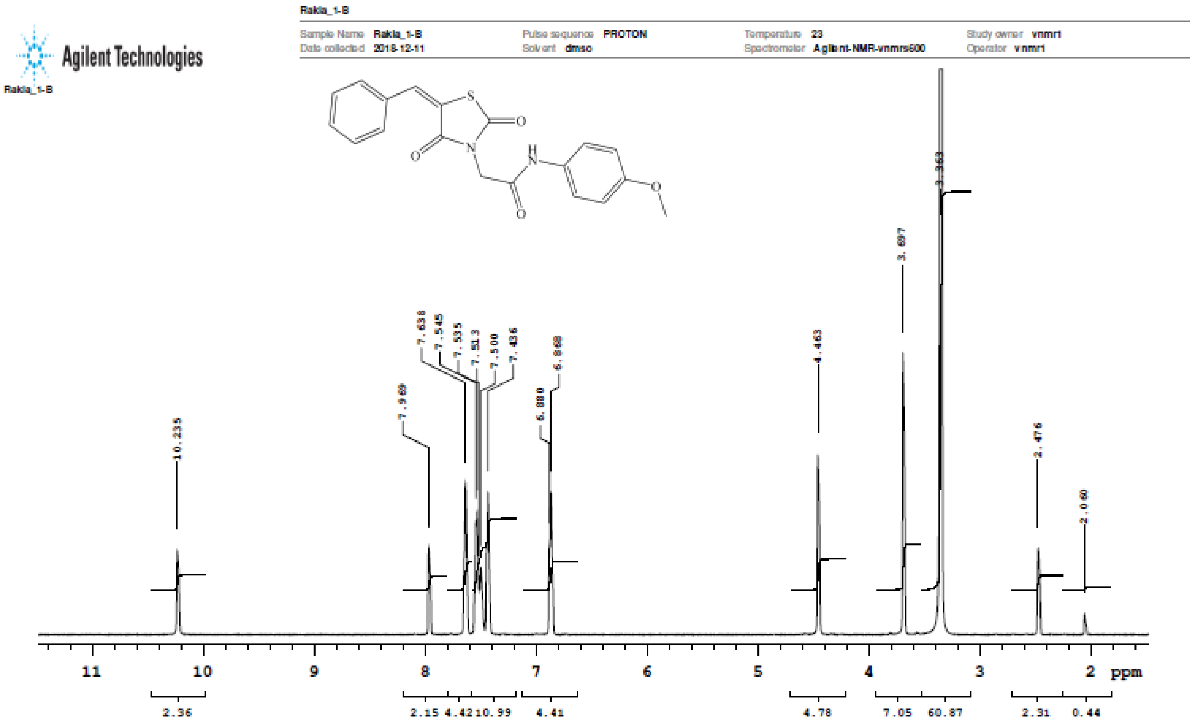

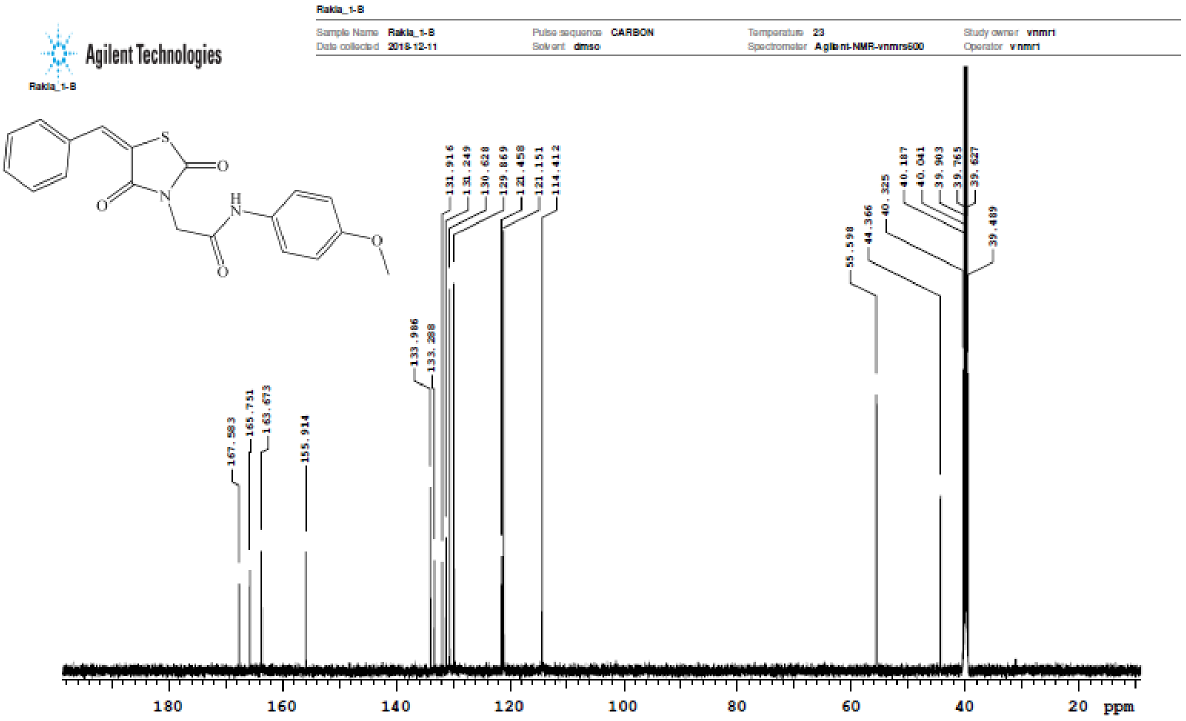

Figure S9 ^{1}H- and ^{13}C-NMR for compound **4f**

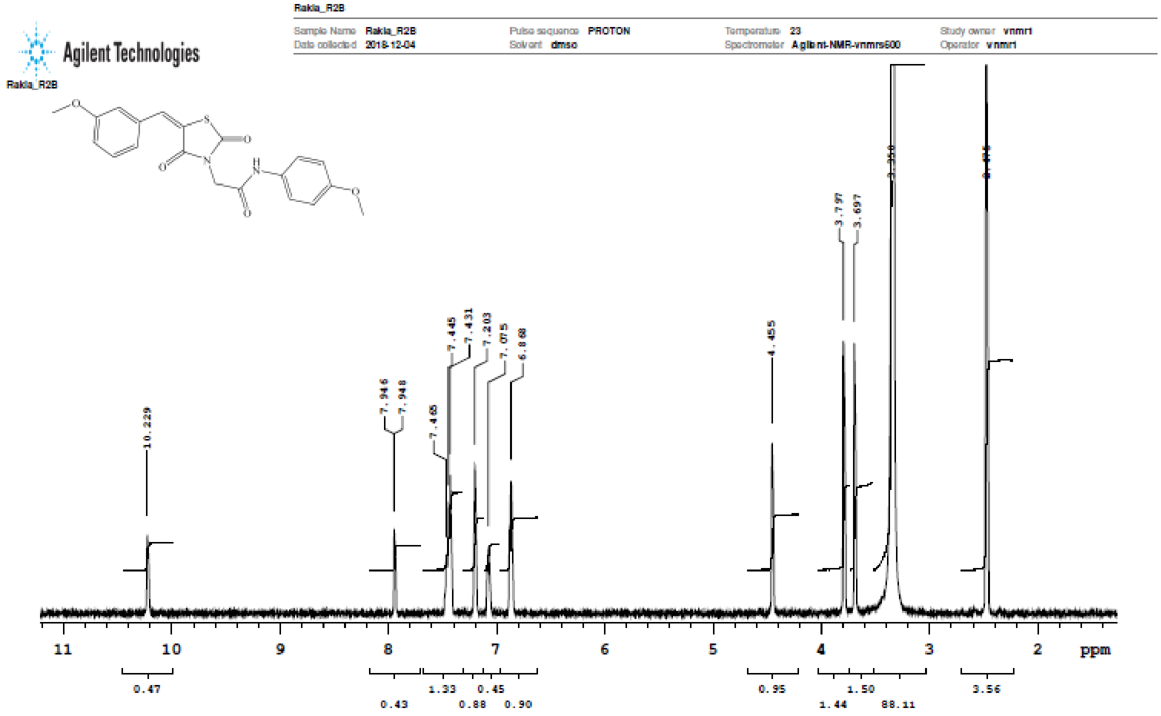

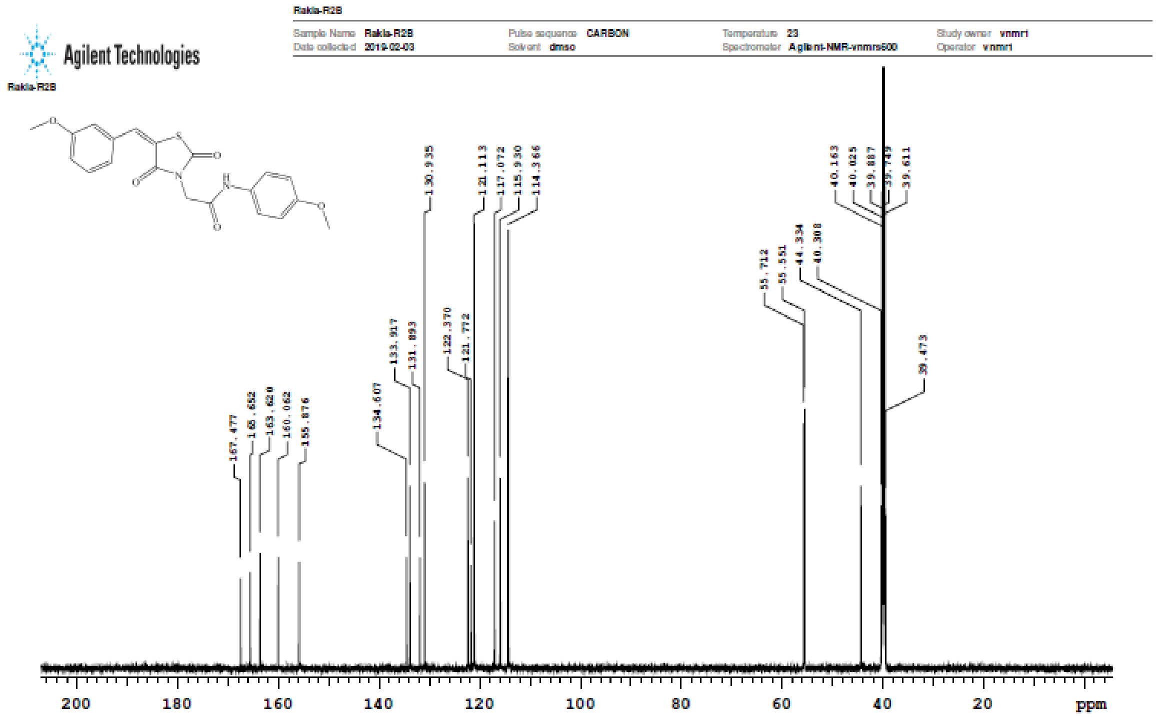

Figure S10 ^{1}H- and ^{13}C-NMR for compound **4g**

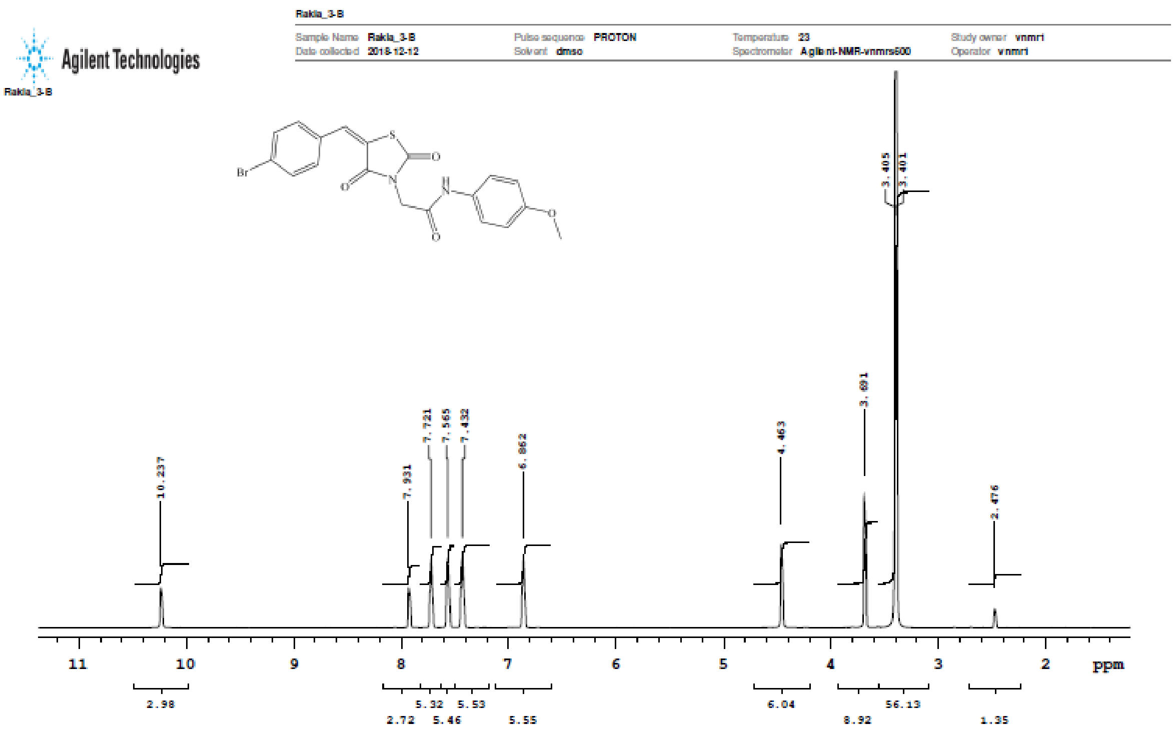

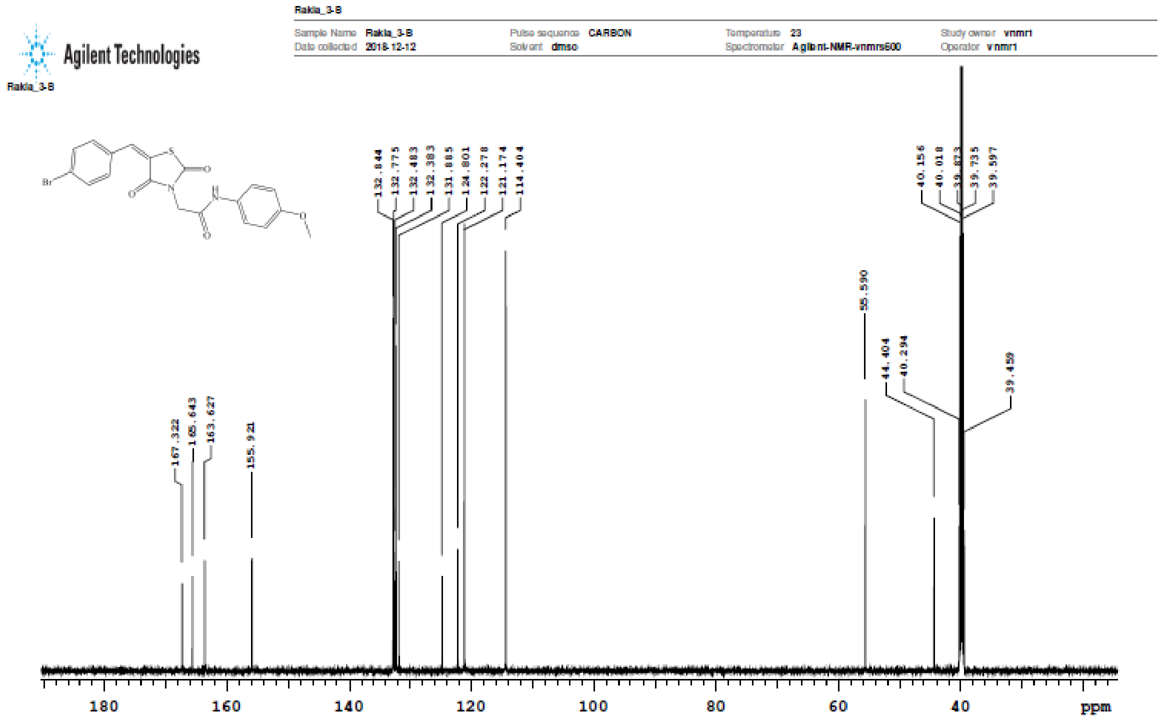

Figure S11 ^{1}H- and ^{13}C-NMR for compound **4h**

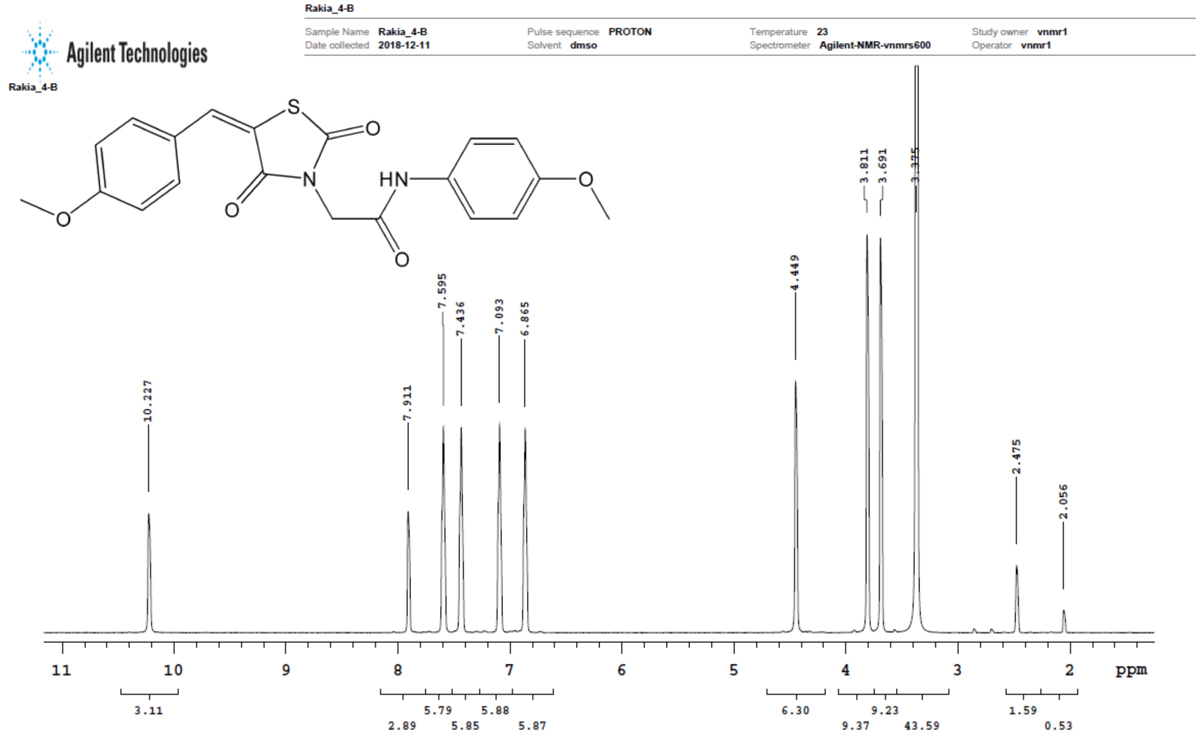

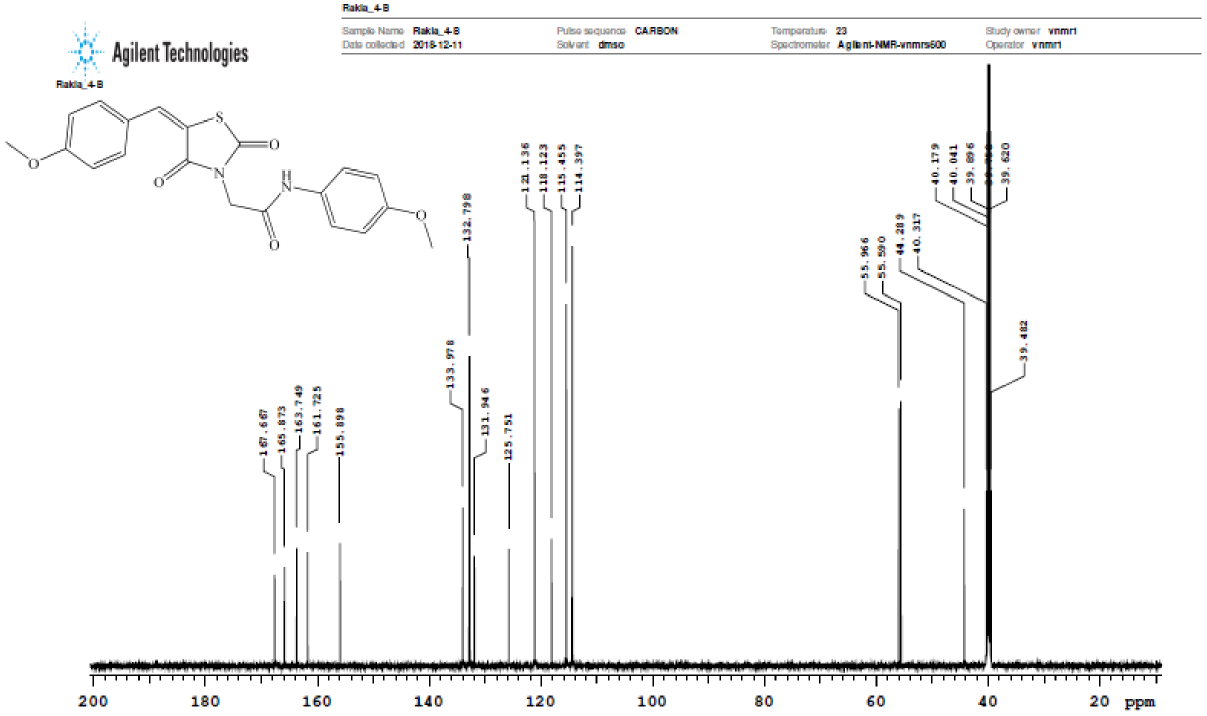

Figure S12 ^{1}H- and ^{13}C-NMR for compound **4i**

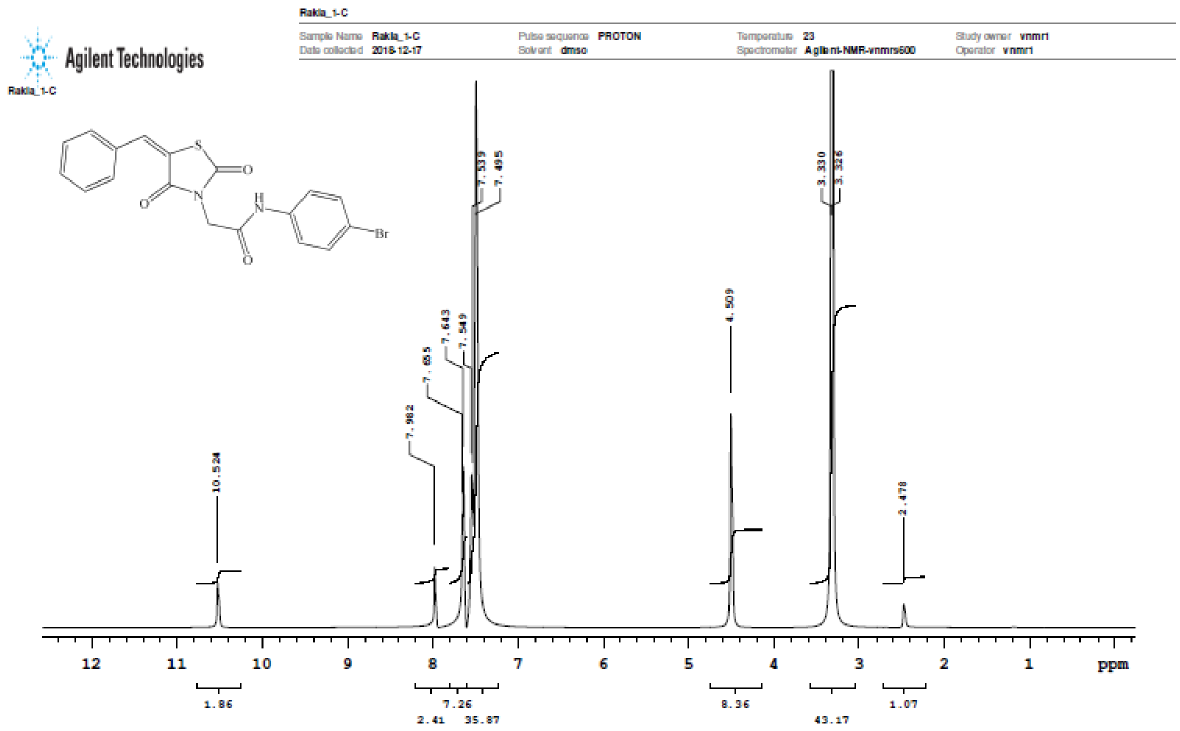

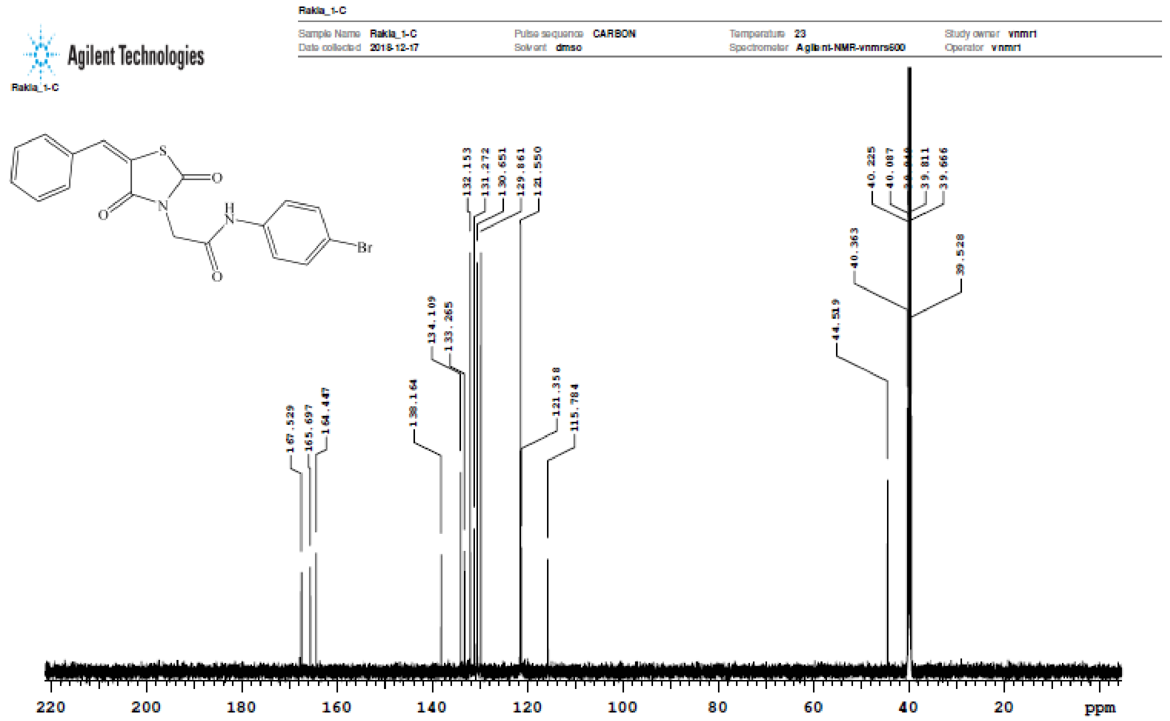

Figure S13 ^{1}H- and ^{13}C-NMR for compound **4j**

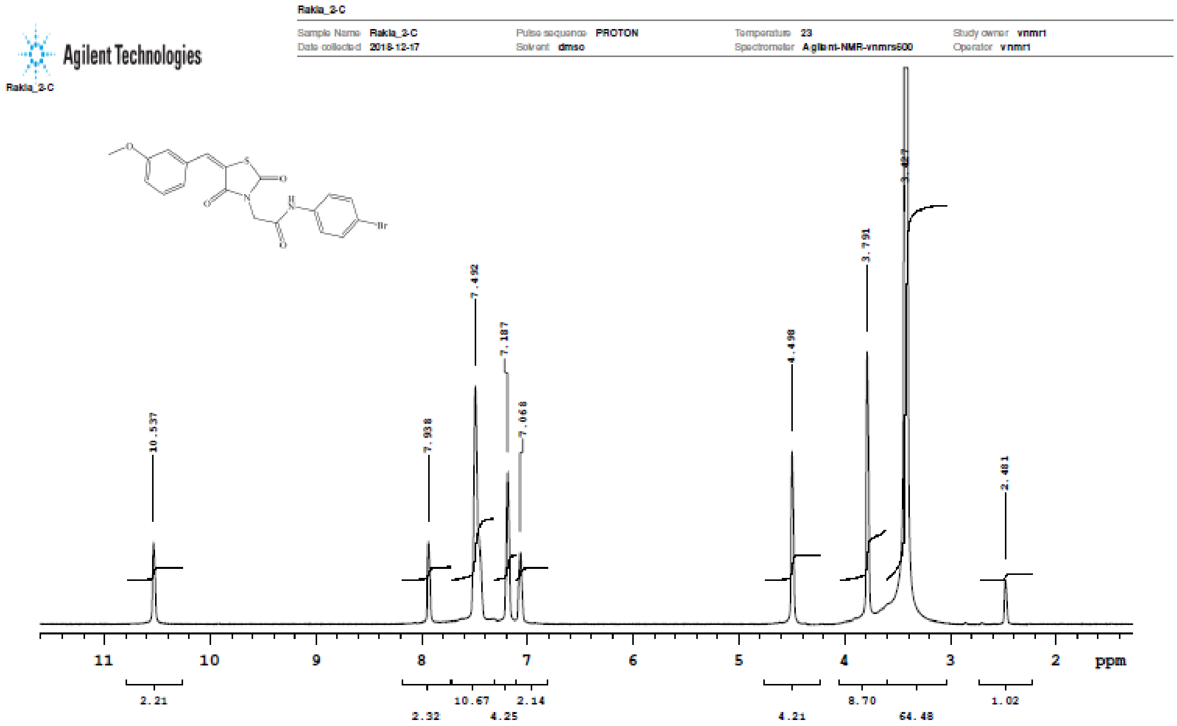

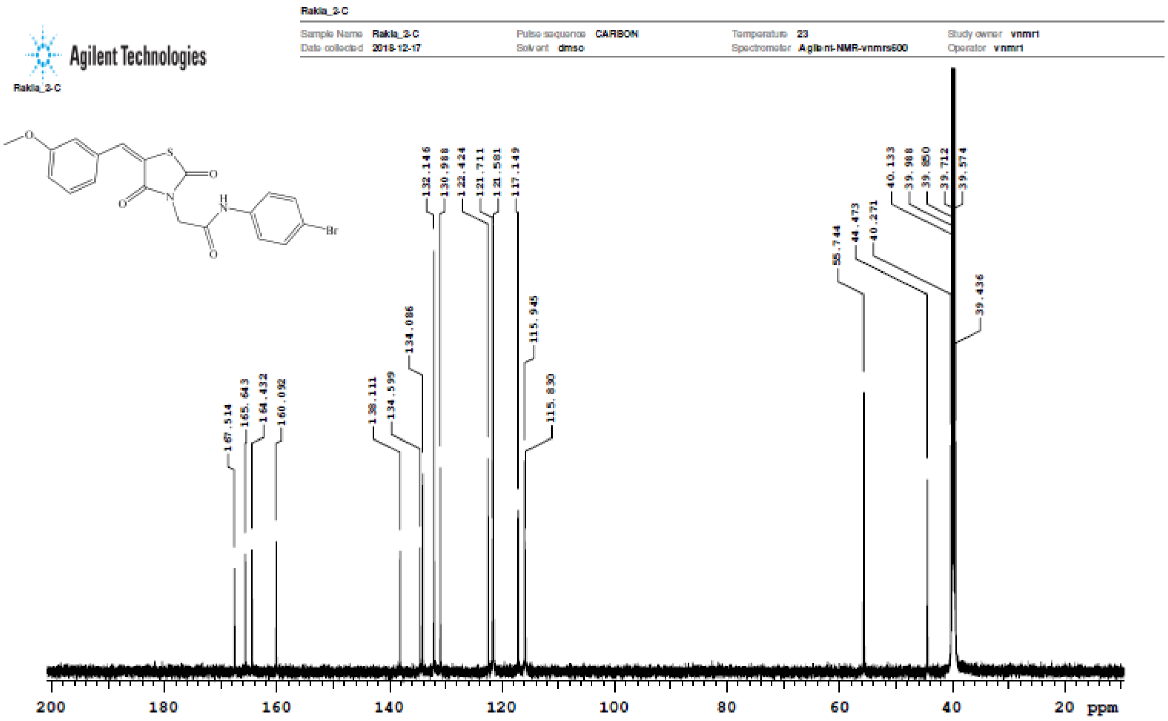

Figure S14 ^{1}H- and ^{13}C-NMR for compound **4k**

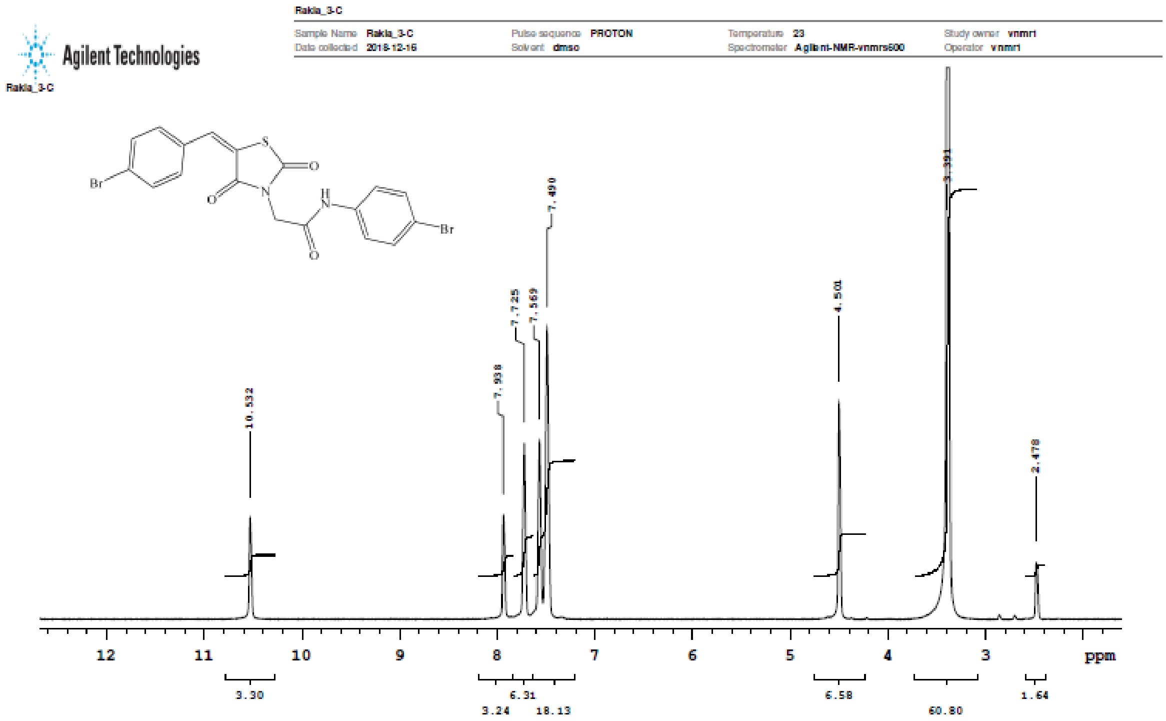

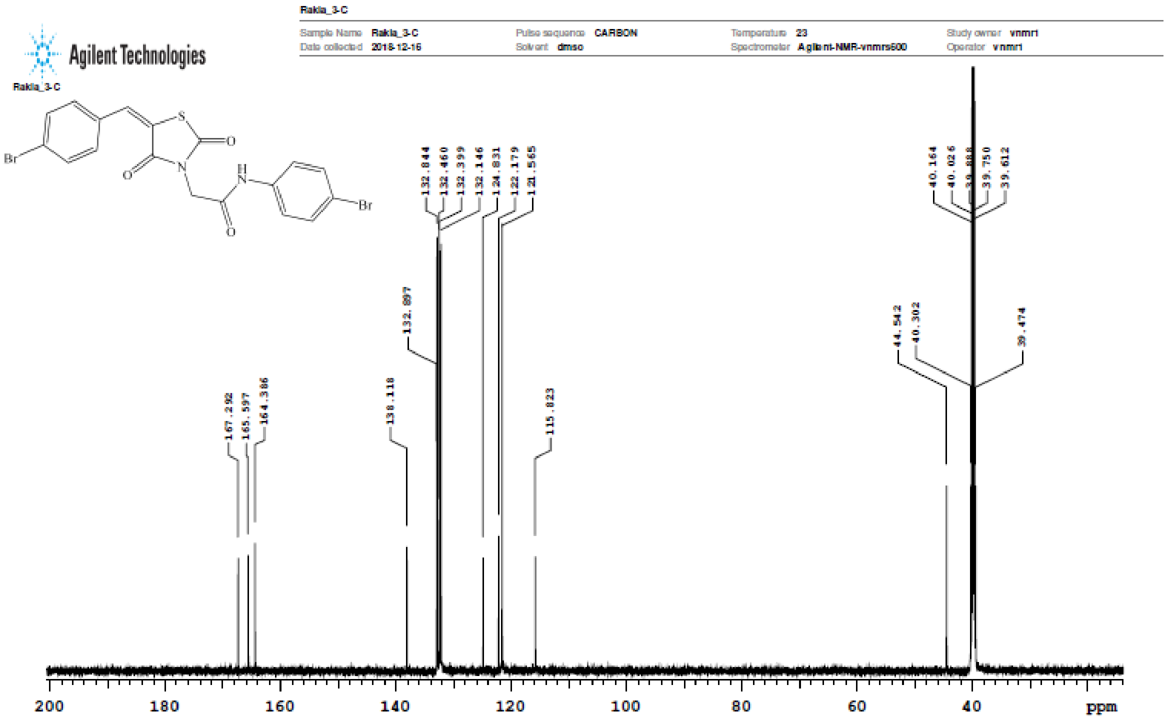

Figure S15 ^{1}H- and ^{13}C-NMR for compound **4l**

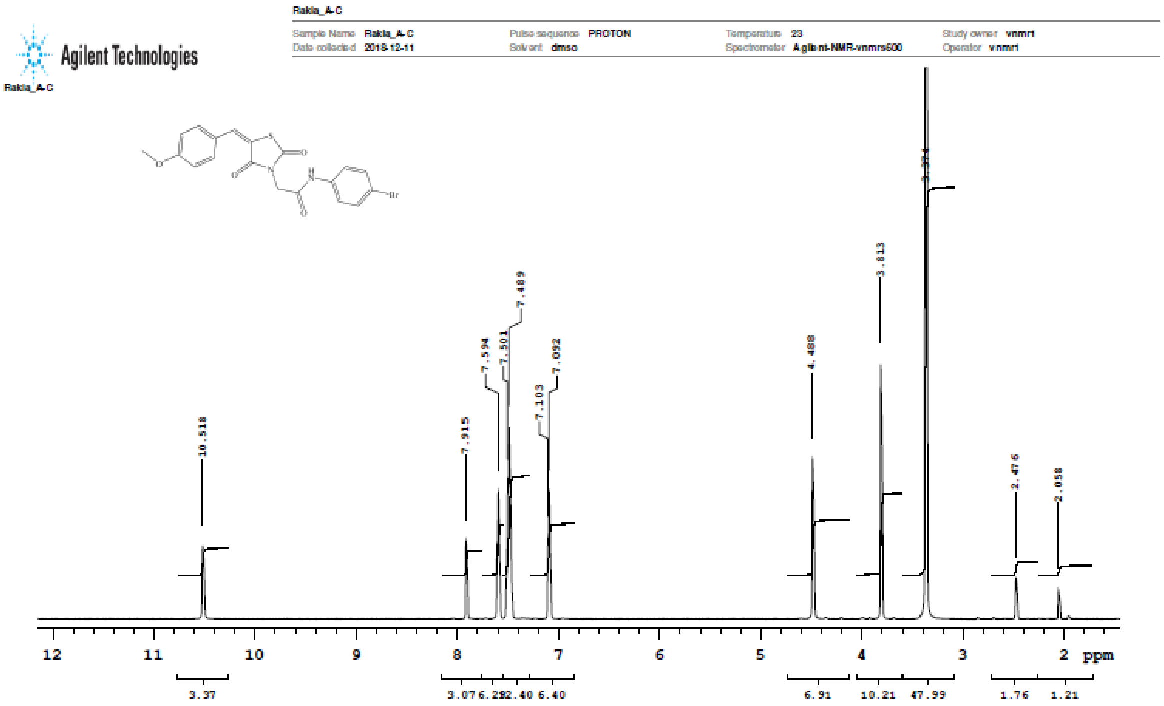

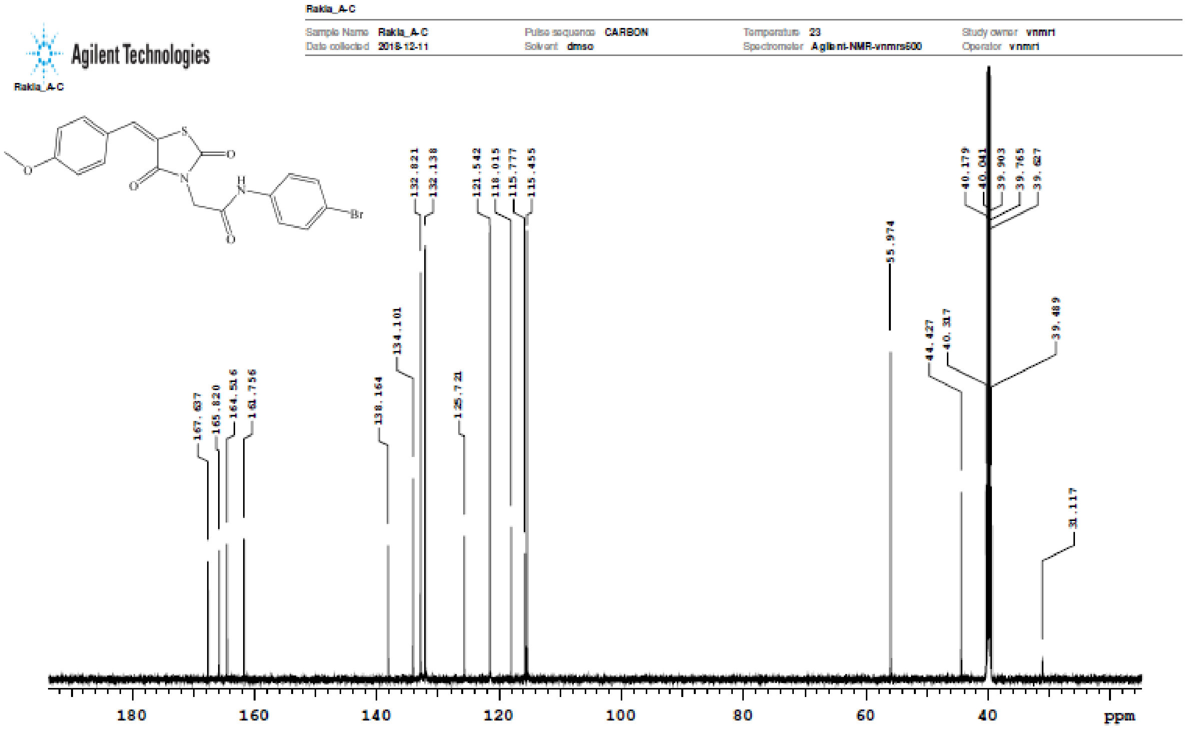

Figure S16 ^{1}H- and ^{13}C-NMR for compound **4m**

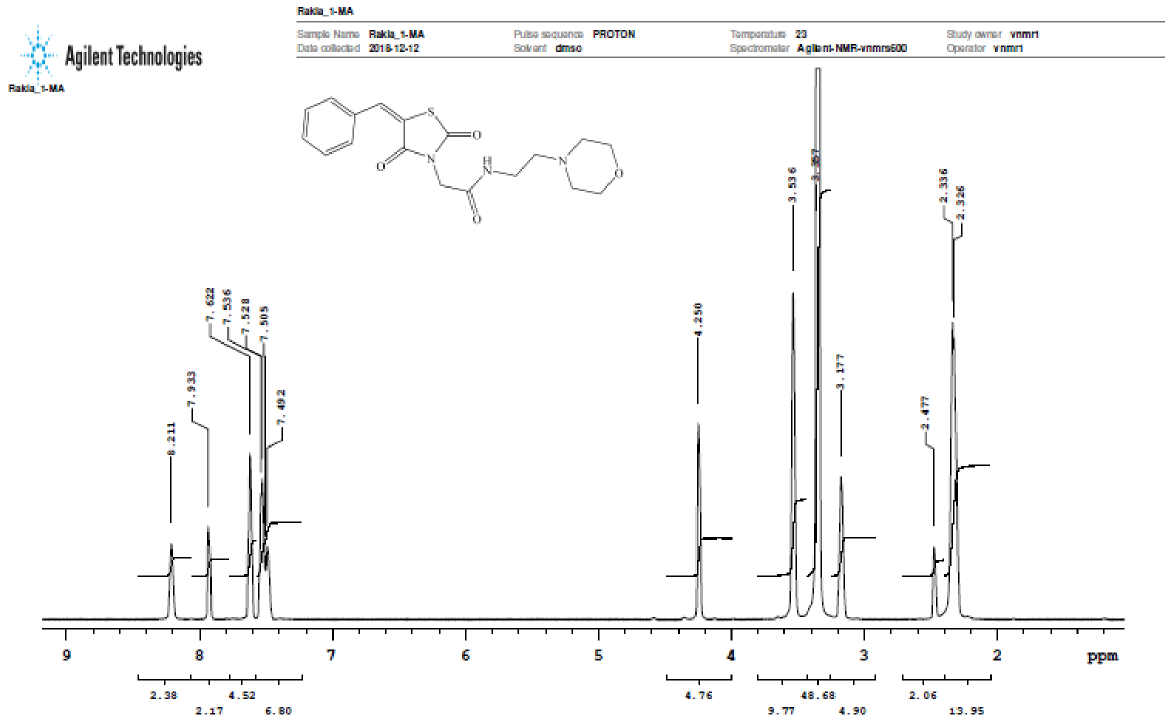

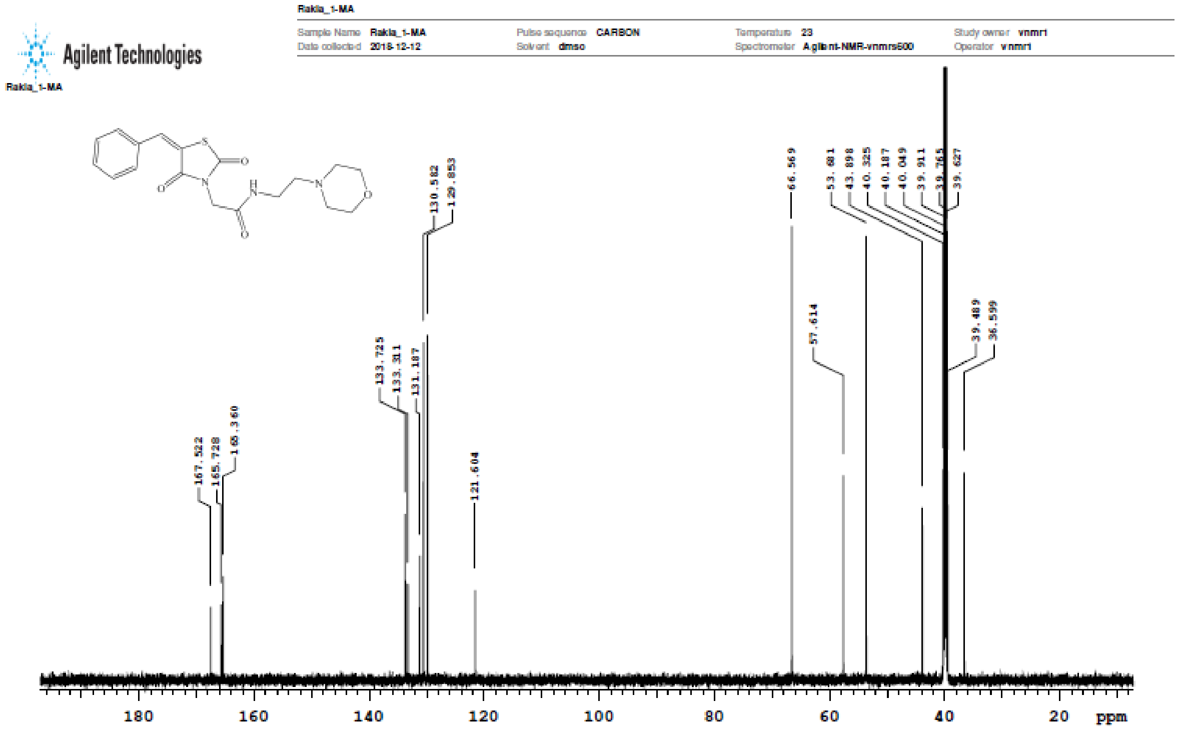

Figure S17 ^{1}H- and ^{13}C-NMR for compound **4n**

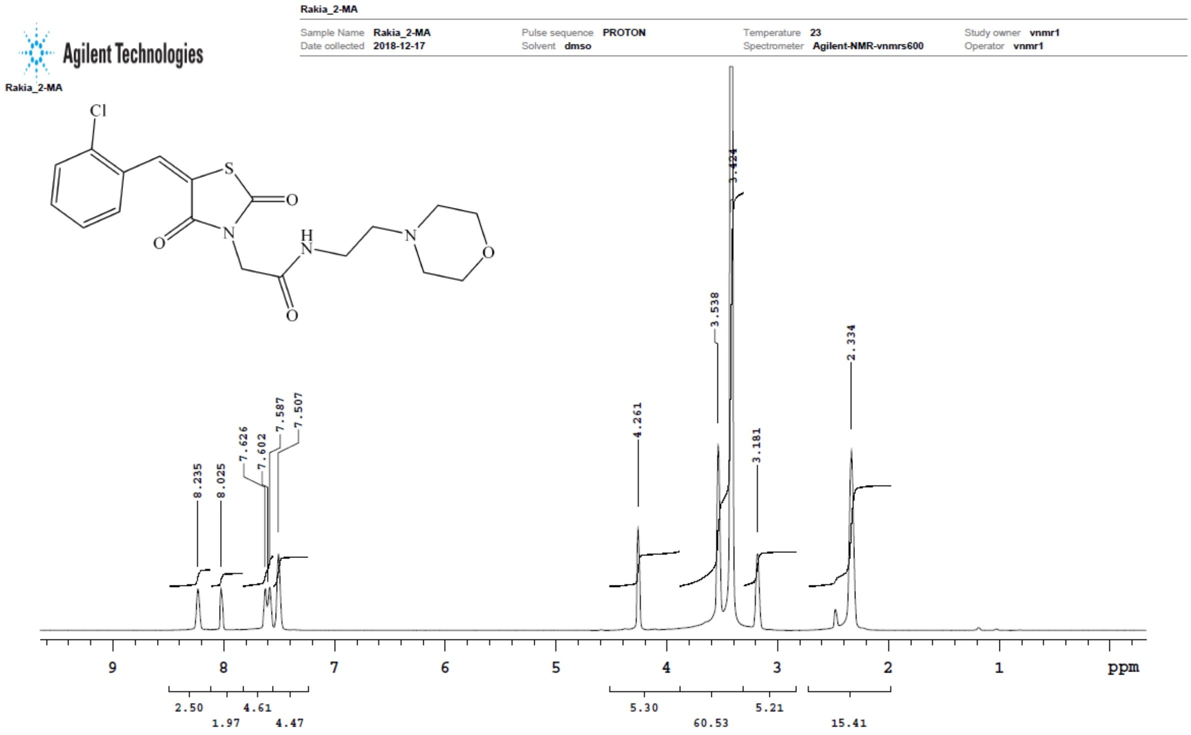

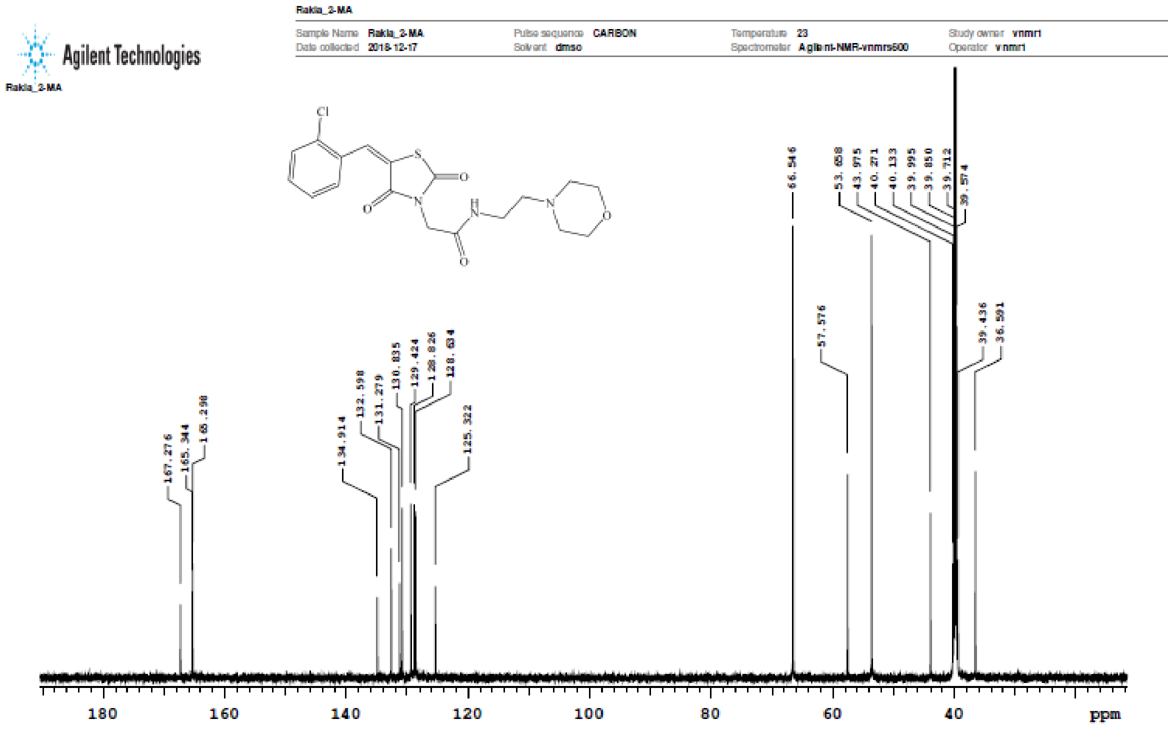

Figure S18 ^{1}H- and ^{13}C-NMR for compound **4o**

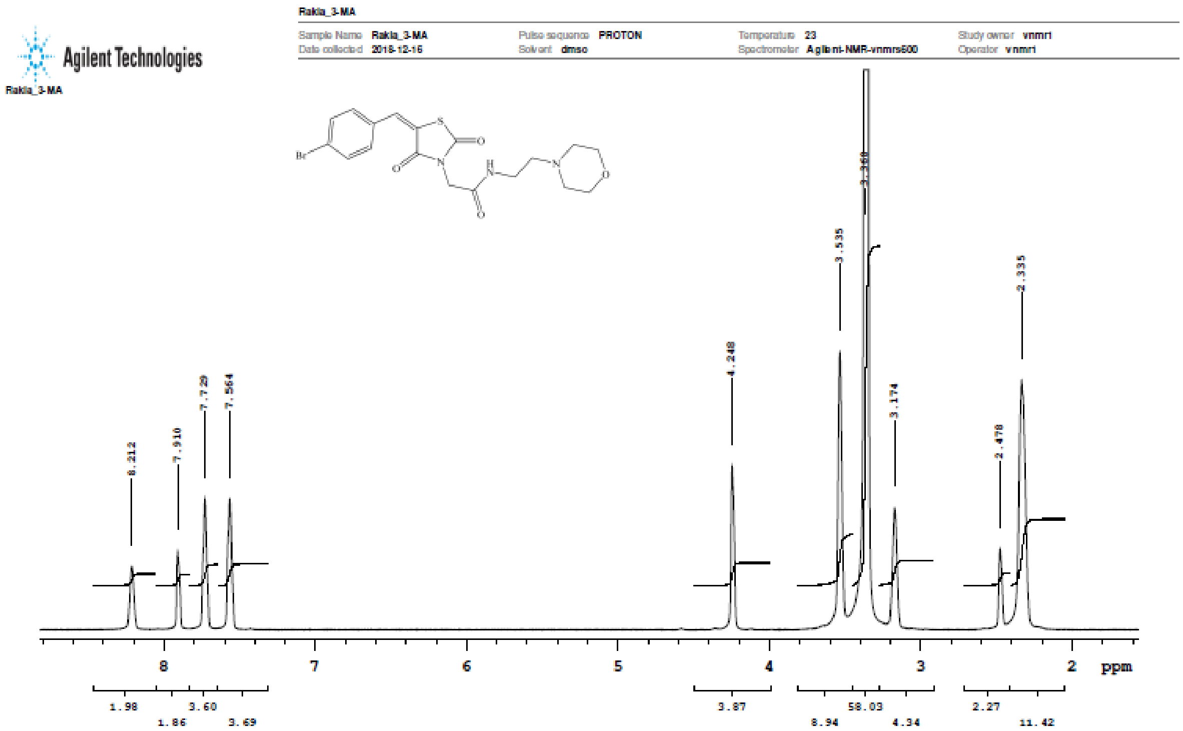

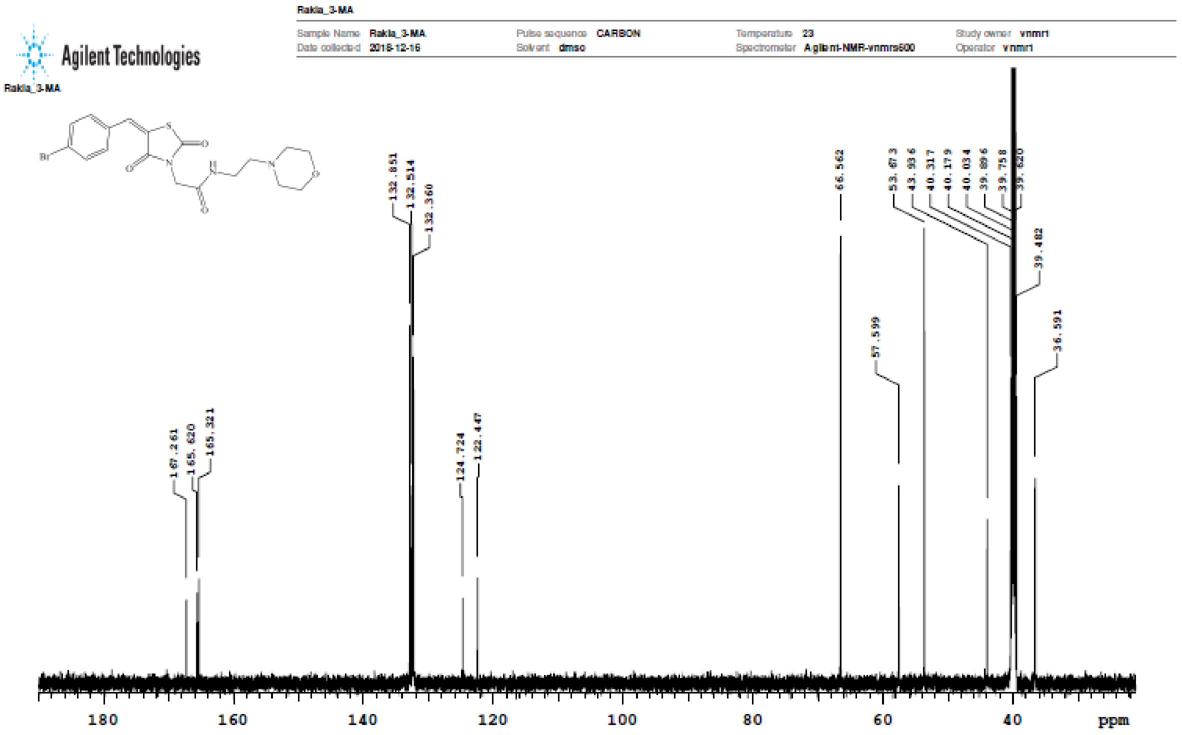

Figure S19 ^{1}H- and ^{13}C-NMR for compound **4p**

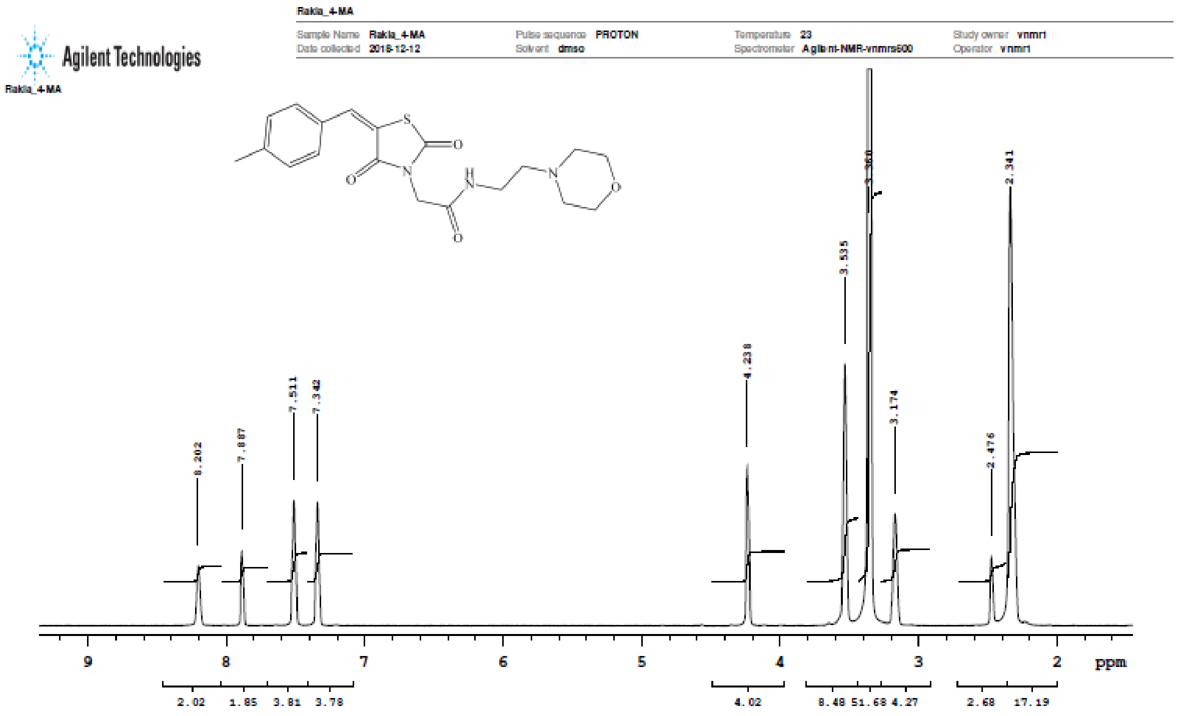

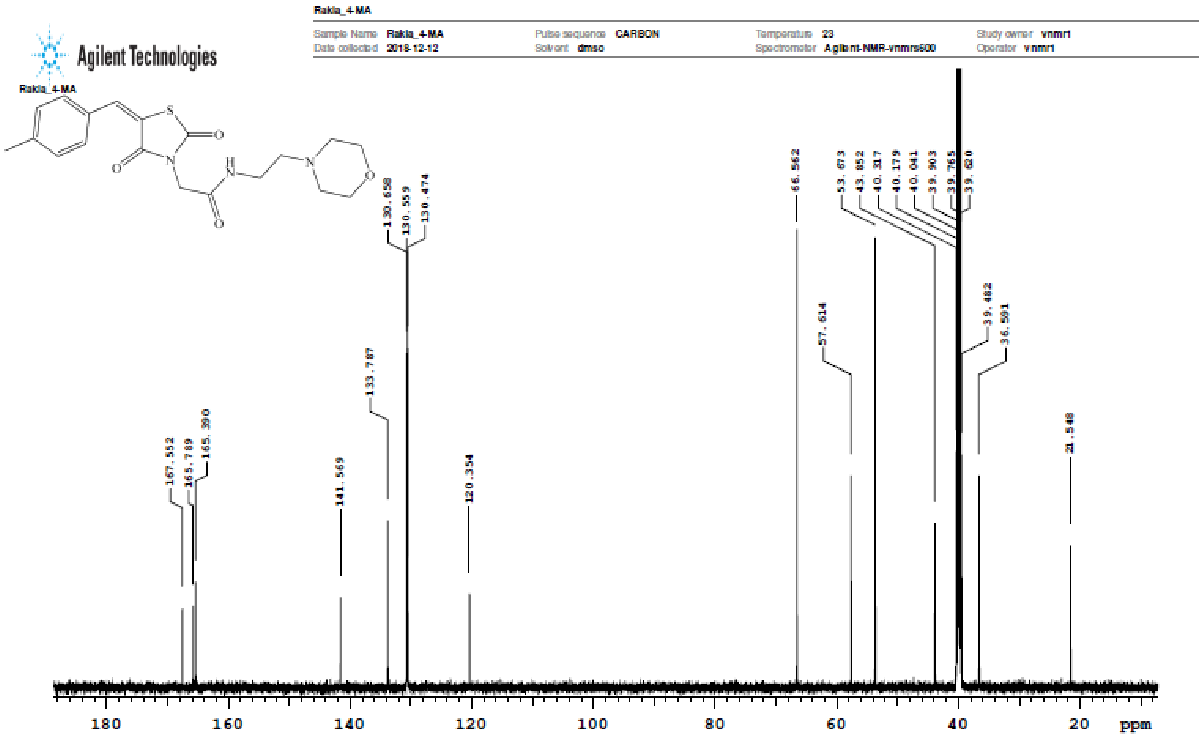

Figure S20 ^{1}H- and ^{13}C-NMR for compound **4q**

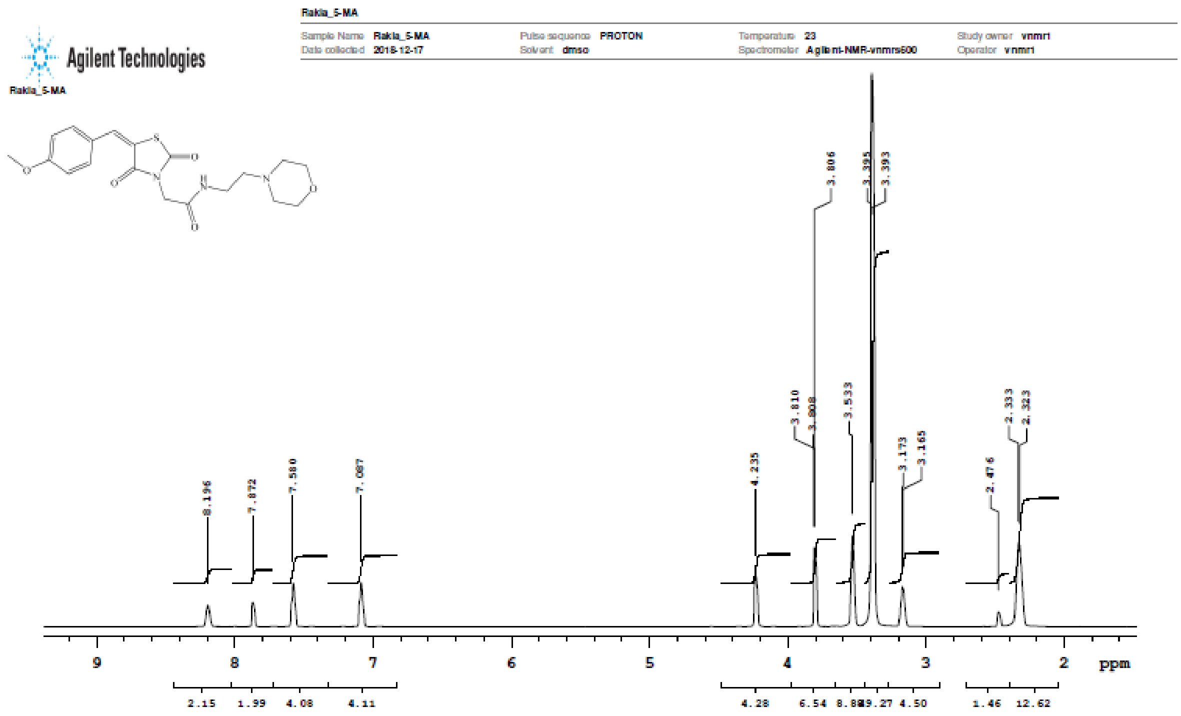

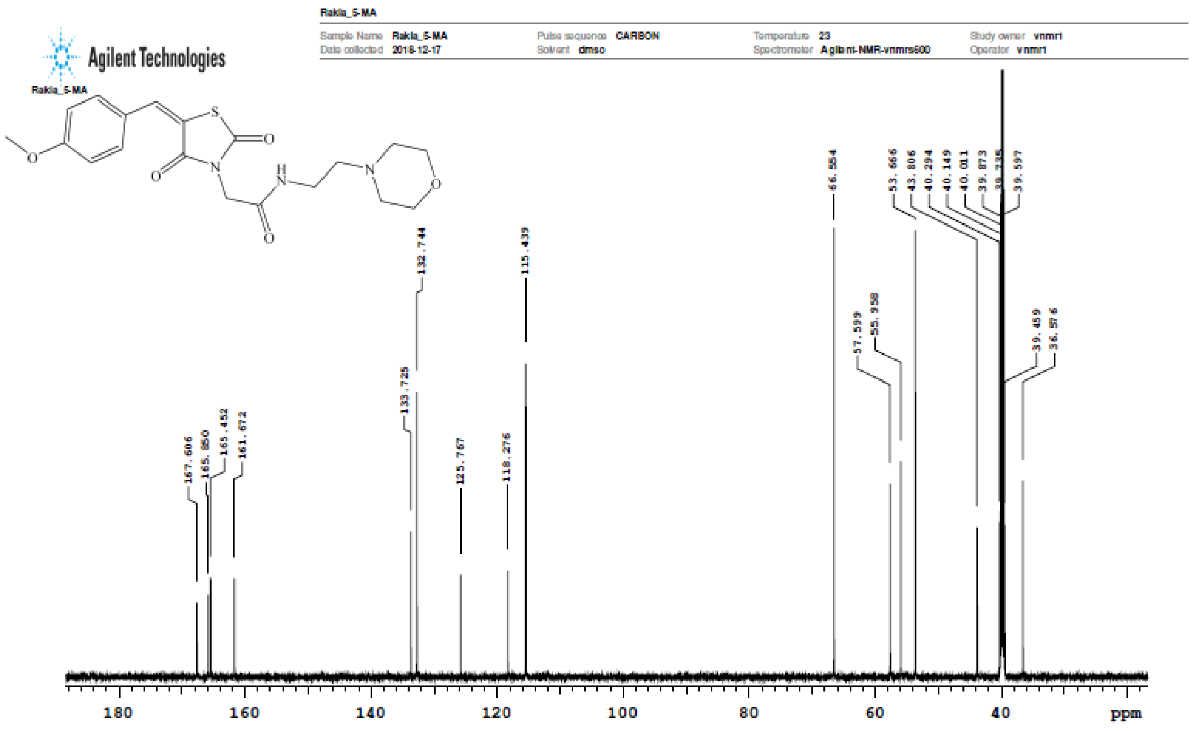

Figure S21 ^{1}H- and ^{13}C-NMR for compound **4r**

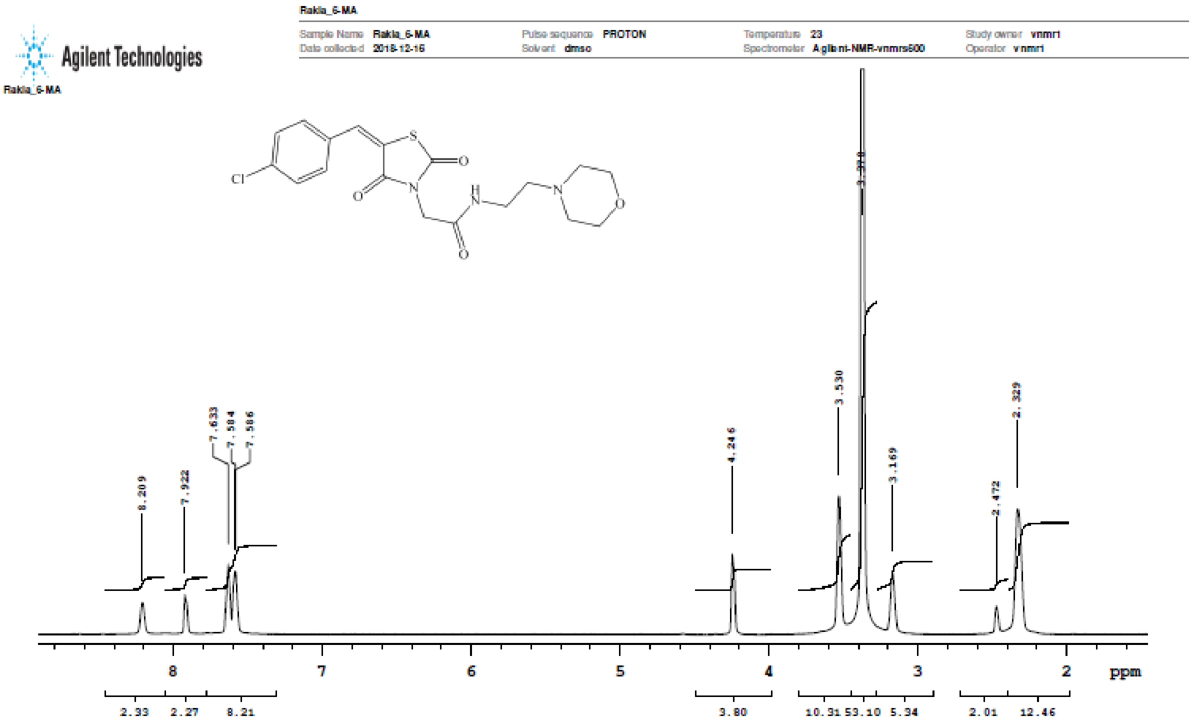

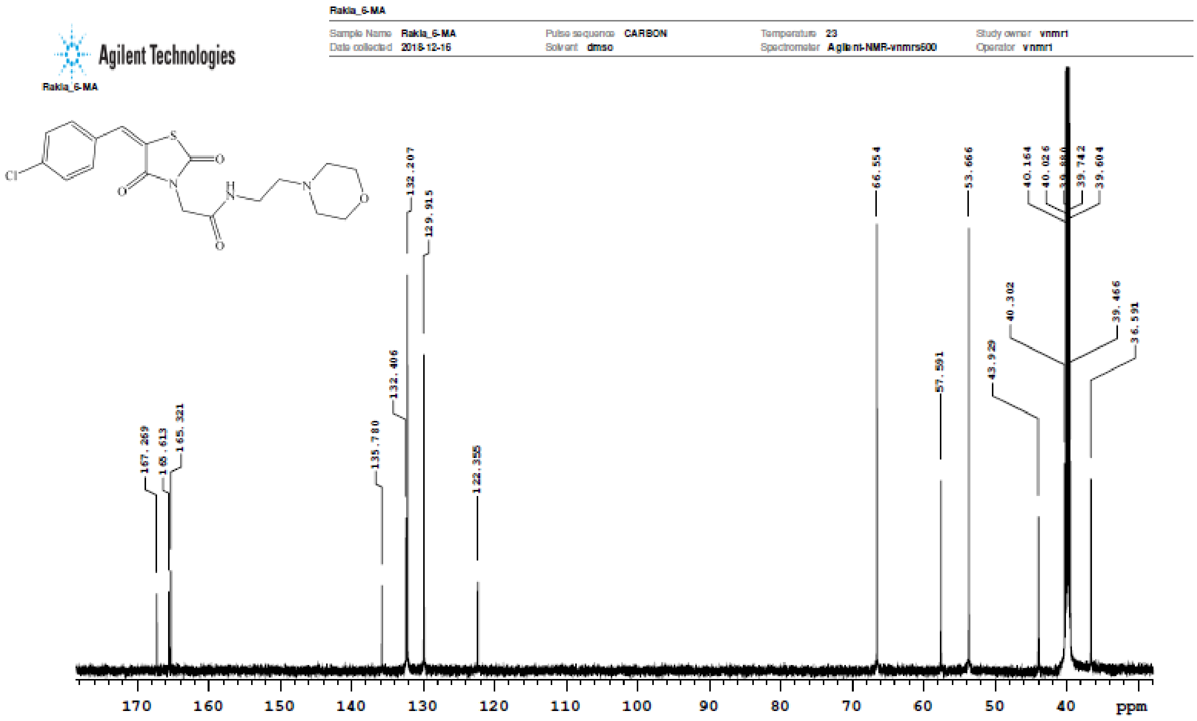

Figure S22 ^{1}H- and ^{13}C-NMR for compound **4s**

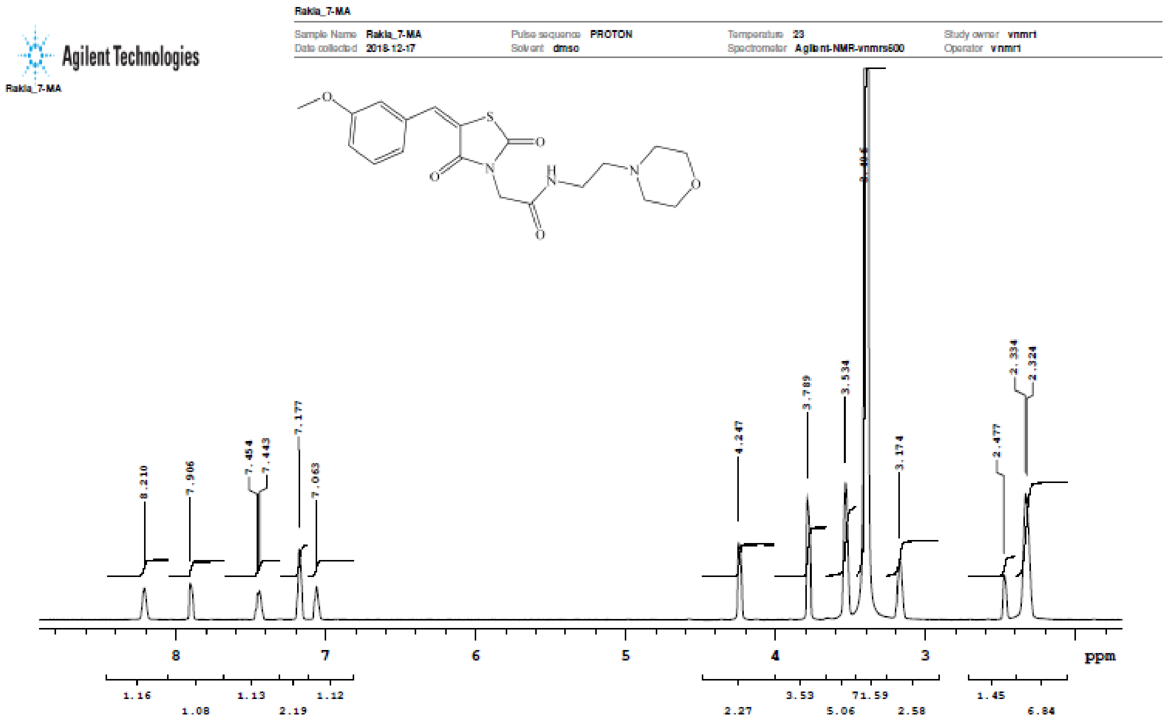

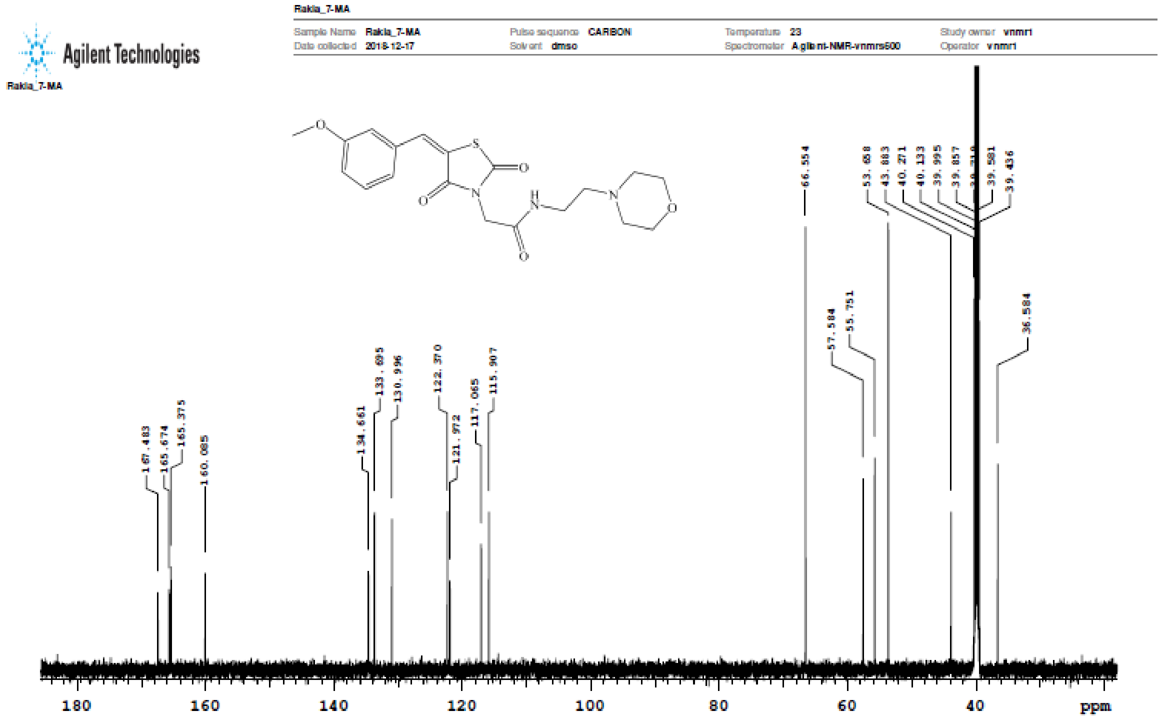

Figure S23 ^{1}H- and ^{13}C-NMR for compound **5a**

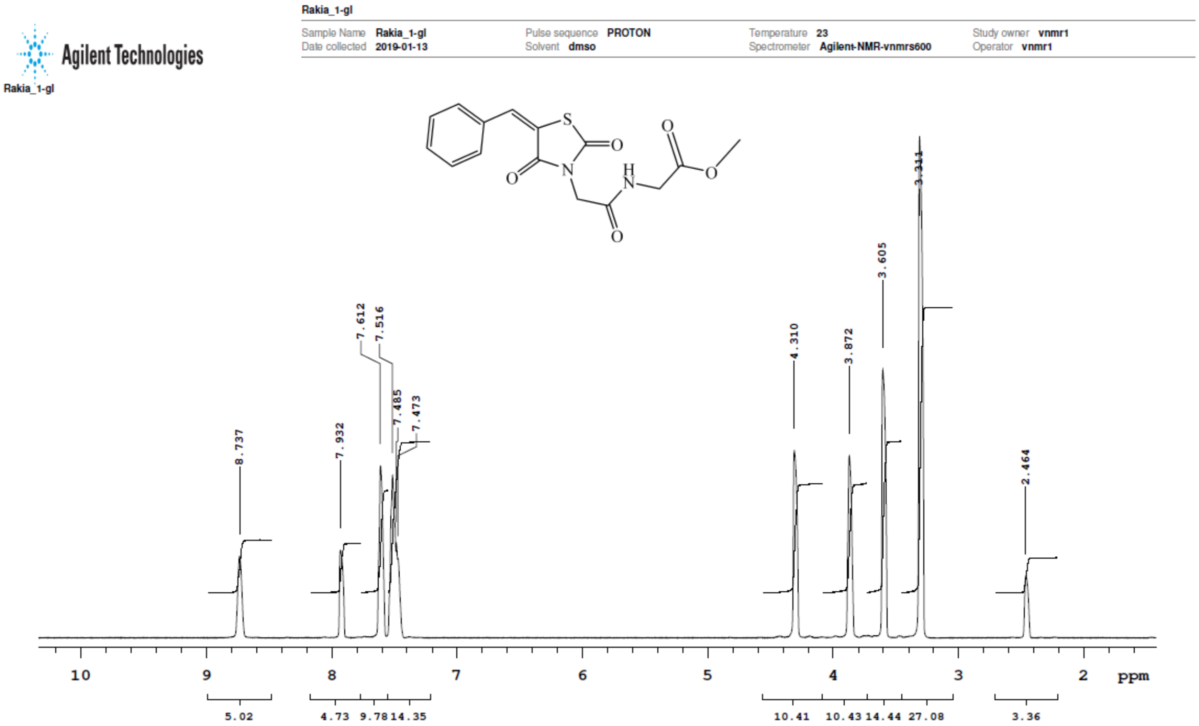

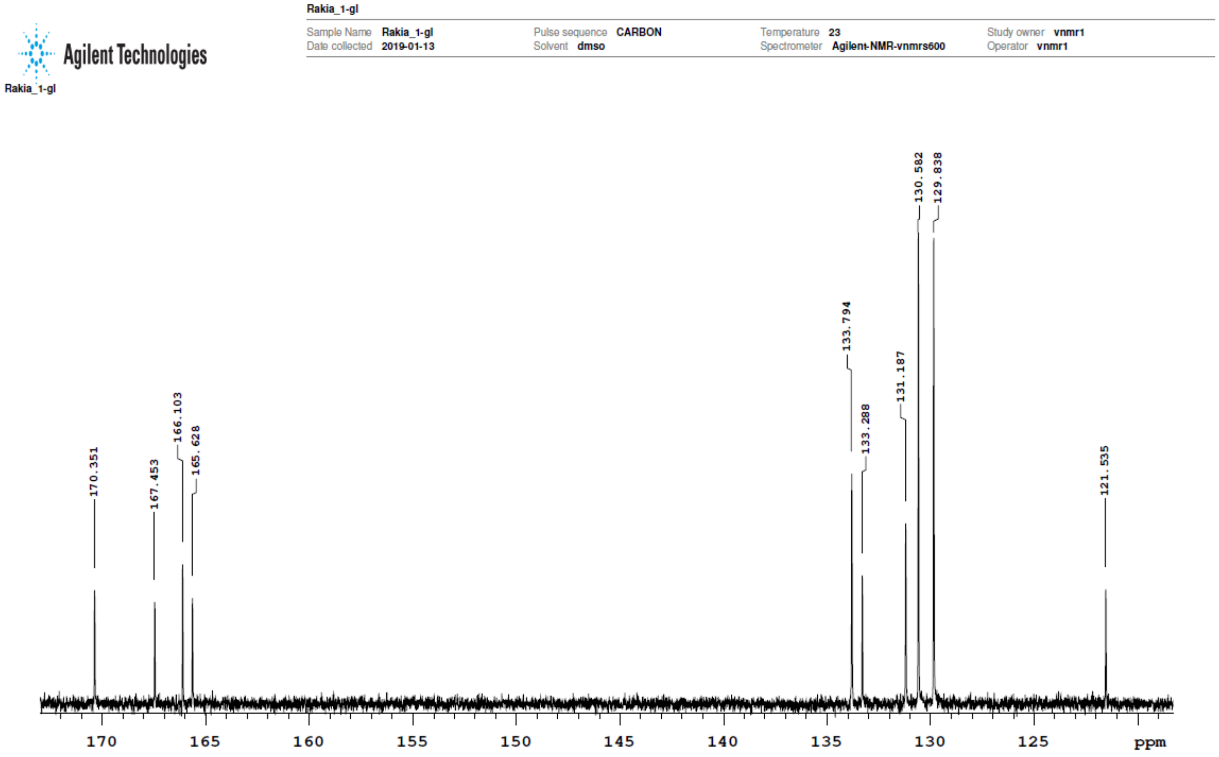

Figure S24 ^{1}H- and ^{13}C-NMR for compound **5b**

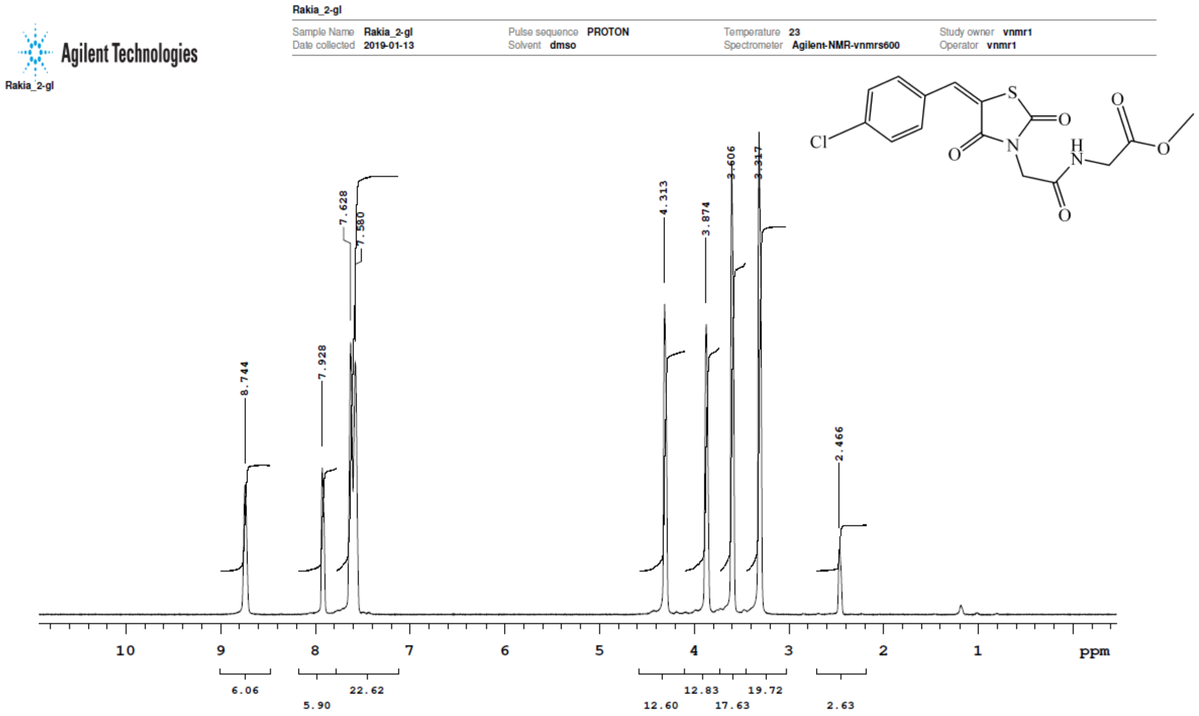

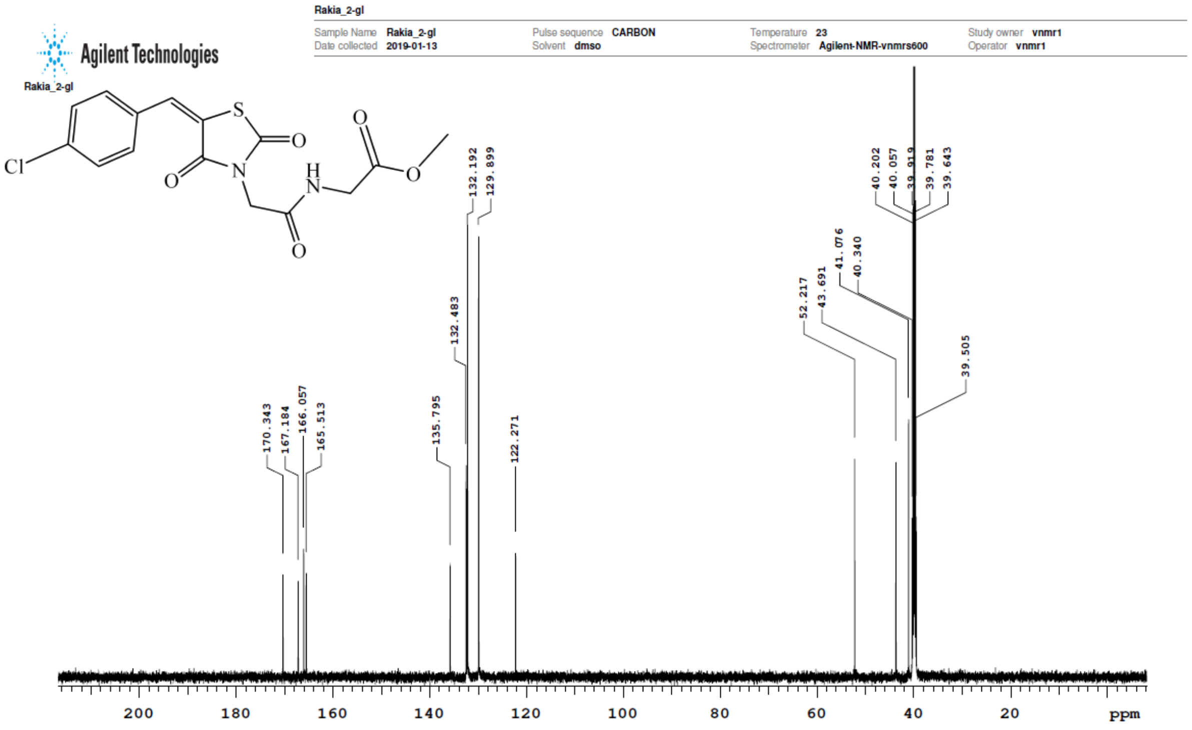

Figure S25 ^{1}H- and ^{13}C-NMR for compound **5c**

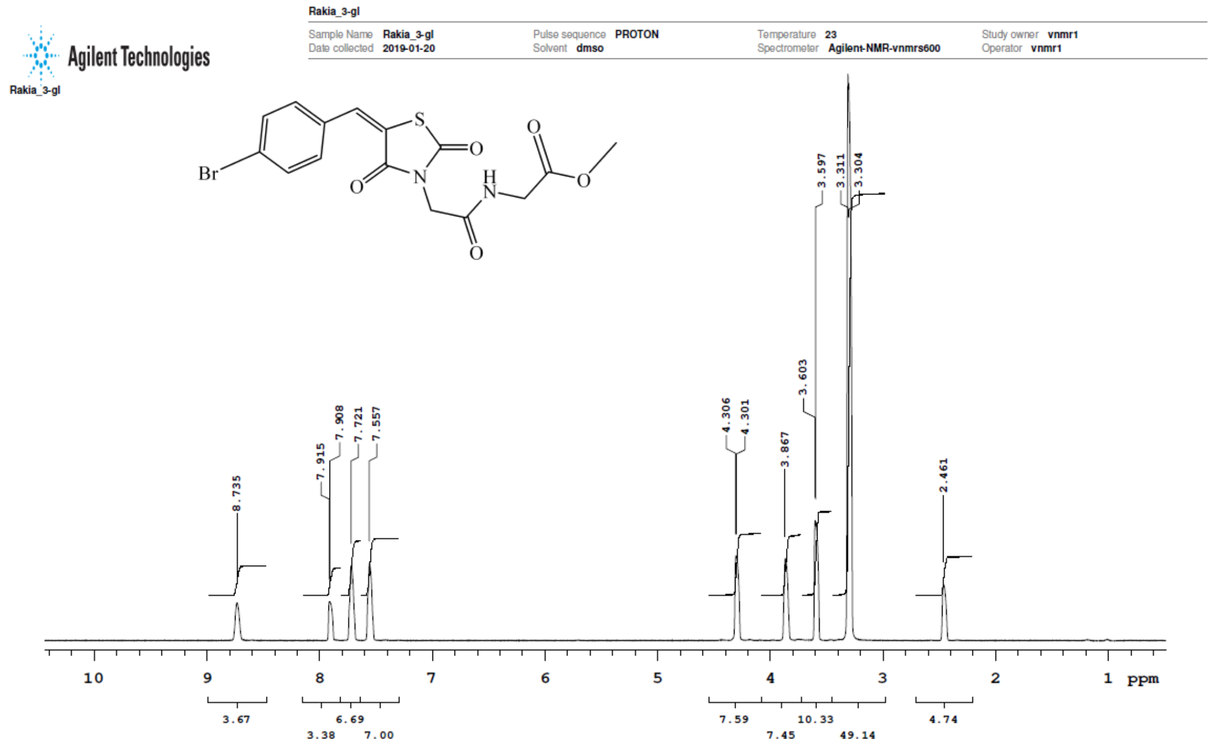

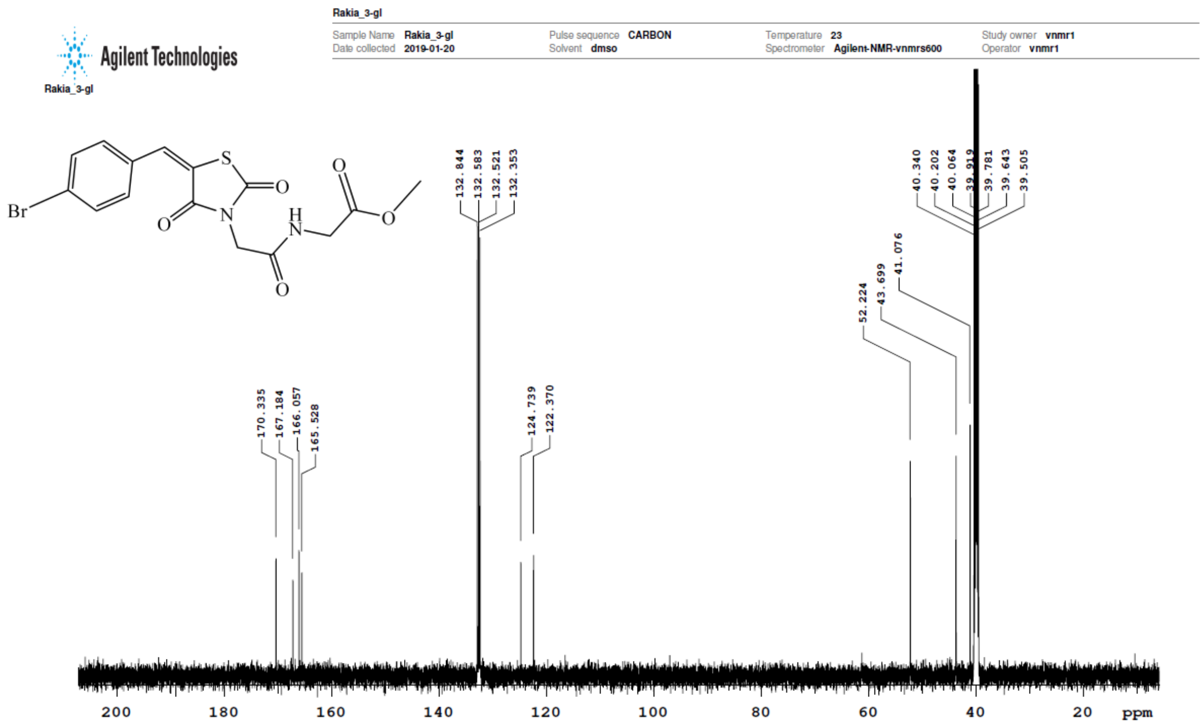

Figure S26 ^{1}H- and ^{13}C-NMR for compound **5d**

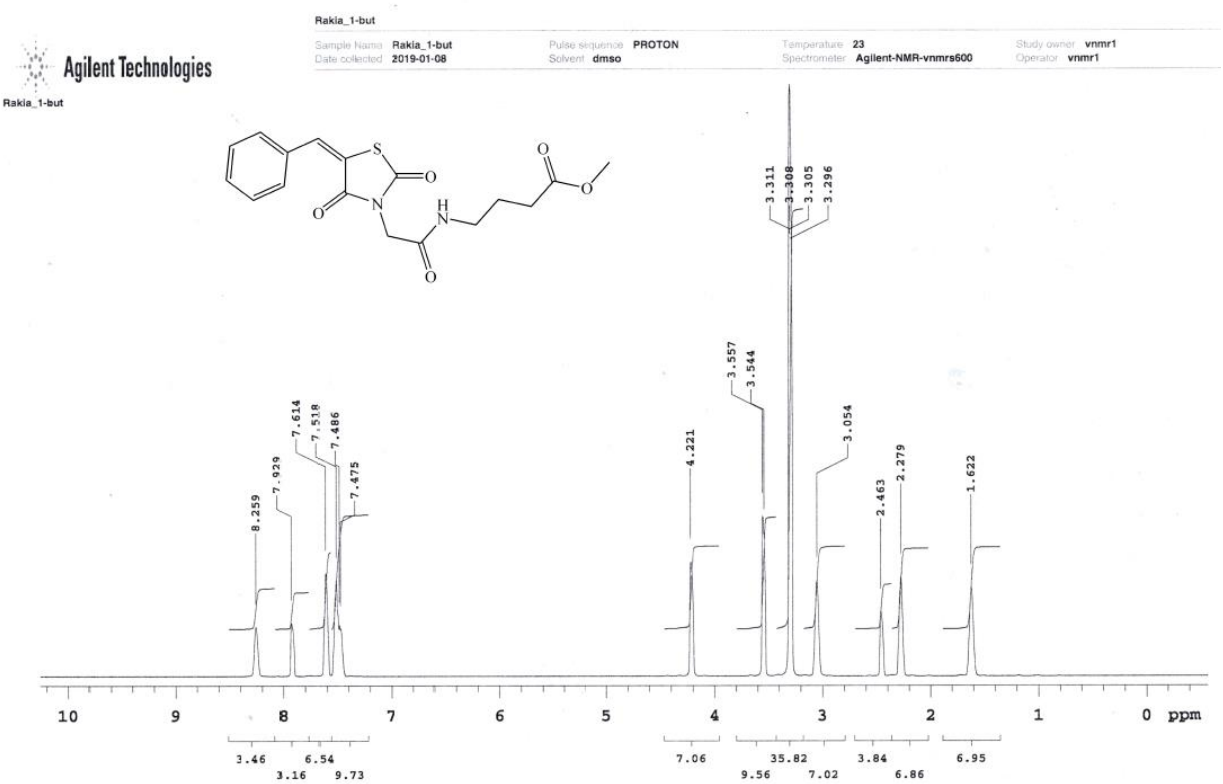

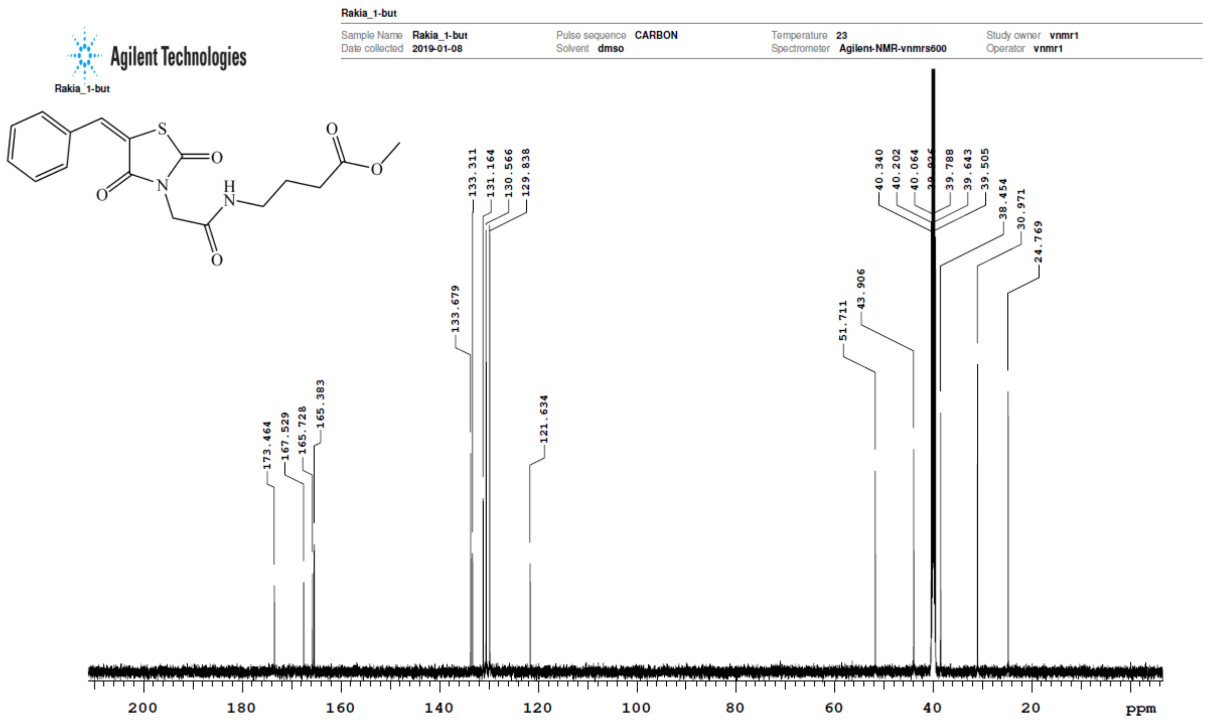

Figure S27 ^{1}H- and ^{13}C-NMR for compound **5e**

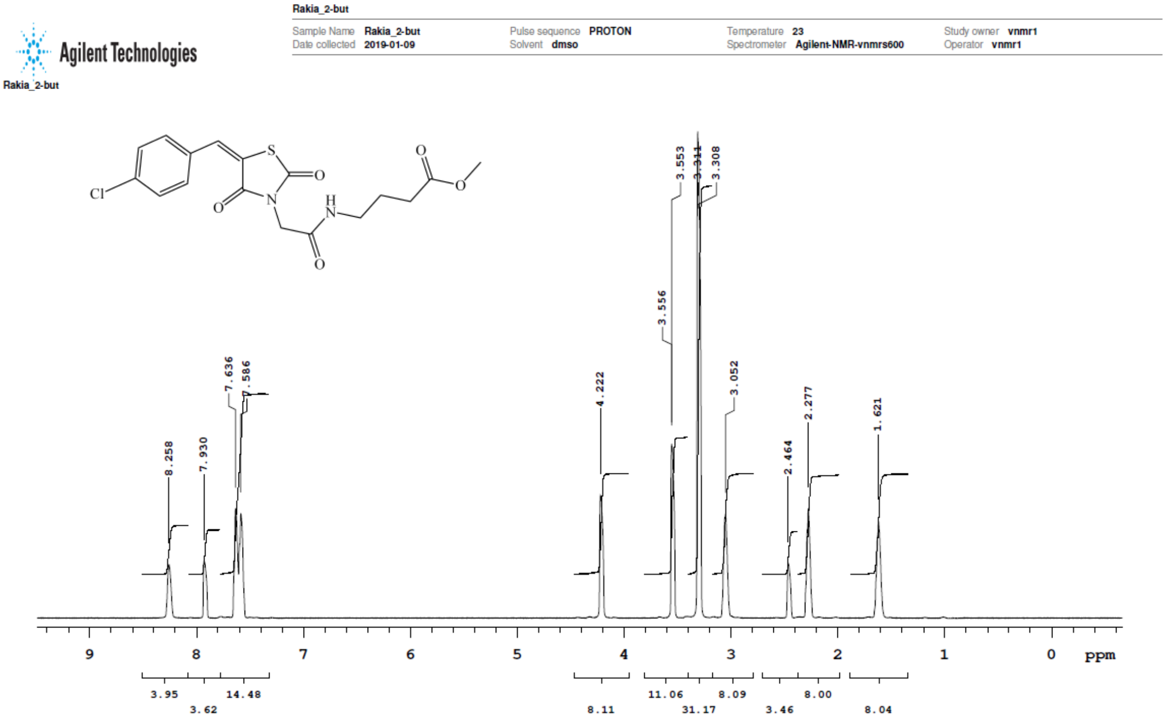

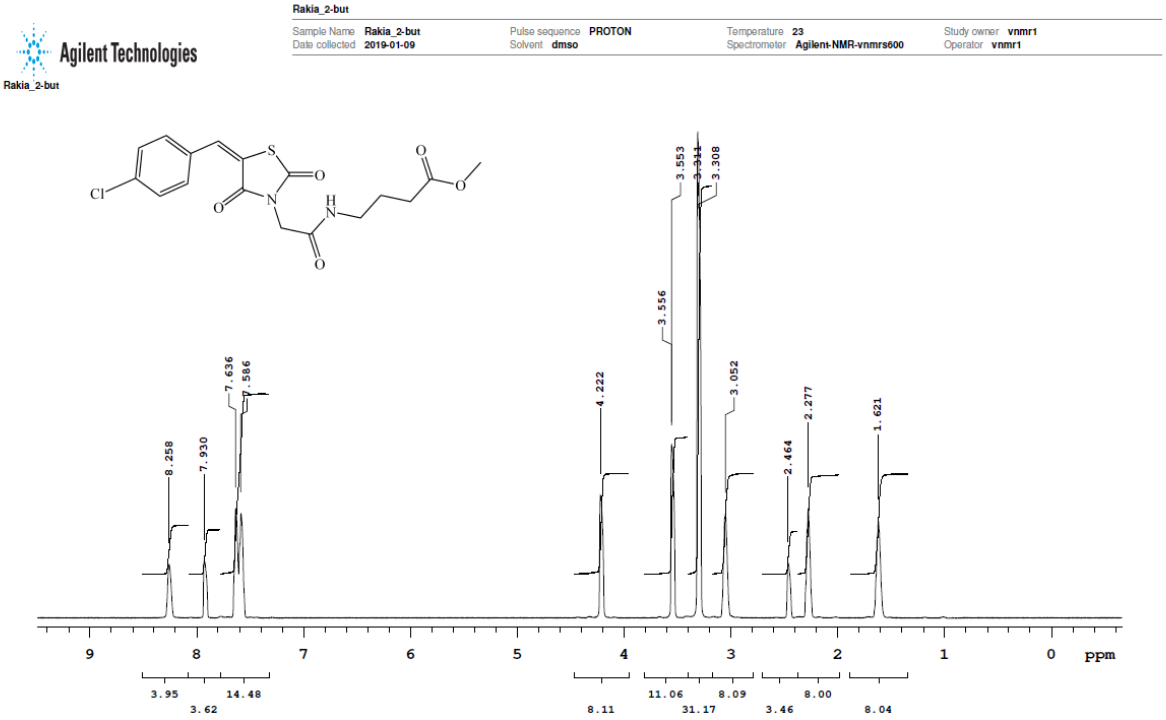

Figure S28 ^{1}H- and ^{13}C-NMR for compound **5f**

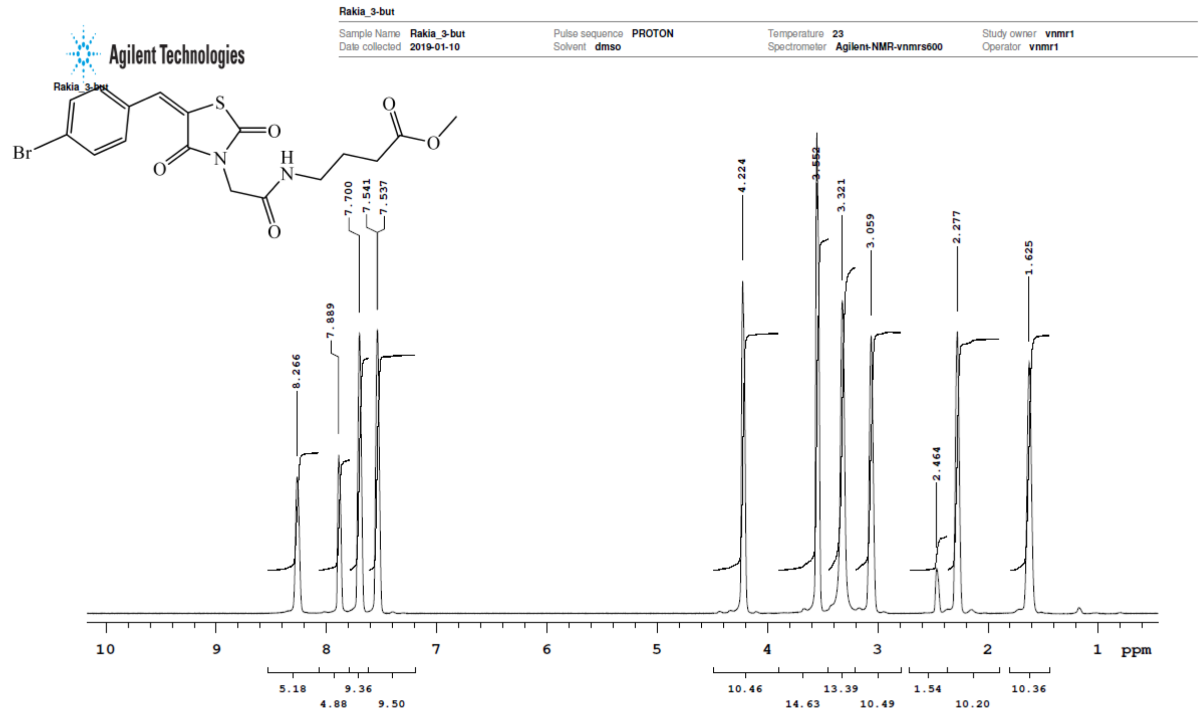

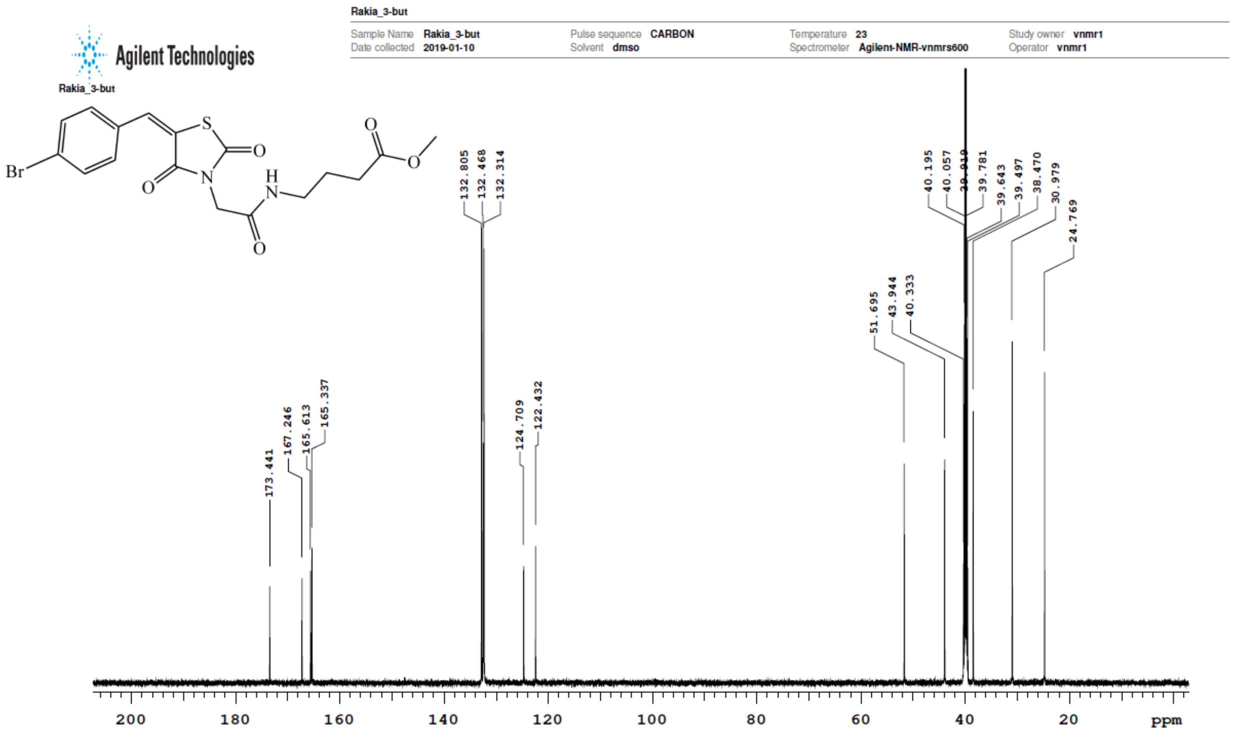

Figure S29 ^{1}H- and ^{13}C-NMR for compound **5g**

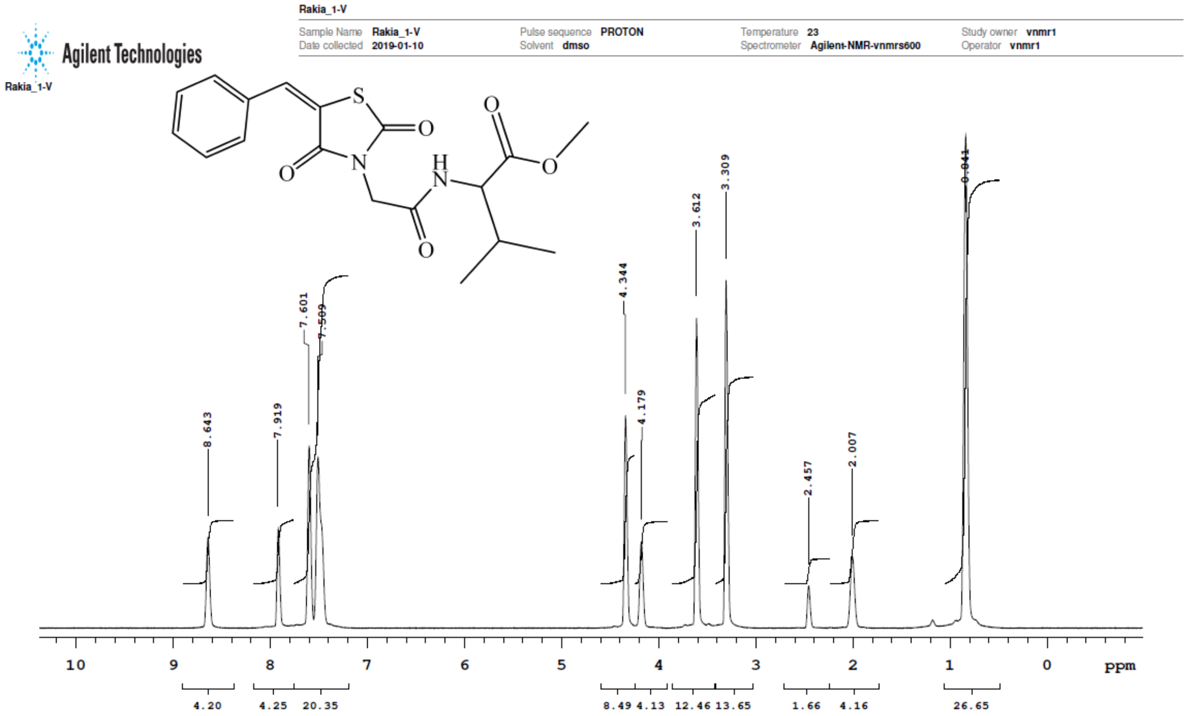

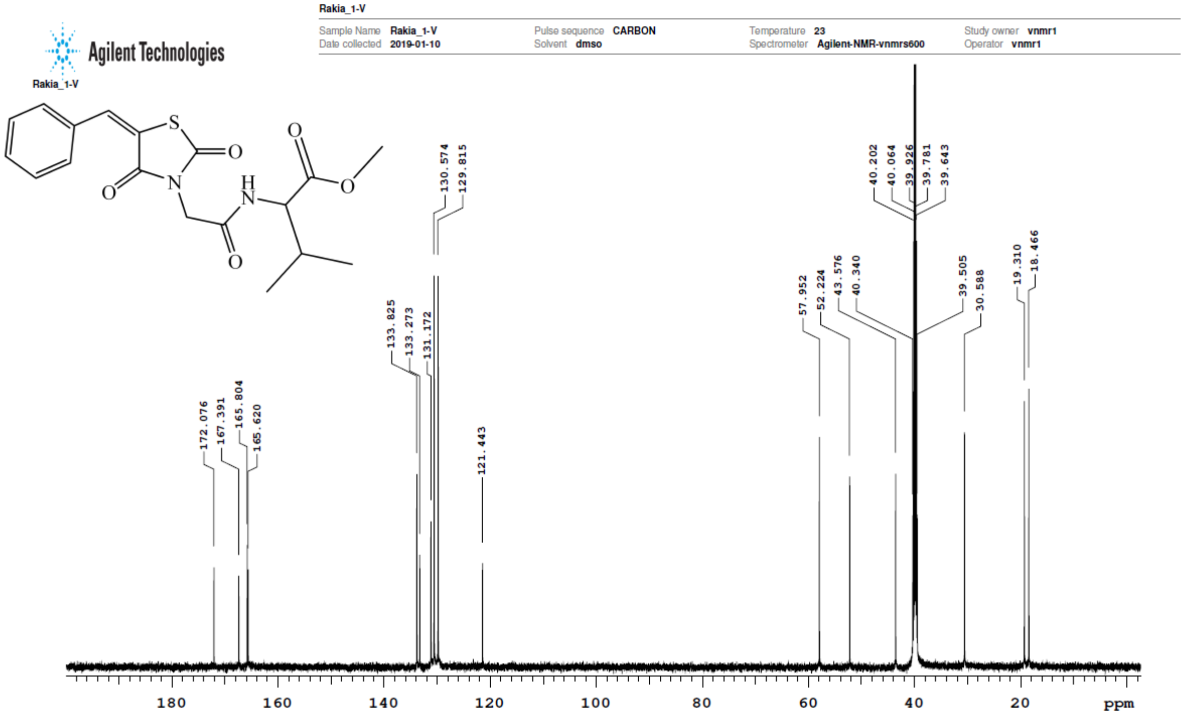

Figure S30 ^{1}H- and ^{13}C-NMR for compound **5h**

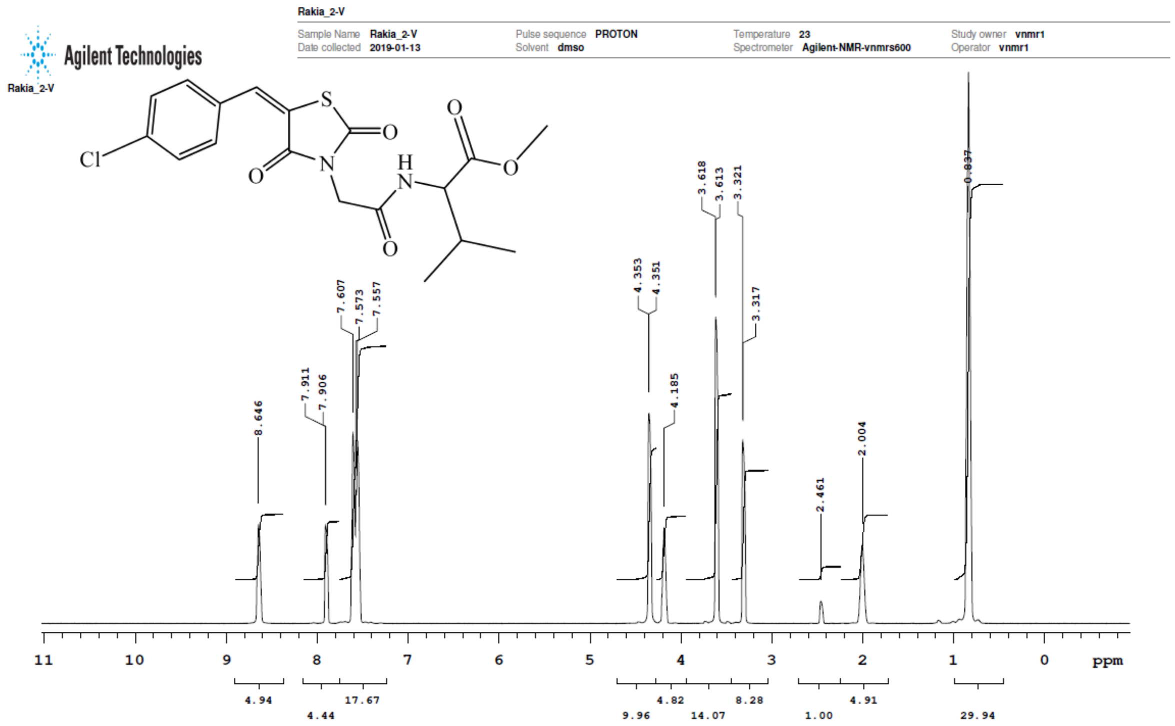

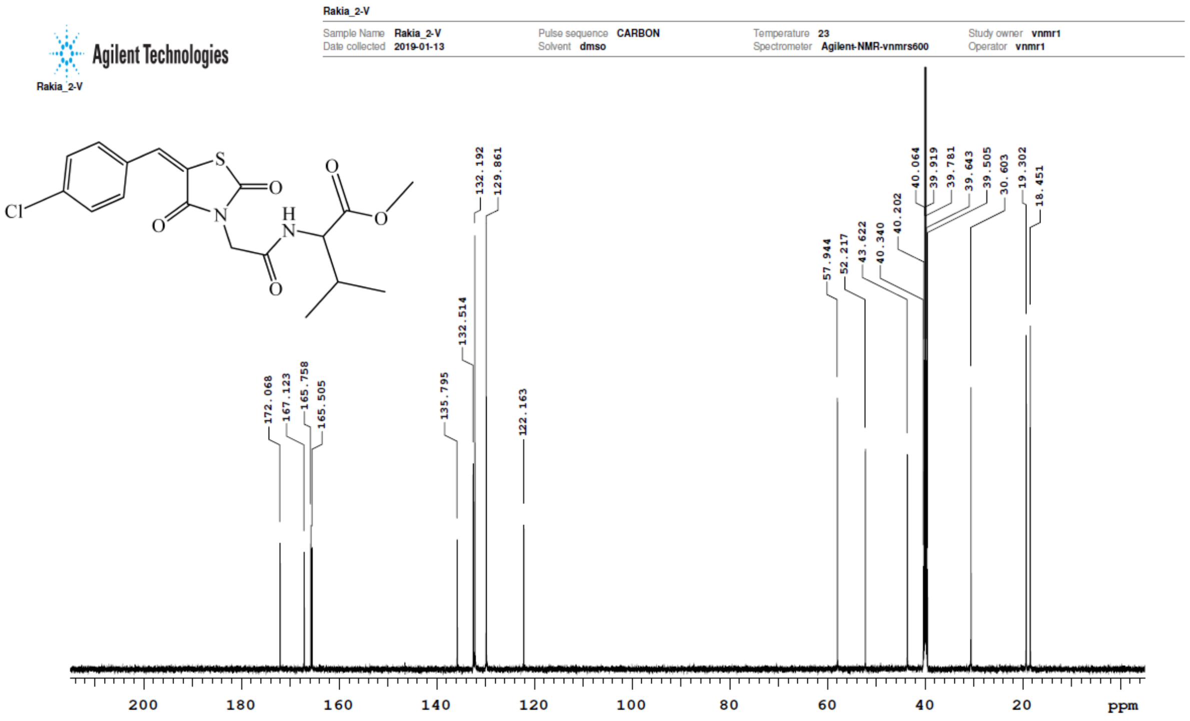

Figure S31 ^{1}H- and ^{13}C-NMR for compound **5i**

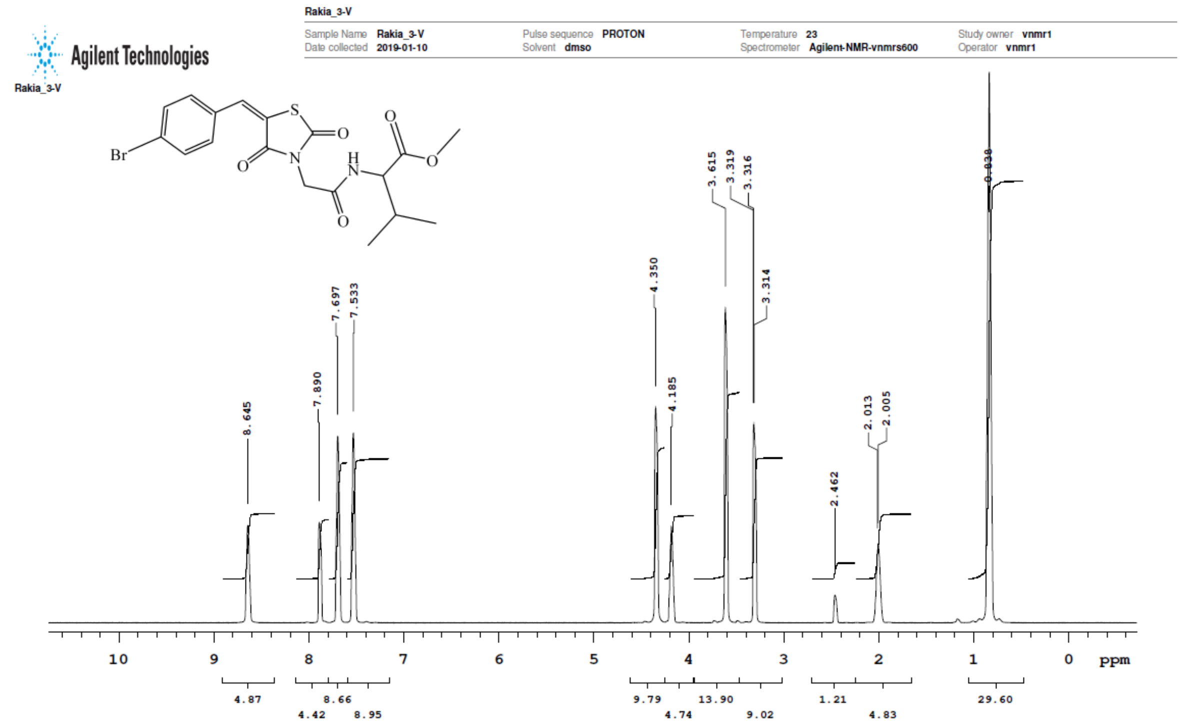

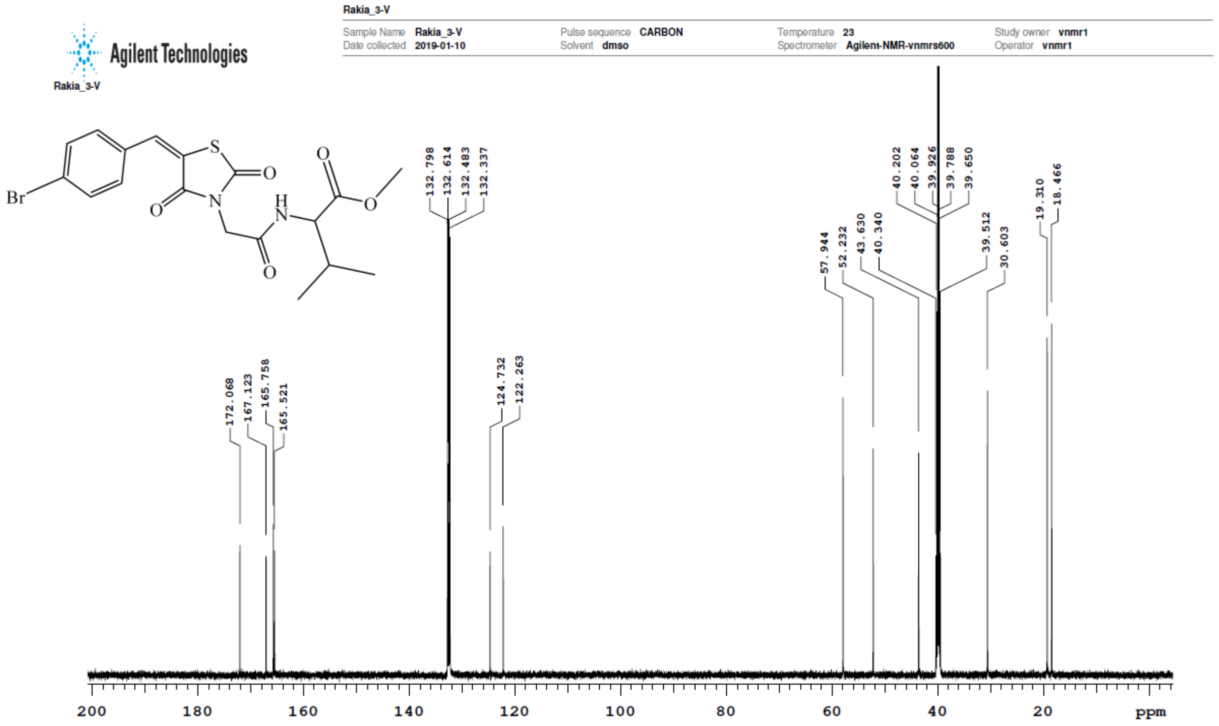

Figure S32 ^{1}H- and ^{13}C-NMR for compound **5j**

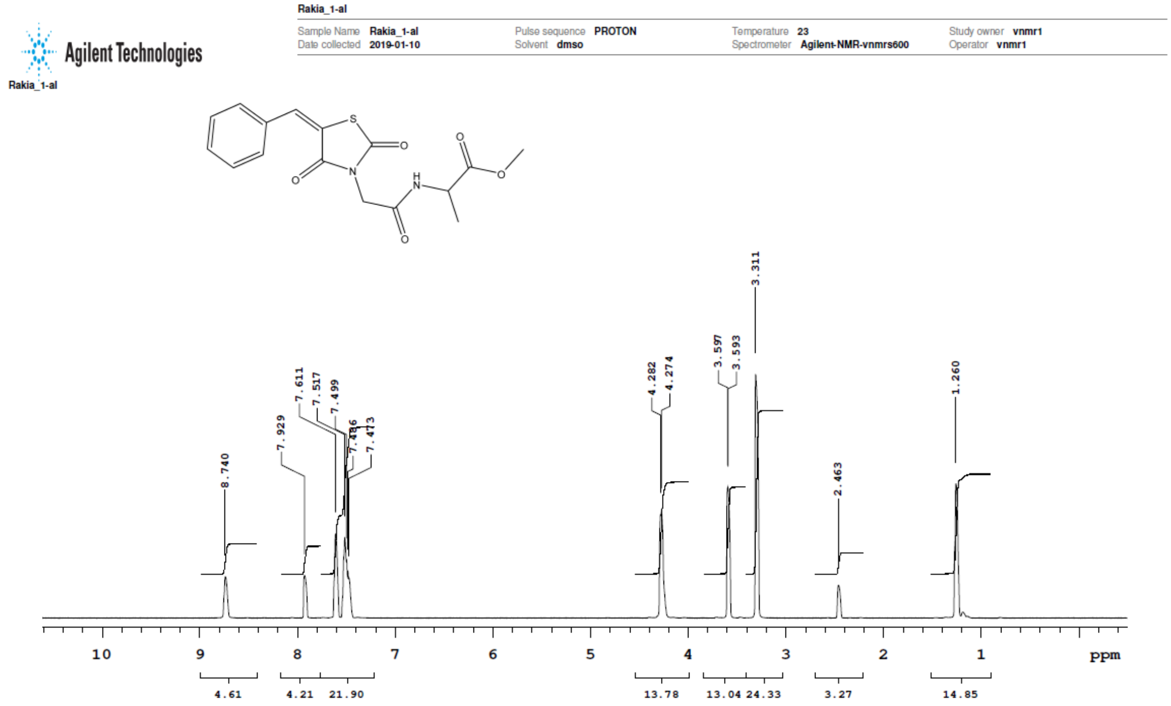

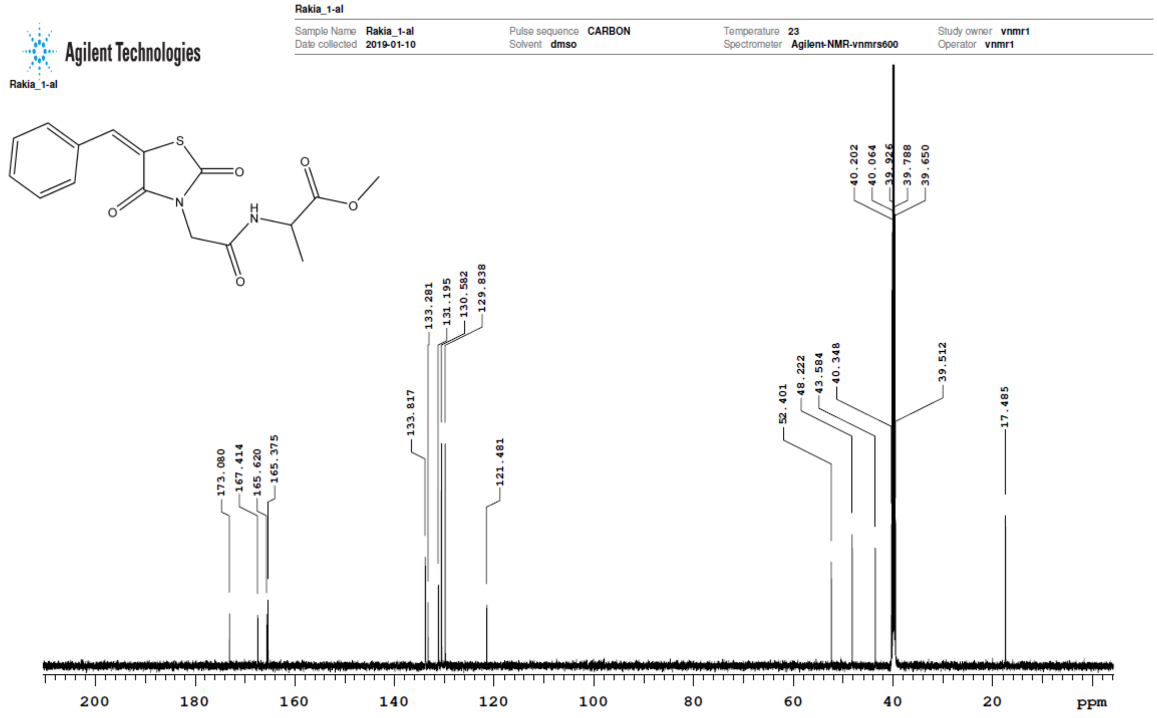

Figure S33 ^{1}H- and ^{13}C-NMR for compound **5k**

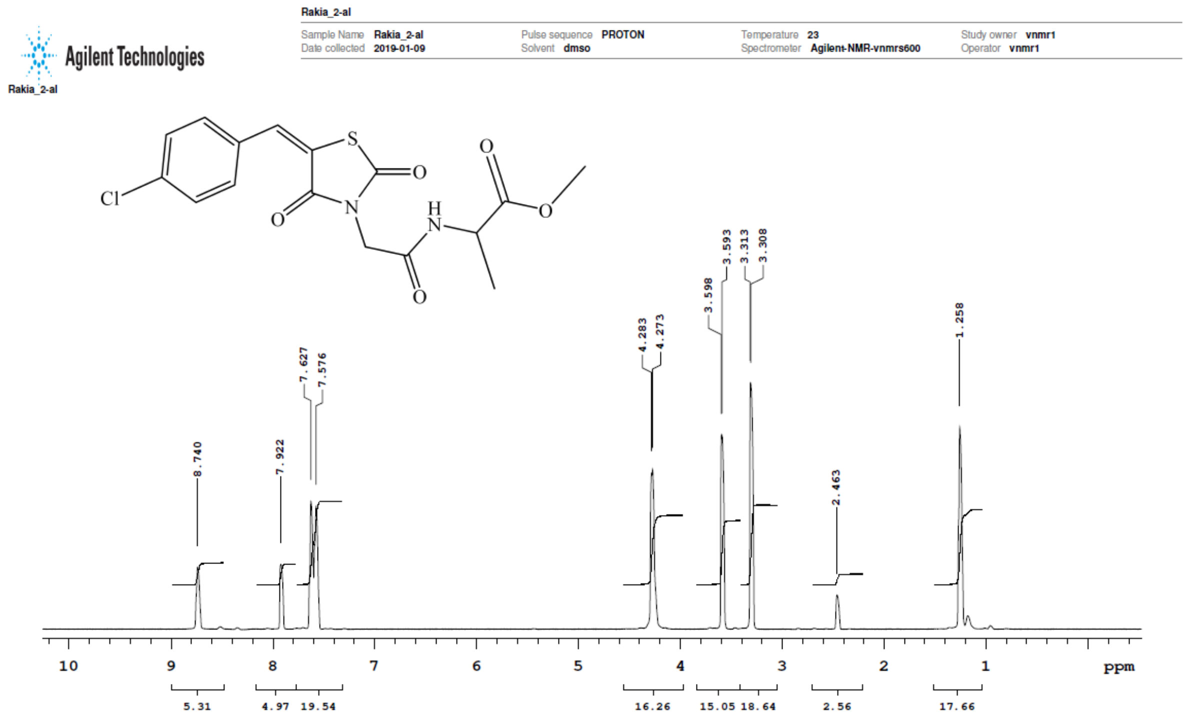

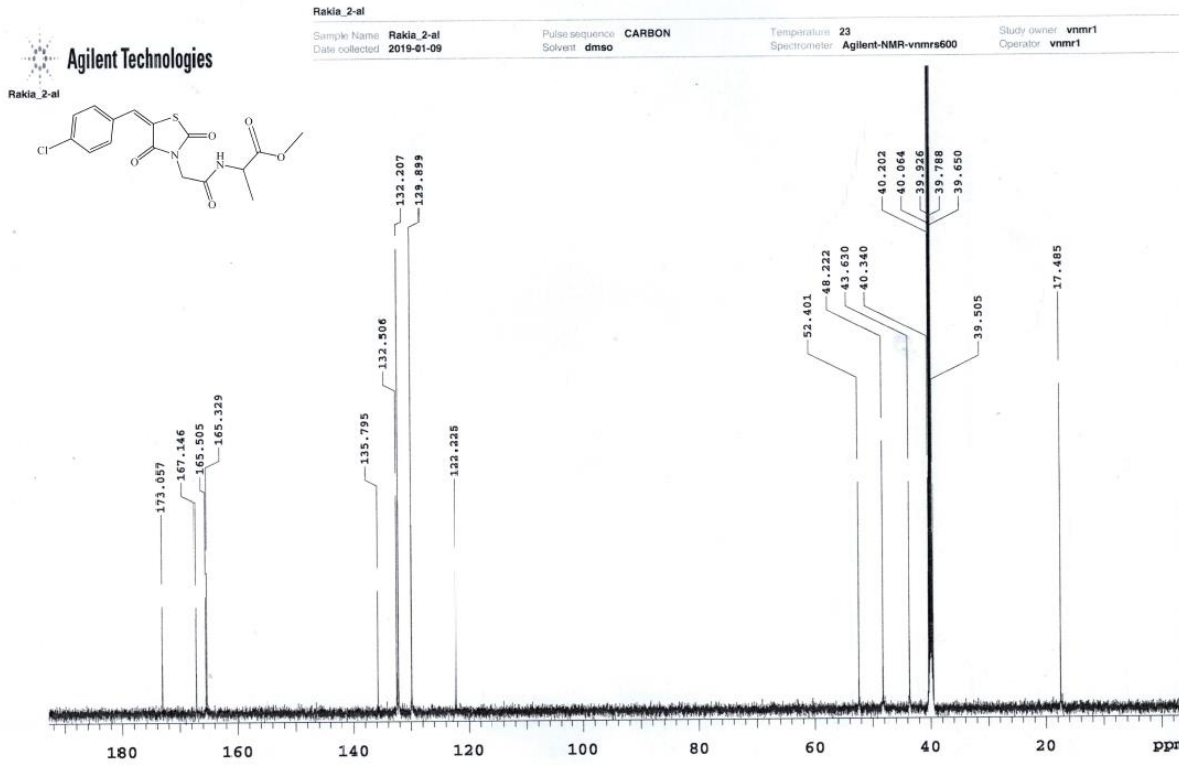

Figure S34 ^{1}H- and ^{13}C-NMR for compound **5l**

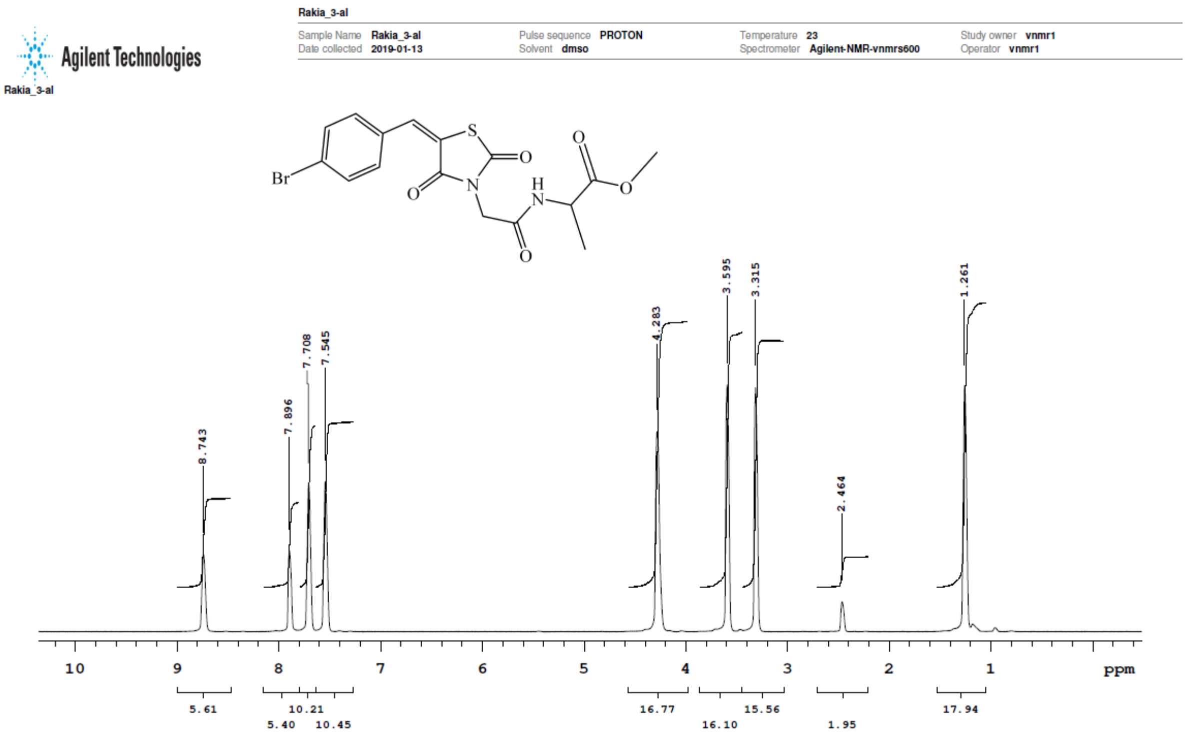

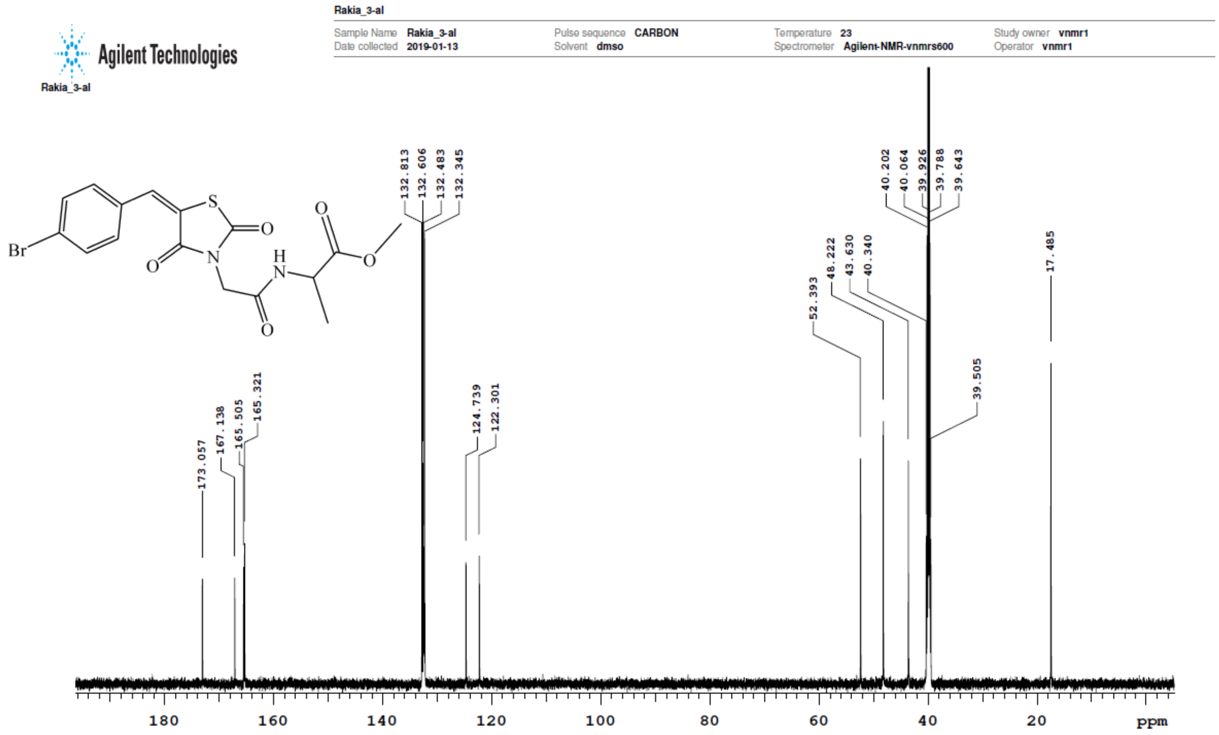

Figure S35 ^{1}H- and ^{13}C-NMR for compound **5m**

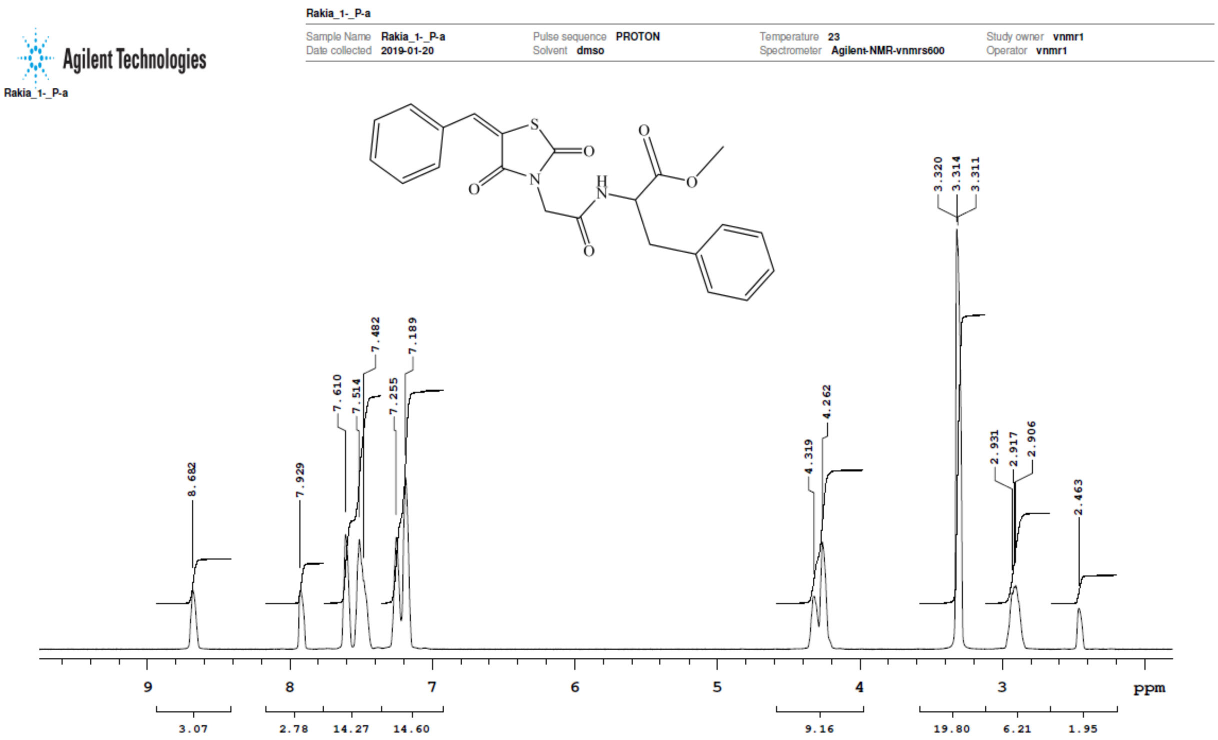

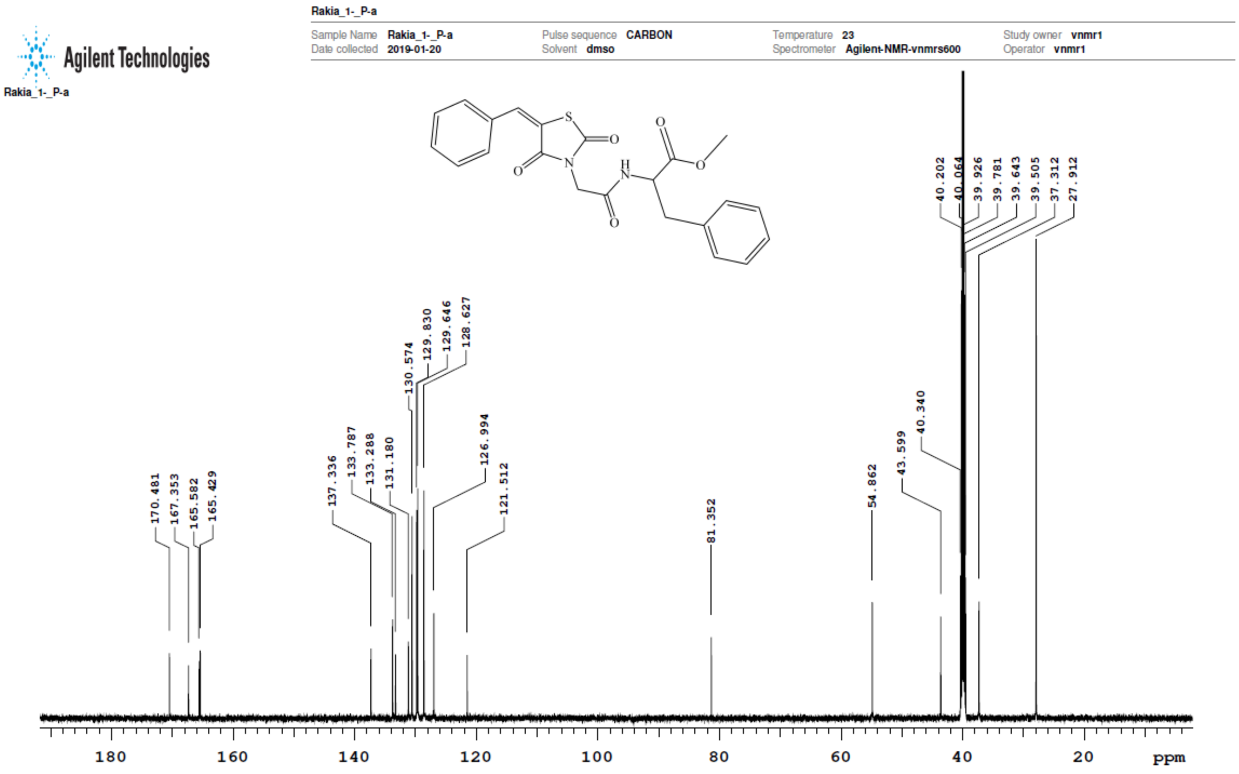

Figure S36 ^{1}H- and ^{13}C-NMR for compound **5n**

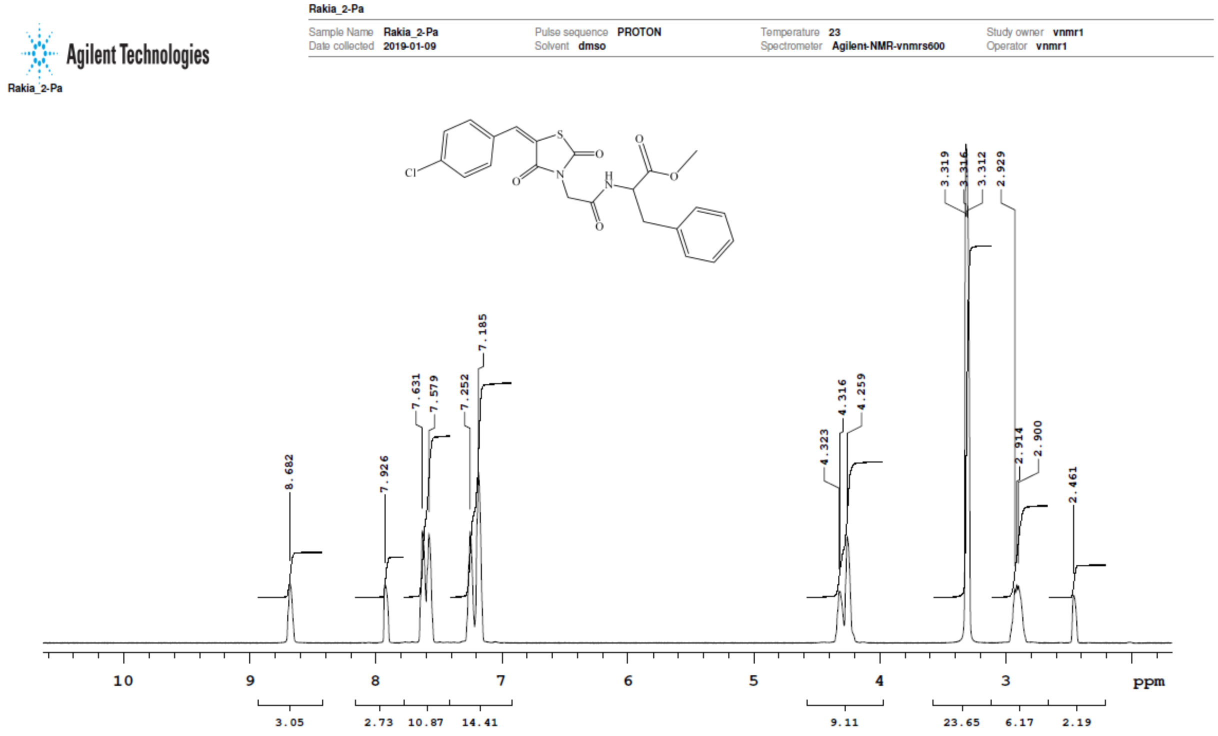

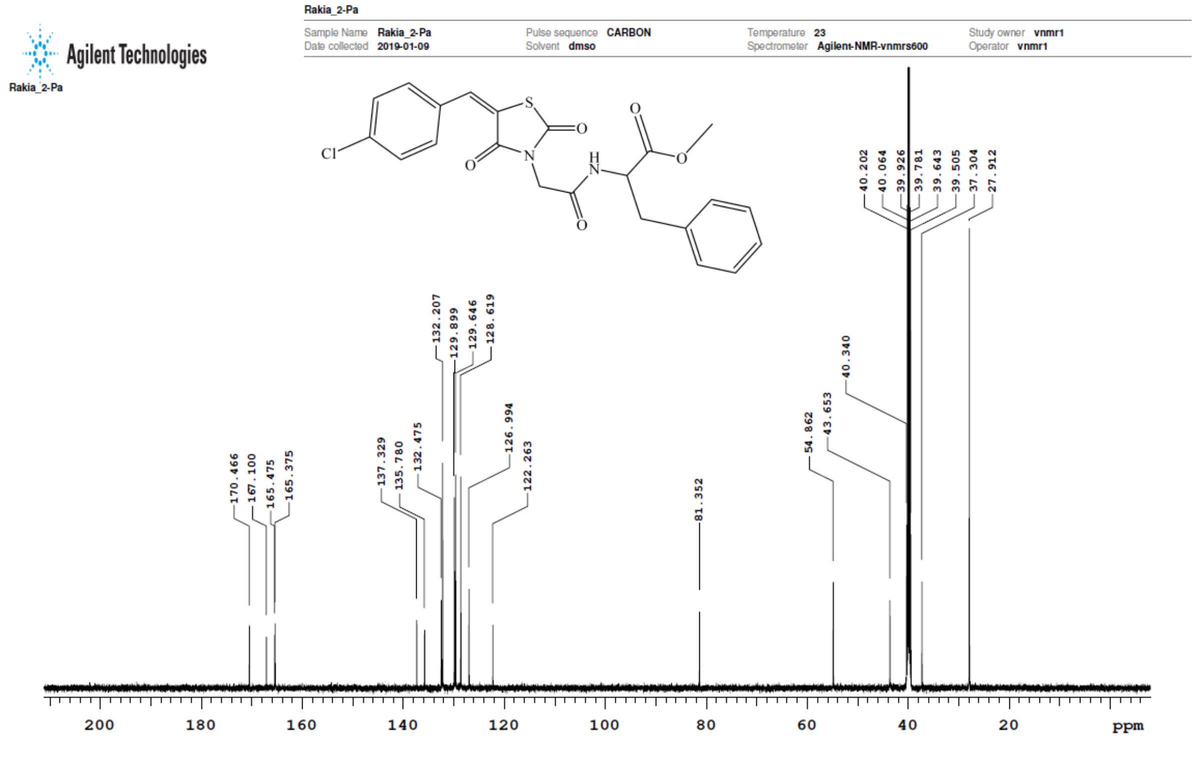

Figure S37 ^{1}H- and ^{13}C-NMR for compound **5o**

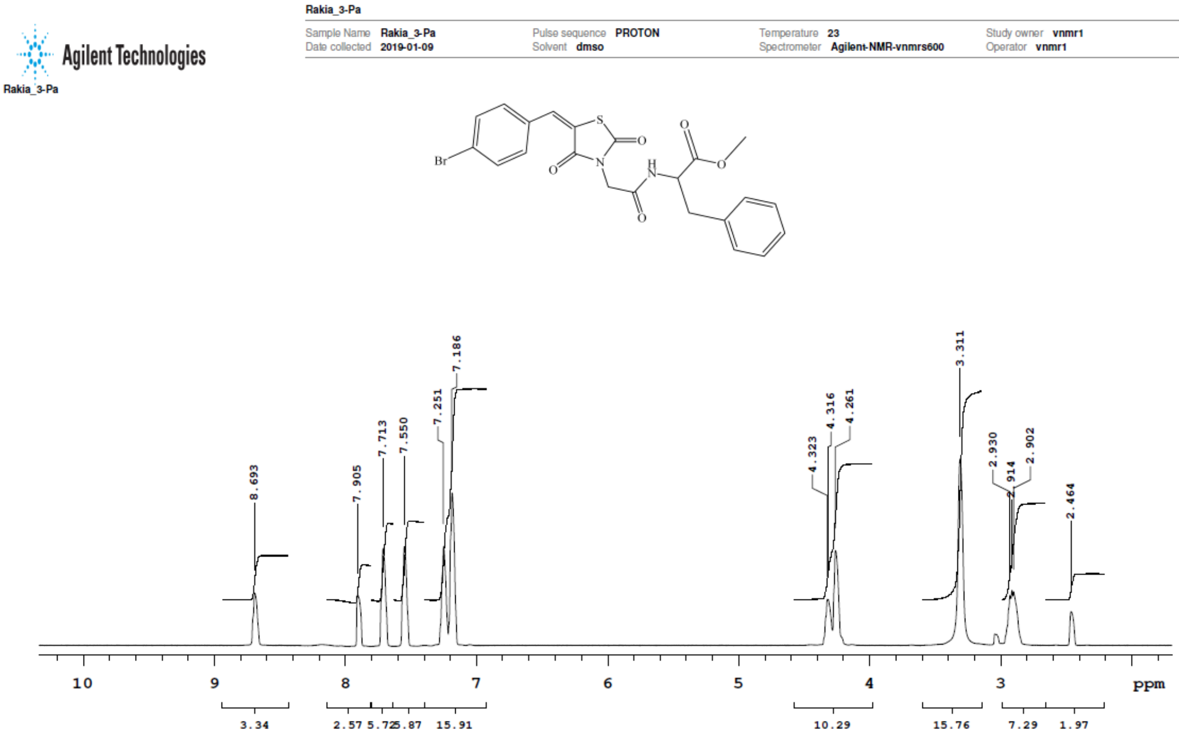

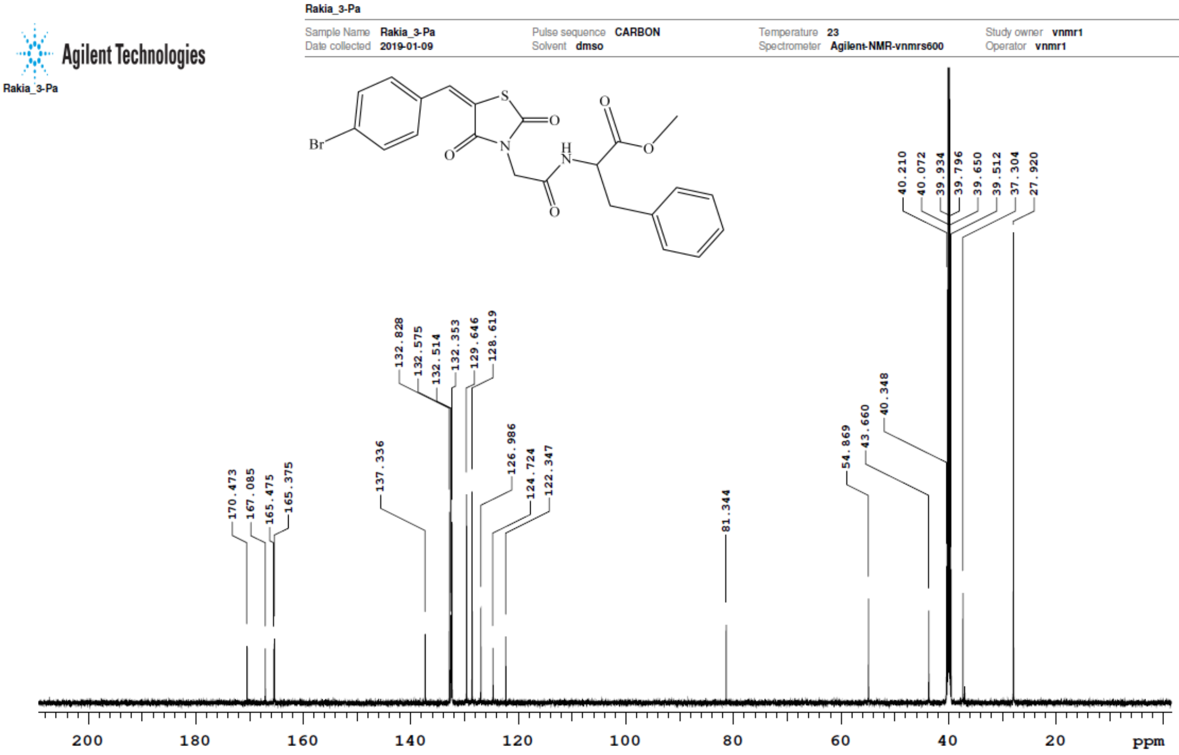

Supplement: Supplementary file 1 [file molecules-25-00105-s001.pdf]
